# Comparative genomics and evolutionary analyses of Sphaeropleales

**DOI:** 10.3389/fpls.2025.1534646

**Published:** 2025-10-16

**Authors:** Qian Xiong, Luqin Zheng, Qi Zhang, Tianli Li, Lingling Zheng, Lirong Song

**Affiliations:** ^1^ Institute of Hydrobiology, Chinese Academy of Sciences, Wuhan, China; ^2^ Center for Environment and Health in Water Source Area of South-to-North Water Diversion, School of Public Health, Hubei University of Medicine, Shiyan, China; ^3^ National Aquatic Biological Resource Center, Institute of Hydrobiology, Chinese Academy of Sciences, Wuhan, China; ^4^ University of Chinese Academy of Sciences, Beijing, China

**Keywords:** Sphaeropleales, comparative functional analysis, environmental adaptation, evolutionary analyses, gene family expansions and contraction

## Abstract

Sphaeropleales is a diverse group with over one thousand species described, which are found in a wide range of habitats showed strong environmental adaptability. This study presented comprehensive genomic analyses of seven newly sequenced Sphaeropleales strains with BUSCO completeness exceeding 90%, alongside comparative assessments with previously sequenced genomes. The genome sizes of Sphaeropleales species ranged from 39.8 Mb to 151.9 Mb, with most having a GC content around 56%. Orthologous analysis revealed unique gene families in each strain, comprising 2 to 3.5% of all genes. Comparative functional analysis indicated that transporters, genes encoding pyrroline-5-carboxylate reductase and antioxidant enzymes played a crucial role in adaptation to environmental stressors like salinity, cold, heavy metals and varying nutrient conditions. Additionally, Sphaeropleales species were found to be B_12_ auxotrophy, acquiring this vitamin or its precursors through a symbiotic relationship with bacteria. Phylogenetic studies based on 18S rDNA and the low copy othologues confirmed species identification and the relationships inside core Chlorophyta and between prasinophytes. Evolutionary analyses demonstrated all the species exhibited a large count of gene family expansions and contraction, with rapidly evolving and positive selected genes identified in terrestrial *Bracteacoccus* species, which contributed to their adaptation to terrestrial habitat. These findings enriched the genomic data for Sphaeropleales, particularly the genus *Bracteacoccus*, which can help in understanding the ecological adaptations, evolutionary relationships, and biotechnological applications of this group of algae.

## 1 Introduction

The Chlorophyta are a diverse group of green algae, which, together with the Streptophyta and Prasinodermophyta, belong to the Viridiplantae, an ancient lineage that diverged from a proposed ‘ancestral green flagellate’ (Fang et al., 2017; Leliaert et al., 2012; Li et al., 2020). Within the Chlorophyta, there are two major subdivisions: the core Chlorophyta and the paraphyletic prasinophytes. The monophyletic core Chlorophyta currently comprises five classes: Chlorophyceae, Ulvophyceae, Trebouxiophyceae, Pedinophyceae, and Chlorodendrophyceae (Fucikova et al., 2014; Sun et al., 2016; Turmel et al., 2016, 2017). The order Sphaeropleales belongs to the CS clade (which includes Chlamydomonadales and Sphaeropleales) of Chlorophyceae, a group containing 18 taxonomically recognized families. Over a thousand species have been described in this order, which are found in a wide range of habitats, demonstrating strong environmental adaptability and exhibiting diverse cellular organizations (Krienitz et al., 2011; Wolf et al., 2002).

Members of Sphaeropleales have been explored for bioassays and biofuel production, due to their potential for producing a range of biomolecules such as pigments, lipids, starch, and cellulose (Breuer et al., 2014, 2015, 2012, 2015; Chisti, 2007; De Jaeger et al., 2014; Rawat et al., 2013) Additionally, their rapid growth and strong resistance to environmental stress make them promising candidates for bioproduction. Some species are also valuable in ecological research and applications due to their heightened sensitivity to various substances compared to other algae (Nagai et al., 2016). For instance, species like *Raphidocelis subcapitata* and *Desmodesmus subspicatus* are recommended for ecotoxicological bioassays by the Organization for Economic Cooperation and Development (OECD) (TG201, http://www.oecd.org/) (Suzuki et al., 2018).

The rapid advancements in genome sequencing over the past two decades have made comparative genomics a key approach in biological research, which is instrumental in uncovering the origin and function of genes and gene families, as well as understanding the mechanisms that drive complexity and diversification during evolution (Goodwin et al., 2016). And comparative genomics has increasingly been applied to the study of eukaryotic algae, including *Chlamydomonas* (Chlorophyta) (Craig et al., 2021), *Cladosiphon okamuranus* (a brown alga) (Nishitsuji et al., 2020), diatoms (Jeong and Lee, 2024), red algae (Ho, 2020).

To date, 28 Sphaeropleales genomes have been sequenced and are available in public databases. Of these, 20 strains belong to four genera within the Scenedesmaceae, six strains are from three genera within the Selenastraceae, and additional genomes are available from Mychonastaceae and Scenedesmaceae. Most of these genomes have been assembled to the contig or scaffold level, with genome sizes ranging from 24 Mb to 208 Mb and contig N50 values ranging from 2.8 Kb to 6125 Kb. Among these 28 genomes, only eight have been annotated, including those of *Monoraphidium neglectum*, *Monoraphidium minutum*, *Raphidocelis subcapitata*, *Scenedesmus* sp. *NREL 46B-D3*, and two *Tetradesmus obliquus* strains (Bogen et al., 2013; Carreres et al., 2017; Dasgupta et al., 2018; Demirbas, 2011; Suzuki et al., 2018). The predicted gene counts for these species range from 7,092 to 17,867 genes. These sequenced Sphaeropleales genomes provide valuable insights into the biosynthesis of lipids and pigments, including triacylglycerol (TAG) and carotenoids, making these species suitable for biotechnological applications and production. Comparative genomics analyses have also revealed mechanisms underlying environmental adaptation, such as the capacity for both heterotrophic and mixotrophic lifestyles, as well as tolerance to salinity and low metal concentrations.

Nucleotide substitution rates are often used as the criterion to reflect selection pressure. While nonsynonymous substitution rates (dN) can cause amino acid change, synonymous substitution rates (dS) do not cause amino acid change. The dN/dS ratio is the measure of natural selection acting on the protein. According to Yang (Yang, 2007), dN/dS < 1 denotes negative purifying selection, dN/dS = 1 signifies neutral evolution, and dN/dS > 1 indicates positive selection (Xiong et al., 2021). As most of the plastid protein-coding genes undergo negative or purifying selection to maintain their function, they are conserved and have a low dN/dS ratio. However, some genes might undergo positive selection in response to environmental changes, consequently presenting relatively high dN/dS ratio (Henriquez et al., 2020; Iram et al., 2019; Smith, 2015).

In this study, we sequenced the genomes of seven Sphaeropleales strains, including the first two terrestrial *Bracteacoccus* species, and performed an in-depth analysis of these genomes together with the seven previously reported high-quality Sphaeropleales genomes. Functional annotation-based comparative genomic analysis revealed key insights into the environmental adaptations of this group. Phylogenetic and evolutionary analyses, based on gene families and low-copy orthologues, showed extensive gene family expansions and contractions across all species. Rapidly evolving and positively selected genes were identified in the terrestrial *Bracteacoccus* species, which contributed to the adaption to the terrestrial habitat. The findings of this study provide valuable information for understanding the environmental adaptations and evolutionary relationships within Sphaeropleales.

## 2 Materials and methods

### 2.1 Sampling, culture conditions, DNA extraction, library preparation, sequencing, genome assembly, and cleaning of the reads

We obtained seven Sphaeropleales strains from National Aquatic Biological Resource Center, Institute of Hydrobiology, Chinese Academy of Sciences. The lists were *Bracteacoccus aerius* (FACHB-895), *Bracteacoccus engadinensis* (FACHB-1300), four *Tetradesmus obliquus* strains (FACHB-14, FACHB-276, FACHB-417 and FACHB-1235), *Monoraphidium contortum* (FACHB-3677). *Bracteacoccus aerius* and *Bracteacoccus engadinensis* were terrestrial, and others were collected from freshwater. All these strains were grown at 25 °C in liquid BG11 medium under a 12/12-h light/dark cycle.

DNA was extracted using a Universal DNA Isolation Kit (Axygen, Suzhou, China). A NEB Next Ultra DNA Library Prep Kit for Illumina (New England Biolabs, Ipswich, Massachusetts, USA) was used for preparing sequencing libraries which were sequenced on an DNBSEQ platform. The quality of the raw reads was initially assessed using FastQC v0.11.6 (http://www.bioinformatics.babraham.ac.uk/projects/fastqc/ accessed on 24 June 2021). Data were trimmed using SOAPnuke software (Chen et al., 2017) and were then assembled by megahit v 1.0.3 (Li et al., 2015) with defaulted parameters. Anvio 7.0 (Eren et al., 2015) was used to remove prokaryotic sequences from the assembled genomes with defaulted parameters. Firstly, Bowtie2 (Langmead and Salzberg, 2012) was used to align the assembled genomes to the fq files, generating BAM files. Then, the ‘anvi-gen-contigs-database’ was employed to generate a contig database, followed by the use of ‘anvi-run-hmms’ to identify single-copy genes in the contig database. The ‘anvi-profile’ was used to import sample information into the database. Next, ‘anvi-interactive’ was used to visualize the results. Finally, ‘anvi-export-collection’ was used to export the eukaryotic bins, and ‘anvi-export-contigs’ was used to extract the sequences of the aimed bins, resulting in the final assembled genomes. The genomes of the seven Sphaeropleales trains were deposited in CNCB with accession numbers SAMC4339295 - SAMC4339301, respectively.

### 2.2 Gene prediction, genome annotation and evaluation

*Ab initio* gene prediction was performed by AUGUSTUS version 3.2.1 (Stanke et al., 2006) with four three-trained organism models: *volvox*, chlamy2011, and *chlorella*. GeMoMa-1.9 (Keilwagen et al., 2018) was employed for homolog-based gene prediction and integration of gene prediction results, with genomes and protein of *Volvox carteri* f. *nagariensis* (GCA_000143455.1), *Gonium pectorale* (GCA_001584585.1), *Haematococcus lacustris* (GCA_030144725.1), and *Tetradesmus obliquus* (GCA_030272155.1) from GenBank as the references. Functional annotation of predicted proteins was performed by InterProScan (Jones et al., 2014) and EggNOG-mapper (Huerta-Cepas et al., 2017) for GO term mapping and KEGG pathway analyses respectively. To assess the completeness of the genome assembly and annotation, BUSCO (Simao et al., 2015) (Benchmarking Universal Single-Copy Orthologs) was used, utilizing the chlorophyta_odb10 database for a quantitative evaluation.

### 2.3 Orthologous gene estimation, transporters identification and repeat composition

Orthologous gene groups for the Sphaeropleales samples (including those newly added in this study and those available in the public database with annotation completeness greater than 80%) were estimated using Orthofinder (Emms and Kelly, 2015) with default parameters to identify single-copy or low-copy orthologues. Additionally, *V. carteri* f. *nagariensis* (GCA_000143455.1) and *D. salina* (GCA_002284615.2) were included as outgroups to estimate shared orthologues for subsequent divergence time estimation and gene family evolution analysis. Transporters were identified using BLASTp, with the Transporter Classification Database (TCDB) as a query, with an e-value cut-off of <1E^−5^. Further, repeat content for each genome was determined by RepeatModeler and RepeatMasker with default parameters. A library of repeats was first created for each assembly using RepeatModeler (Version 2.0.1) and all repeats were masked using RepeatMasker (Version 4.1.0).

### 2.4 Phylogenomic analyses

The predicted protein sequences (PEP) sequences of each single-copy or low-copy orthologue were aligned using MAFFT v7.394 (Katoh and Standley, 2013) with the parameters -maxiterate 1000 and -globalpair. Regions with poor alignment were trimmed using TrimAl v1.2 (Capella-Gutierrez et al., 2009) with the -automated1 option. The resulting trimmed alignments of orthologous groups were then used for subsequent phylogenomic analysis. Coalescent-based analyses were employed to construct the phylogenetic tree. For these analyses, RAxML (Stamatakis, 2014) was used to perform maximum likelihood (ML) analysis of each single-copy orthologue, applying the PROTGAMMA GTR model. Additionally, ASTRAL (Zhang et al., 2018) was used to infer the coalescent-based species tree (ST) phylogeny.

The 18S rDNA sequences were aligned using MAFFT v7.0 (Katoh and Standley, 2013), and ambiguous regions were manually adjusted and refined using MEGA7 (Kumar et al., 2016). To locate the positions of 18S rDNA sequences within the sequenced genomes, nhmmer (Wheeler and Eddy, 2013) was used to align the final assembled genomes to the rDNA database, based on rDNA sequences downloaded from NCBI. Subsequently, SSU sequences were extracted using Perl scripts. To understand the phylogenetic relationships of the strains in this study, additional Sphaeropleales 18S rDNA sequences were downloaded from the public database. The 18S rDNA sequences were analyzed using jModelTest2 (Darriba et al., 2012) to select the best-fit model, which was found to be GTR + I + G. Bayesian inference (BI) method was applied to infer the phylogeny.

### 2.5 Gene family expansion and contraction estimation

PhyloSuite (Zhang et al., 2020) was used to concatenate all the shared single-copy orthologous groups from the Sphaeropleales and the outgroup, resulting in a concatenated sequence of 35,684 amino acids. This concatenated alignment, along with the species tree constructed by ASTRAL (Zhang et al., 2018), was used for Bayesian divergence time estimation using the approximate likelihood method described by dos Reis & Yang, implemented in MCMCtree v. 4.9 (Yang, 2007).

The number of gene copies per family, as determined by Orthofinder (Emms and Kelly, 2015), and the timetree estimated earlier by MCMCtree (Yang, 2007). were used to analyze gene family expansion and contraction using CAFE v. 5.1 (Mendes et al., 2020). Expanded and contracted genes were then extracted for Gene Ontology (GO) functional and KEGG pathway enrichment analyses. For GO enrichment, all PEP sequences were imported into InterProScan (Jones et al., 2014) for GO term mapping. The analysis was carried out using the clusterProfiler (Yu et al., 2012) package, with a significance cutoff of *p* < 0.05, and the false discovery rate (FDR) method was applied to adjust for multiple testing (Benjamini and Hochberg, 1995).

### 2.6 Evolutionary analysis

The CODEML program of PAML v4.9 (Yang, 2007) was used to estimate positive selection and rapidly evolving genes based on common orthologues were described as Xiong et al (Xiong et al., 2022, 2021). The branch model was employed in the calculation of dN/dS ratios for terrestrial Sphaeropleales species and aquatic ones with the two *Bracteacoccus* species labeled as foreground branches. A null model (model = 0), where one dN/dS ratio was fixed across all strains, was compared with an alternative model (model = 2), where *Bracteacoccus* species were allowed to have a different dN/dS ratio. Likelihood ratio tests were performed to examine model fit, a chi-squared test was used to analyze *p* values, and multiple testing was corrected using false discovery rate (FDR) in R. The genes were considered putative rapidly evolving genes if they had an FDR-adjusted *p* value < 0.05 and a higher dN/dS ratio in the foreground branch than in the background branches.

Branch-site model was utilized to find genes that possibly underwent positive selection. The improved branch-site model (model = 2, Nsites = 0) was used to detect signatures of positive selection on individual amino acids in a specific branch. The two *Bracteacoccus* species were set as the foreground branch. The null model assumed that no positive selection occurred on the foreground branch (fix_omega = 1, omega = 1), and the alternative model assumed that sites on the foreground branch were under positive selection (fix_omega = 0, omega = 1.5). LRT were used to test model fit and Chi-square test was applied for examining the *P* values in R. A correction was performed for multiple testing using an FDR criterion, and BEB method was employed to statistically identify sites under positive selection. Genes with an FDR-adjusted *P* < 0.05 were considered as putatively selected. For the genes belonging to both under positive selection and putative rapidly evolving were performed for gene ontology (GO) functional enrichment analyses as above.

## 3 Results and discussion

### 3.1 Phylogenetic analyses

**Figure 1:**
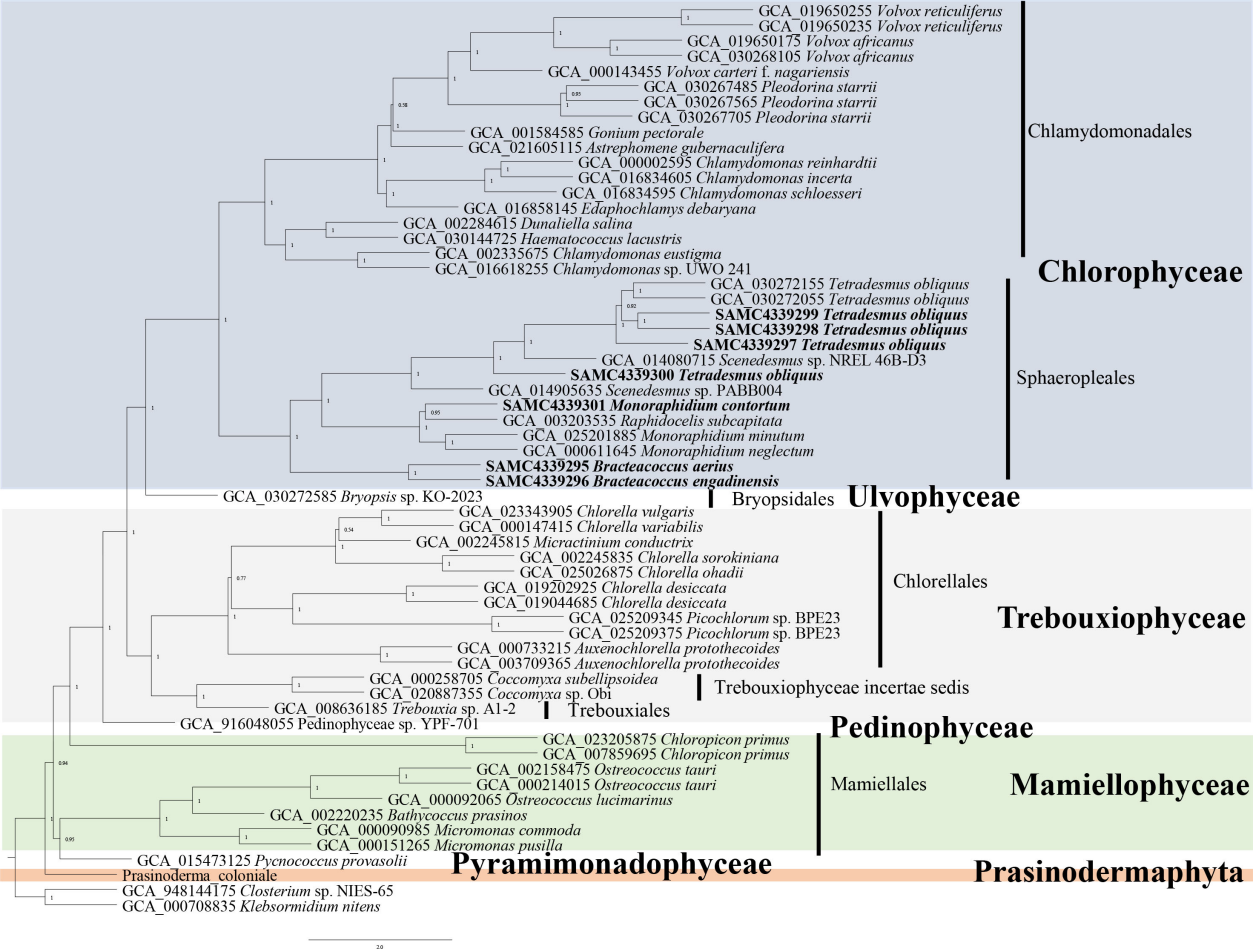
Phylogenetic tree of the Chlorophyta based on the low-copy orthologues to by ASTRAL. Numbers on branches represent support values of ASTRAL. Branch lengths are proportional to genetic distances, which are indicated by the scale bar. The species in bold indicates the newly added Sphaeropleales strains in this study.

The seven newly added Sphaeropleales strains were collected prior to 2000 and were deposited in National Aquatic Biological Resource Center, Institute of Hydrobiology, Chinese Academy of Sciences. Phylogenetic analysis based on 18S rDNA sequences extracted from the genomes was performed, and the results are shown in **Supplementary Figure S1**. The phylogenetic tree with high support values at the basal nodes confirmed species identification. We also performed a phylogenetic analysis using low-copy orthologues across the entire Chlorophyta, including core Chlorophyta and prasinophytes by Astral (**Figure 1**), which enables highly accurate phylogenomic estimation, even in the presence of high levels of gene tree conflict because of incomplete lineage sorting (Mirarab et al., 2014) or horizontal gene transfer (Davidson et al., 2015). The results were almost identical with the previous studies, supporting the phylogenetic relationships within core Chlorophyta and between prasinophytes. Notably, our analysis confirmed that *P. coloniale* (CCMP 1413) was distinct from both core Chlorophyta and prasinophytes, suggesting it belongs to a newly identified phylum, Prasinodermophyta (Li et al., 2020).

### 3.2 Genome sequencing and characteristics

Using Illumina platforms, we sequenced and assembled draft genomes for *B. aerius*, *B. engadinensis*, *M. contortum*, *S. obliquus*, and three *T. obliquus* strains. These genomes, along with seven previously sequenced Sphaeropleales genomes, were summarized in **Table 1**. The contig N50 values of the seven newly sequenced genomes were nearly identical, ranging from 20.5 to 22.34 kb. The BUSCO completeness for all genomes exceeded 90%, indicating that the genome assemblies were sufficiently complete for comparative analyses of genomic and gene constituents. The genome sizes of the seven sequenced species ranged from 45.62 Mb to 109.8 Mb, with the GC content varied from 55.72% to 57.63%. Gene counts varied significantly among species, ranging from 6,874 to 13,568. The repeat composition covered 3.06% to 18,88% of the genome.

**Table 1:** Comparison of draft genome assemblies of seven species of Sphaeropleales algae.

Species	Assembled genome size (Mb)	Number of contigs	N50 contig length (kp)	Number of scaffolds	N50 scaffold length (kb)	Genome complete BUSCOs	Number of genes	GC (%)	Repeated (%)	Accession number
*Bracteacoccus aerius*	69.48	5602	22.164	5602	22.164	90.80%	7612	56.68	3.27	SAMC4339295
*Bracteacoccus engadinensis*	62.94	3570	21.575	3570	21.575	92.50%	7697	55.72	3.06	SAMC4339296
*Scenedesmus obliquus*	96.20	5611	22.336	5611	22.336	91.30%	12827	56.99	10.95	SAMC4339297
*Tetradesmus obliquus*	101.41	5722	22.183	5722	22.183	93.70%	13568	56.85	18.88	SAMC4339298
*Tetradesmus obliquus*	95.24	5163	22.128	5163	22.128	92.50%	13152	57.01	11.56	SAMC4339299
*Tetradesmus obliquus*	109.80	11643	20.503	11643	20.503	90.30%	11717	56.15	6.78	SAMC4339300
*Monoraphidium contortum*	45.62	2569	22.204	2569	22.204	91.60%	6.874	57.63	3.75	SAMC4339301
*Monoraphidium minutum*	68.2	512	259.4	511	259.4	95.6%	15464	72.00	73.72	GCA_025201885.1
*Monoraphidium neglectum*	69.50	12074	9.1	6718	15.6	90.9%	16,807	65.00	4.26	GCA_000611645.1
*Raphidocelis subcapitata*	51.20	1,620	91.8	300	341.8	94.6%	13,429	71.50	1.38	GCA_003203535.1
Scenedesmus sp. NREL 46B-D3	151.90	2,661	204.9	–	–	94.9%	17,867	57.50	7.67	GCA_014080715.1
*Scenedesmus* sp. PABB004	39.8	77	1300	77	1300	96%	7,092	78.50	1.31	GCA_014905635.1
*Tetradesmus obliquus*	100.30	17	600	17	600	94.1%	14,673	57.00	11.8	GCA_030272055.1
*Tetradesmus obliquus*	104.70	17	6100	17	6100	95.7%	15224	56.50	10.03	GCA_030272155.1

Among all the Sphaeropleales species, the genomes size of *Scenedesmus* species exhibited significant variation, ranging from 39.8 Mb to 151.90 Mb, representing both the smallest and largest genomes within this group. In contrast, *Monoraphidium*, *Raphidocelis*, and *Bracteacoccus* displayed moderate genome sizes, ranging from 46.6 Mb to 69.5 Mb. And *Tetradesmus* species possessed relatively larger genomes that exceed 100 Mb. Most species have a GC content around 56%, exception for *Monoraphidium* and *Scenedesmus* sp. PABB004 greater than 70%. The repeat content varied considerably among all genomes examined, *Scenedesmus* sp. PABB004 and *R. subcapitata* showed the lowest repeat content about 1.3%, and *T. obliquus* (SAMC4339298) displayed the highest at 18.8%. *Bracteacoccus* and *Monoraphidium* have moderate repeat content, ranging from 3% to 4%, whereas *Tetradesmus* and most *Scenedesmus* species exhibit higher ranging 6.78% to 18.88%. *minutum*,

It is generally accepted that there is some degree of correlation between the genome size, the proportion of repetitive sequence, and the number of genes (Feng et al., 2017; Hou and Lin, 2009; Nishitsuji et al., 2020; Wang et al., 2021). These observations support the idea that larger genomes generally having more gene count, fewer repeats and lower GC content. While, it seemed larger genomes exhibited more gene count at the genus level of Sphaeropleales, the relationship the genomes size among the GC and repeat content showed no obvious pattern, more high-quality genomes will contribute to explore the relationships.

### 3.3 Analysis of orthologous gene families and comparative analysis of predicted gene function

**Figure 2:**
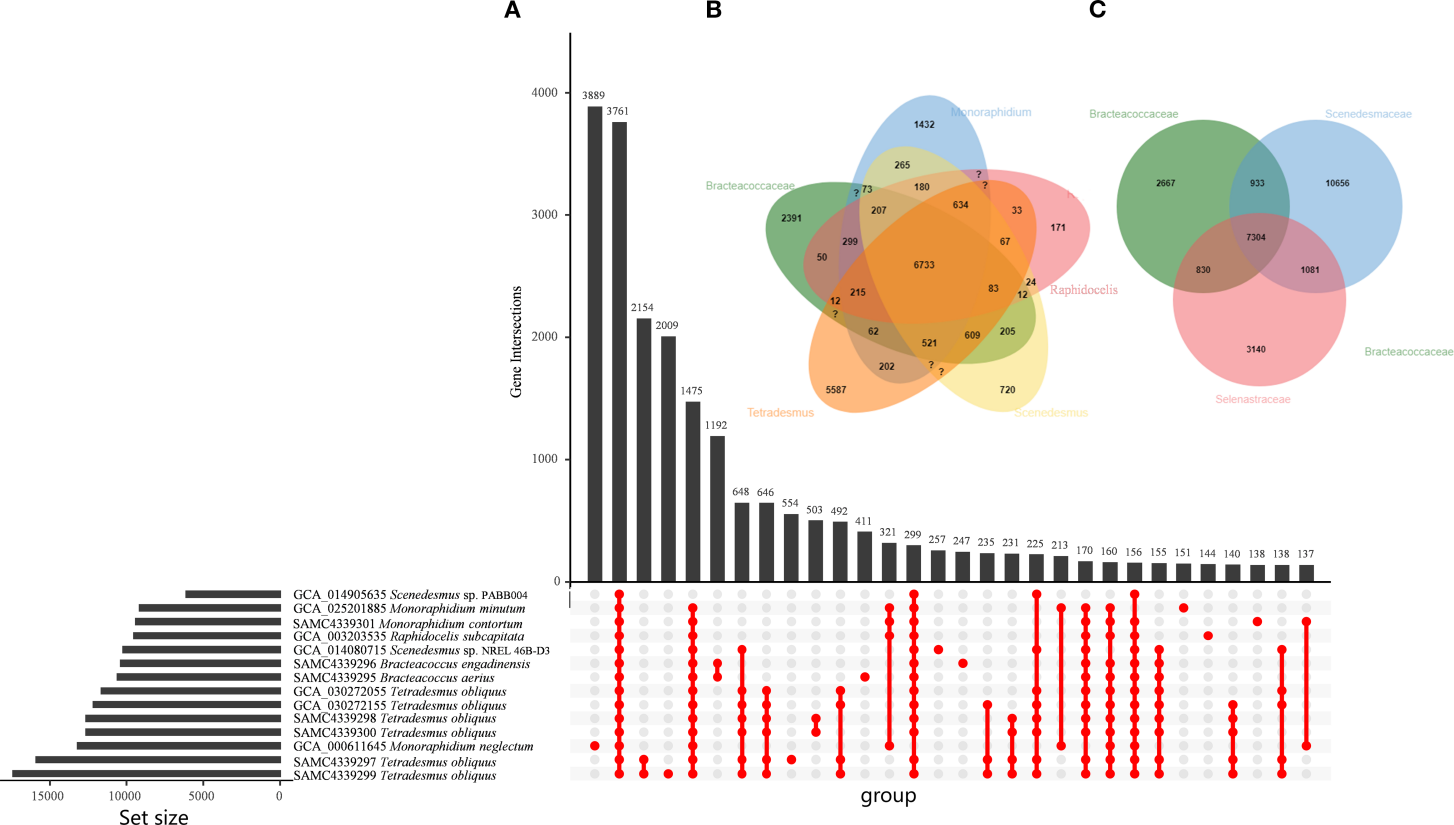
Orthologous gene analysis in genomes of the Sphaeropleales algae. **(A)** Shared families are shown on the horizontal axis and the number of patterns in the vertical axis. **(B)** Venn diagram showing the numbers of gene families shared among the Sphaeropleales algae at the level of genus. **(C)** Venn diagram showing the numbers of gene families shared among the Sphaeropleales algae at the level of family.

We conducted orthologous gene family analysis based on the newly sequenced seven Sphaeropleales strains along with seven high-quality previously sequenced genomes at different taxonomic levels (**Figure 2**). The orthologous analysis revealed that 3,761 gene families were shared or conserved across all fifteen genomes (**Figure 2A**). Additionally, each strain contained unique gene families, comprising 2-3.5% of the total gene count: 2,009 families were unique to *T. obliquus*, 554 to *S. obliquus*, 411 to *B. aerius*, 257 to *Scenedesmus* sp. NREL 46B-D3, 247 to *B. engadinensis*, 151 to *M. minutum*, 144 to *R. subcapitata*, and 138 to *M. contortum*. At the genus level (**Figure 2B**), 6,733 gene families were shared or conserved across the five genera. Furthermore, 5,587 gene families were unique to *Tetradesmus*, 2,391 to *Bracteacoccus*, 1,432 to *Monoraphidium*, and 720 to *Scenedesmus*. At the family level (**Figure 2C**), 7,304 gene families were shared or conserved among the three families. Additionally, 10,656 gene families were unique to Scenedesmaceae, 3,149 to Selenastraceae, and 2,667 to Bracteacoccaceae. In whole, the *Tetradesmus* species, in particular, had the most unique gene families (5,587), likely due to their larger genomes.

To further investigate gene functions within the Sphaeropleales, we classified all annotated proteins from 14 genomes into functional categories using the Gene Ontology (GO) database (**Supplementary Figure S2**). The predicted proteins were categorized into three primary GO domains: molecular function (MF), cellular component (CC), and biological process (BP). We compared the contents of each category and their corresponding percentages. Among the 11 strains, unique GO domains were identified, with *T. obliquus* FACHB-276 exhibiting the highest diversity, containing 278 distinct domains across the three GO categories. For the top ten functional categories at the CC and MF levels, most species shared nearly identical terms. We also calculated the PFAM domain categories and their percentages (**Supplementary Figure S3**) and KEGG pathway categories (**Supplementary Figure S4**) for all species, which showed that all the species shared similar top-ten categories and associated percentages.

### 3.4 Comparative analysis of predicted gene function

Based on the Transporter Classification Database, we analyzed the transporters in the Sphaeropleales genomes. A total of 320 transporters were identified, as shown in **Supplementary Table S1**. The transporters across the Sphaeropleales species exhibited similar patterns in terms of the top ten transporter categories and their respective numbers. As additional species were included in the analysis, the results were consistent with previous studies (Suzuki et al., 2018), which showed that Sphaeropleales species possess notably higher numbers of genes encoding for H+/hexose transporters (2.A.1.1, TCAD ID), amino acid permeases (2.A.18.2), peptide transporters (2.A.17.3), aquaporins (1.A.8.8), and metal-nicotianamine transporters (2.A.67.2) compared to *Chlamydomonas reinhardtii* (**Table 2**). The Sphaeropleales are a dominant group of freshwater algae, well-adapted to diverse environmental conditions (Baldisserotto et al., 2012; Fawley et al., 2004). These species also exhibit high sensitivity to exogenous substances (Nagai et al., 2016). Previous studies have suggested that Sphaeropleales have the ability to adapt to various environmental conditions for possessing a significantly greater number of genes related to H+/hexose transporters, amino acid permeases, peptide transporters, aquaporins, and metal-nicotianamine transporters comparing to *C. reinhardtii* (Suzuki et al., 2018). Aquaporins help algae adapt to high salt stress by facilitating the transport of small polar molecules, such as water, across cell membranes, thereby regulating intracellular osmotic pressure (Anderberg et al., 2011). Additionally, the presence of multiple metal-nicotianamine transporters, ABC transporters, and genes involved in heavy metal ion and xenobiotic transport (**Supplementary Table S2**) suggested that Sphaeropleales may have a high sensitivity to metals, positioning them as potential phytoremediation organisms for removing heavy metal pollution from aquatic environments (Benderliev and Ivanova, 1994, 1996; Murata et al., 2006; Schaaf et al., 2004). Genes related to H+/hexose cotransport, amino acid/peptide transporters, and nitrate/nitrite transporters are likely key to their rapid growth under varying nutrient conditions (Cho et al., 1981; Sauer et al., 1983).

**Table 2:** The number of genes for several transporters.

Annotation	Aquaporin	Hexose transporter	Peptide transporter	Amino acid permease	Metal-nicotianamine transporter
TCAD ID	1.A.8.8	2.A.1.1	2.A.17.3	2.A.18.2	2.A.67.2
*R*. *subcapitata*	0	13	5	8	6
*M*. *neglectum*	3	14	8	7	4
*T*. *obliquus*	8	12	3	11	5
*C*. *zofingiensis*	4	12	3	4	4
*C*. *reinhardtii*	1	3	1	0	0
*T. obliquus* (SAMC4339297)	4	17	3	8	6
*T. obliquus* (SAMC4339299)	8	20	2	11	5
*T. obliquus* (SAMC4339298)	6	21	3	13	7
*T. obliquus* (SAMC4339300)	2	21	1	13	9
*B. aerius* (SAMC4339295)	3	5	2	2	1
*B. engadinensis* (SAMC4339296)	1	6	1	4	1
*M. contortum* (SAMC4339301)	4	15	2	8	5

The data of R. subcapitata, M. neglectum, T. obliquus, Chromochloris zofingiensis and C. reinhardtii was cited from the study of Suzuki et al., 2018.

Furthermore, Sphaeropleales species possessed a gene encoding pyrroline-5-carboxylate reductase, which synthesizes proline to alleviate osmotic stress under cold conditions (Liu et al., 2020). And pyrroline-5-carboxylate reductase is also associated with halotolerance (Arora et al., 2019; Kishor et al., 1995; Pérez-Arellano et al., 2010). Previous study indicated that copies of the pyrroline-5-carboxylate reductase gene of the halotolerant microalga *Scenedesmus* sp. NREL 46B-D3 were upregulated (Calhoun et al., 2021) in the cold stress, and pyrroline 5-carboxylate reductase was upregulated under the salt stress in the cold tolerant *M. minutum* 26B-AM and *S. obliquus* UTEX393 (Calhoun et al., 2022). They also exhibited genes encoding antioxidant enzymes (e.g., catalase, ascorbate peroxidase) and antioxidant biosynthesis pathways (e.g., glutamate, β-carotene) (**Supplementary Table S3**), which helped mitigate oxidative stress from excess reactive oxygen species (O_2_ and H_2_O_2_) under cold stress (Fryer et al., 1998; Liu et al., 2020; Prasad, 1997; Van Breusegem et al., 1999). The findings from this study, based on a larger set of sequenced Sphaeropleales genomes, supported with adaptation for different environmental condition.

All Sphaeropleales species showed the presence of genes involved in vitamin B biosynthetic pathways, including the vitamin B6 biosynthetic process, thiamine (vitamin B1) biosynthesis and metabolism, biotin (vitamin B7) synthase activity, and biotin biosynthesis (**Supplementary Table S4**), indicating the ability to synthesize these vitamins *de novo*, similar to *C. reinhardtii*, *Cyanidioschyzon merolae*, and *Thalassiosira pseudonana* (Croft et al., 2006). A previous study on the marine diatom *Skeletonema costatum* has shown that a mixture of vitamin B compounds plays a crucial role in mitigating the harmful effects of hypersalinity (Gede et al., 2017). Furthermore, the possession of a complete pathway for thiamine biosynthesis contributes to enhanced biotic and stress resistance (Almutairi et al., 2021). The biosynthesis of the essential amino acid methionine can occur via both B12-dependent and B12-independent isoforms of methionine synthase (MetH and MetE, respectively) (Croft et al., 2005, 2006; Helliwell et al., 2011). In this study, all the Sphaeropleales species showed no genes about cobalamin (vitamin B12) biosynthesis, while exhibited genes about cobalamin (vitamin B12) metabolic process, and cobalamin binding (**Supplementary Table S4**), indicating that methionine synthesis occurred solely via the VB12-independent pathway, namely MetE isoform in Sphaeropleales. A previous survey of 306 species aimed to determine whether these algae require vitamin B12 (Croft et al., 2006). The results indicated that none of the Sphaeropleales species showed a requirement for cobalamin, which acts as a cofactor for enzymes involved in rearrangement-reduction reactions and methyl transfer reactions. Vitamin B12 (VB12) can only be produced by bacteria (both eubacteria and archaea) in nature, and its concentration in the natural environment is typically lower than required in culture (Croft et al., 2006). Therefore, these species acquire VB12 or its precursors through a symbiotic relationship with bacteria. Such symbiotic interactions between bacteria and algae are widespread, as many algae species are capable of acquiring vitamin B12 from their bacterial partners (Croft et al., 2006; Daisley, 1969).

All species contained genes involved in the assembly, movement, and organization of cilia (**Supplementary Table S5**), as well as genes associated with meiosis (**Supplementary Table S6**). In the Sphaeropleales cell cycle, the stage with motility flagellates/) or meiosis are either not dominant or not well understood (Trainor and Burg, 1965; Yamagishi et al., 2017). Suzuki et al. proposed that immobility may force cells to adapt to different environmental conditions aided by their numerous transporters (Suzuki et al., 2018). Another possibility that a cryptic sexual cycle or previously unobserved motile life cycle stage with flagella may exist in these organisms, or these genes are nonfunctional in Sphaeropleales. This phenomenon has been reported in *C. zofingiensi* (Blanc et al., 2010; Roth et al., 2017).

### 3.5 Gene family expansion and contraction estimation and evolution analysis

**Figure 3:**
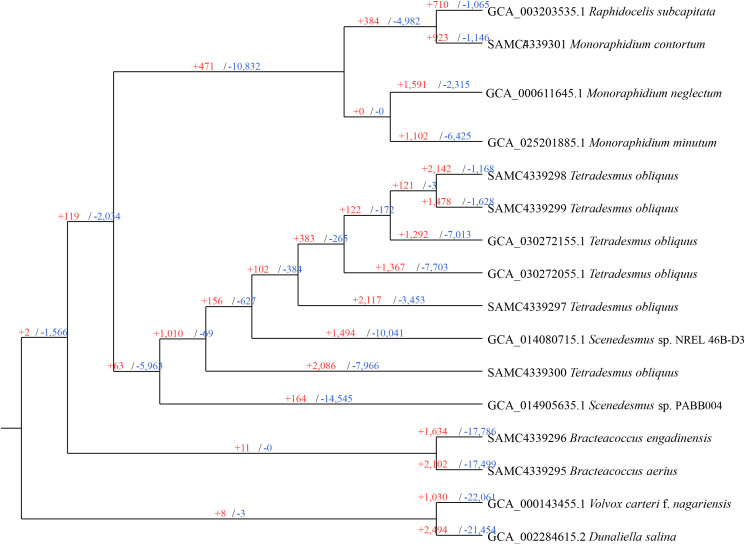
Gene family expansion or contraction in Sphaeropleales algae. Branch numbers indicate the number of gene families that have expanded (+) and contracted (−) since the split from the common ancestor.

To gain a comprehensive understanding of the orthogroups differences between species, particularly those with assignable functions, we performed gene family expansion and contraction analyses using CAFÉ, based on 29,073 gene families. As shown in **Figure 3**, all species and most ancestral nodes exhibited a substantial number of gene family expansions, with contractions being more prevalent. Specifically, *B. engadinensis* and *B. aerius* exhibited the highest number of contracted gene families, with 17,786 and 17,499 respectively, surpassing all other nodes, while *T. obliquus* (FACHB-276) displayed the most expanded gene families, with 2,142. In terms of ancestral nodes, the common ancestor of the Selenastraceae family had the largest number of expansions and contractions, with 10,832 contracted gene families and 471 expanded gene families. In contrast, the common ancestor of the Bracteacoccaceae family showed the smallest number, with only 11 expanded gene families and no contractions. The number of expanded or contracted gene families can be influenced by various factors, including gene duplication, *de novo* gene creation, gene loss, the functional roles of gene families, and environmental changes (Albalat and Canestro, 2016; Guo, 2013; Prachumwat and Li, 2008). Consequently, the observed differences in gene family expansions and contractions in Sphaeropleales are likely due to multiple causes. A previous study suggested that gene family contraction could contribute to genome reduction (Qiu et al., 2015). This may help explain why *Scenedesmus* sp. PABB004exhibited the largest number of gene family contractions, while simultaneously having the smallest genome size within the Scenedesmaceae, and the *Bracteacoccus* species showed relatively small genome size and almost the fewest gene count.

**Figure 4:**
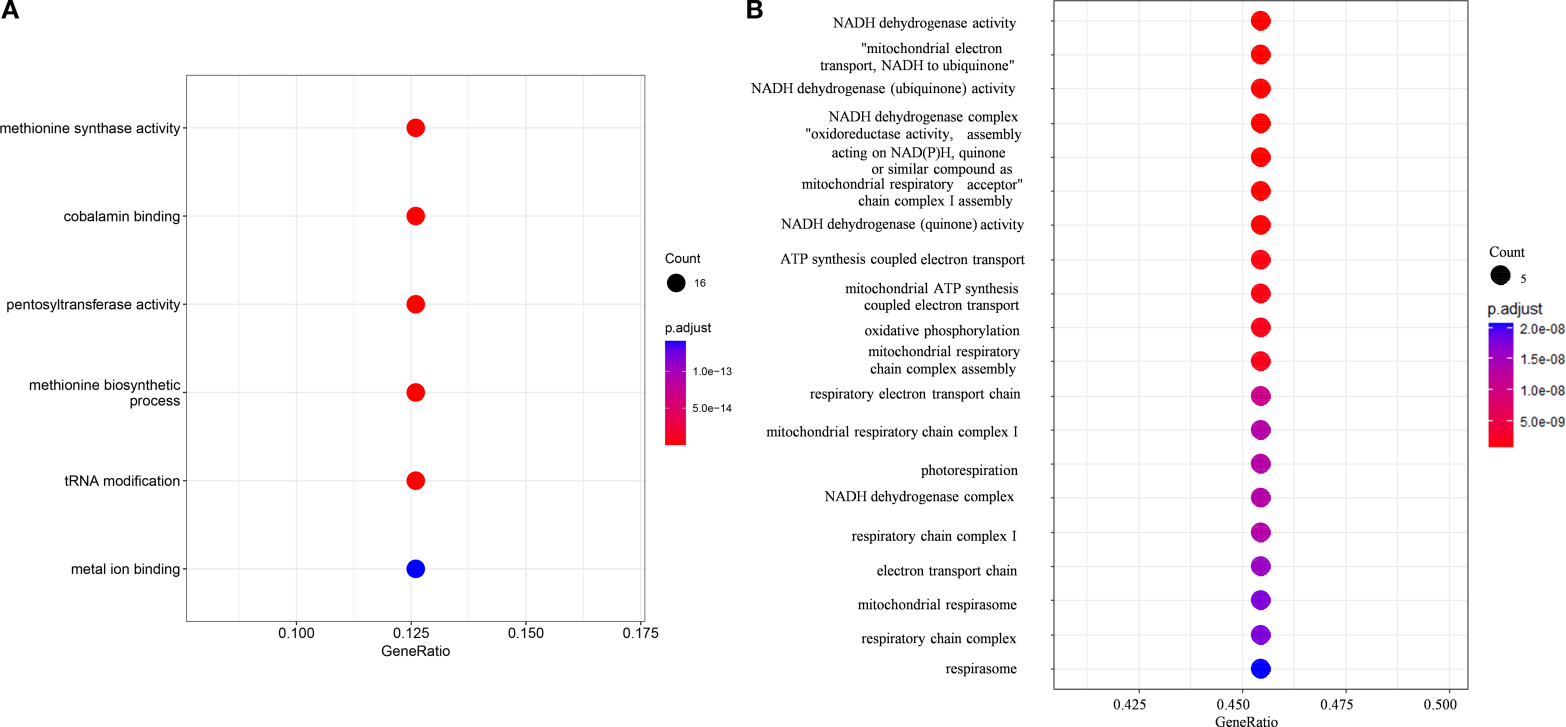
Dot plot showing the enrichment of the special orthologues about evolution. The dot sizes represent the numbers of genes, circle colors indicate different *p* values. **(A)** Dot plot showing the enrichment of the expanded orthologues in the common ancestor of Bracteacoccaceae. **(B)** Dot plot showing the enrichment of the orthologues experienced fast-evolving and positively selected.

To investigate whether gene family evolution correlates with habitat adaptation, we performed Gene Ontology (GO) enrichment analysis based on the expansions and contractions in the common ancestor of Bracteacoccaceae. The GO enrichment analysis revealed that the expanded families were enriched in six GO terms (**Figure 4A**), primarily related to methionine biosynthetic, cobalamin binding, tRNA Modification, pentosyltransferase activity, and metal ion binding.

The branch model of PAML was used to compare dN/dS ratios between terrestrial and aquatic Sphaeropleales species based on 3757 common orthologues. Among these, 3032 orthogroups showed significantly higher dN/dS ratios in the two terrestrial Bracteacoccaceae species, indicating the occurrence of rapid evolution (**Supplementary Table S7**). Positive selection analysis was performed based on branch-site model, and the null and alternative models were compared. The null model considered that the foreground branch only has dN/dS = 1, and the alternative model assumed that sites on the foreground branch have dN/dS > 1 (positive selection). When the two terrestrial Bracteacoccaceae species were labelled as the foreground branch, 13 orthogroups showed the FDR-adjusted *P* value less than 0.05, which indicated that these 13 orthogroups may possibly under positive selection (**Supplementary Table S8**), and they also under rapid evolution based on the result of Branch model. GO enrichment analysis showed these orthogroups were enriched in 87 GO terms (**Figure 4B**), among which 55 GO terms belonged to biological process category, 22 belonged to cellular component category and 10 belonged to molecular function category. Among all the three categories, there were some common functions such as oxidation-reduction process, the function of mitochondrial, the biosynthetic and metabolism of starch, polysaccharide and other organics (**Figure 4B**).

Bracteacoccaceae included only one genus *Bracteacoccus*, which are coccoid green algae that occurs in a wide range of soil types worldwide, spanning climates from polar to tropical and not avoiding even heavily polluted localities (Patova and Dorokhova, 2008; Ronquist et al., 2012; Stibal et al., 2006). Gene Ontology (GO) enrichment analysis, based on the expansions and contractions in the ancestral lineage of Bracteacoccaceae, revealed that the enriched GO terms were primarily associated with methionine biosynthesis, cobalamin binding, tRNA modification, pentosyltransferase activity, and metal ion binding. A previous study about *C. reinhardtii* indicated that methionine biosynthesis is an essential cellular mechanism for adaptation to thermal stress (Xie et al., 2013). Additionally, Zeng et al. identified key pathways in regulating natural variations in phenylpropanoid content, including flavone C-pentosyltransferase proteins, which are involved in UVB protection in Qingke (Tibetan hulless barley) (Zeng et al., 2020). It has also been reported that tRNA modifications is correlated with cold temperatures, drought, increased soil salinity, and developmental stages in vascular plants (Mmbando, 2024). Furthermore, the evolutionary analysis based on PAML showed the two species of *Bracteacoccus* exhibited genes experienced both rapidly evolution and positively selected. Among these, common functional categories included oxidation-reduction processes, mitochondrial functions, and the biosynthesis and metabolism of starch, polysaccharides, and other organic compounds. We considered these expanded gene families, rapidly evolution and positively selected were likely related to the adaption to the terrestrial habitat for Bracteacoccaceae.

## 4 Conclusions

The comprehensive genomic analysis of Sphaeropleales strains provides valuable insights into their evolutionary adaptations and genomic constituents. The phylogenetic analyses confirmed the distinctiveness of species and their relationships within the Chlorophyta. The genome sizes of Sphaeropleales species ranged from 39.8 to 151.9 Mb, with most having a GC content around 56%. Comparative analysis of orthologous gene families revealed conserved and unique gene families across species, with substantial expansions and contractions in all species and ancestral nodes. Functional annotation and analysis of transporter genes explained the importance of specific gene families in environmental adaptation. The gene family expansion and contraction analyses, along with positive selection studies, identified key functional categories associated with terrestrial adaptation in Bracteacoccaceae.

The work enriched the genomic data for Sphaeropleales, particularly the genus *Bracteacoccus*, enhancing our understanding of the ecological adaptations, evolutionary relationships, and biotechnological applications of this group of algae.

## Data Availability

The assembled data were deposited in the National Genomics Data Center (NGDC) databases BioProject PRJCA032465 (https://ngdc.cncb.ac.cn/bioproject/), and the accession numbers was SAMC4339295 - SAMC4339301, respectively.

## Supplementary material

Supplementary Figure 1 Phylogenetic tree of Sphaeropleales based on 18S rDNA sequences by Mrbayes. Numbers on branches represent support values of Bayesian posterior probabilities. Branch lengths are proportional to genetic distances, which are indicated by the scale bar. The species in bold indicates the newly added Sphaeropleales strains in this study.

Supplementary Figure 2 The top ten functional categories of the 14 Sphaeropleales based on Gene Ontology (GO) database. The red, green and blue indicates biological process (BP), cellular component (CC), and molecular function (MF) respectively.

Supplementary Figure 3 The top 20 PFAM domain categories of the 14 Sphaeropleales.

Supplementary Figure 4 The top 10 KEGG pathway categories of the 14 Sphaeropleales.

Supplementary Table 1 The analysis of the transporters in the Sphaeropleales genomes based on the Transporter Classification Database.

Supplementary Table 2 The genes about heavy metal ions and xenobiotics in the Sphaeropleales genomes.

Supplementary Table 3 The genes about encoding antioxidant enzymes (e.g., catalase, ascorbate peroxidase) and antioxidant biosynthesis pathways in the Sphaeropleales genomes.

Supplementary Table 4 The genes about vitamins B in the Sphaeropleales genomes.

Supplementary Table 5 The genes about cilium in the Sphaeropleales genomes.

Supplementary Table 6 The genes about meiosis in the Sphaeropleales genomes.

Supplementary Table 7 The fast-evolving genes based on the branch model of PMAL.

Supplementary Table 8 The positively-selected genes based on branch-site model of PAML.
