# Supplementary figures and images for: Comparative genomics and evolutionary analyses of Sphaeropleales
Source: Front Plant Sci. 2025 Oct 16;16:1534646. doi: 10.3389/fpls.2025.1534646 (PMC12571836; doi:10.3389/fpls.2025.1534646)

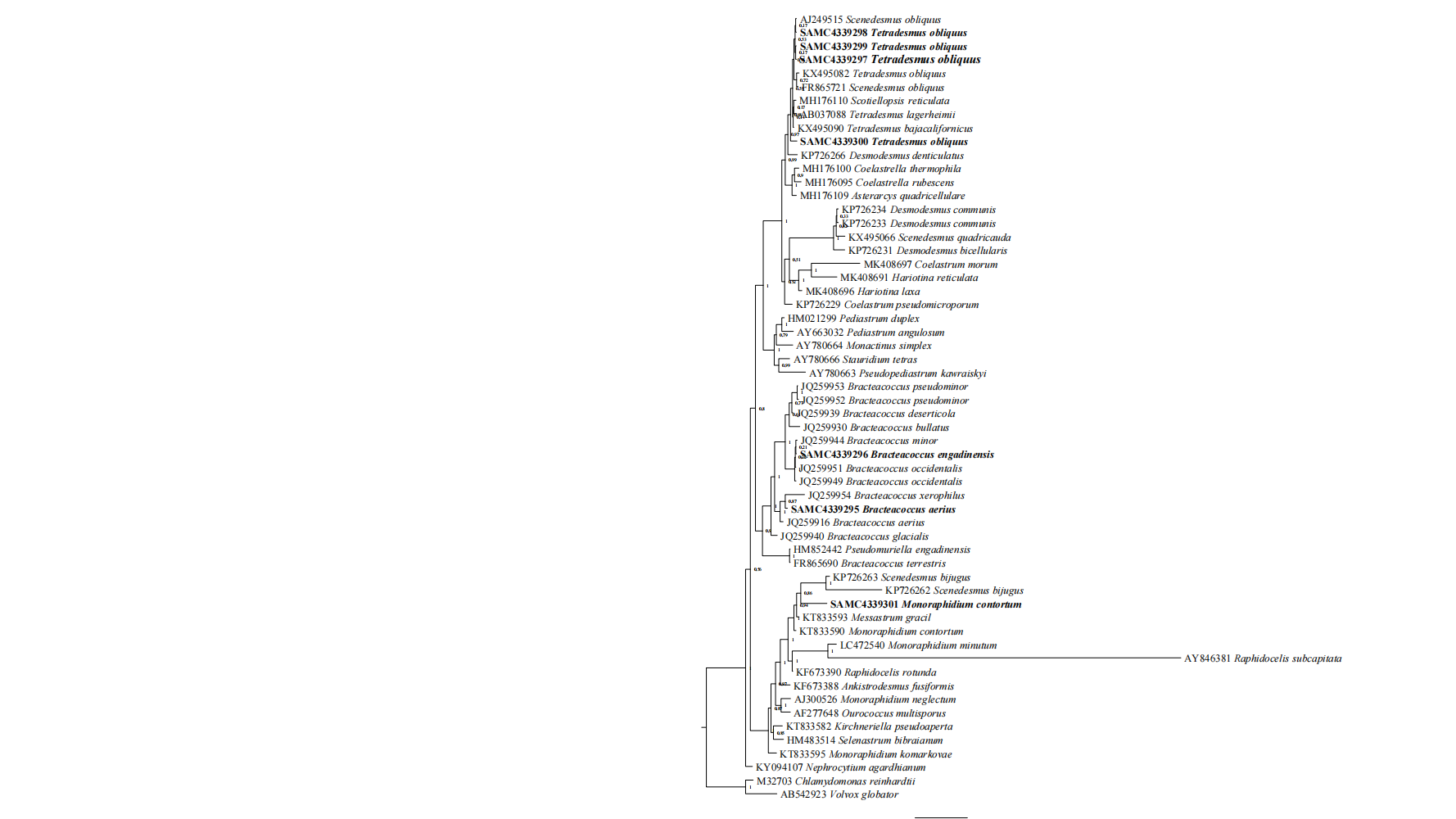

Supplement: Supplementary Figure 1 — Phylogenetic tree of Sphaeropleales based on 18S rDNA sequences by Mrbayes. Numbers on branches represent support values of Bayesian posterior probabilities. Branch lengths are proportional to genetic distances, which are indicated by the scale bar. The species in bold indicates the newly added Sphaeropleales strains in this study. [file Image1.tif]

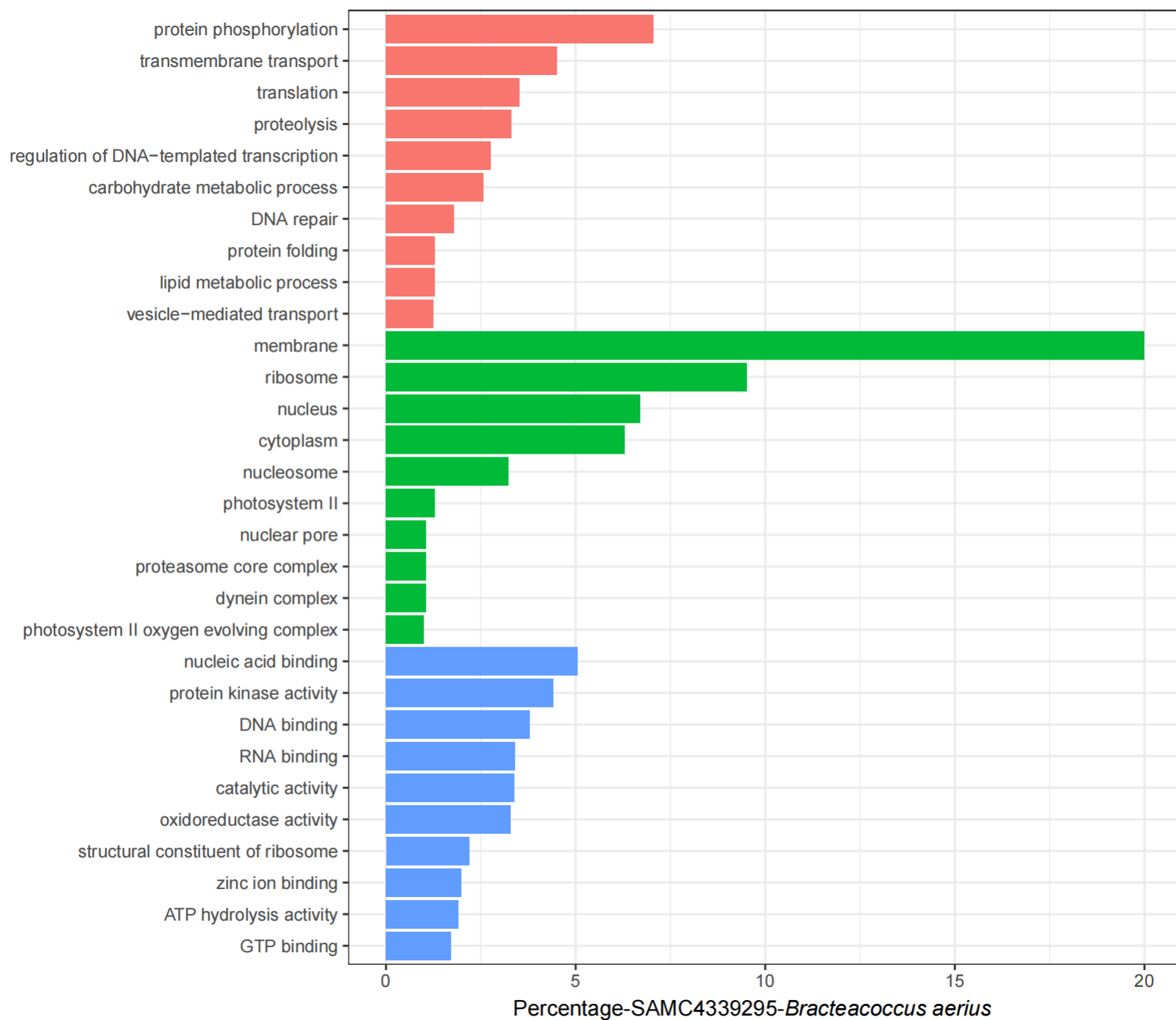

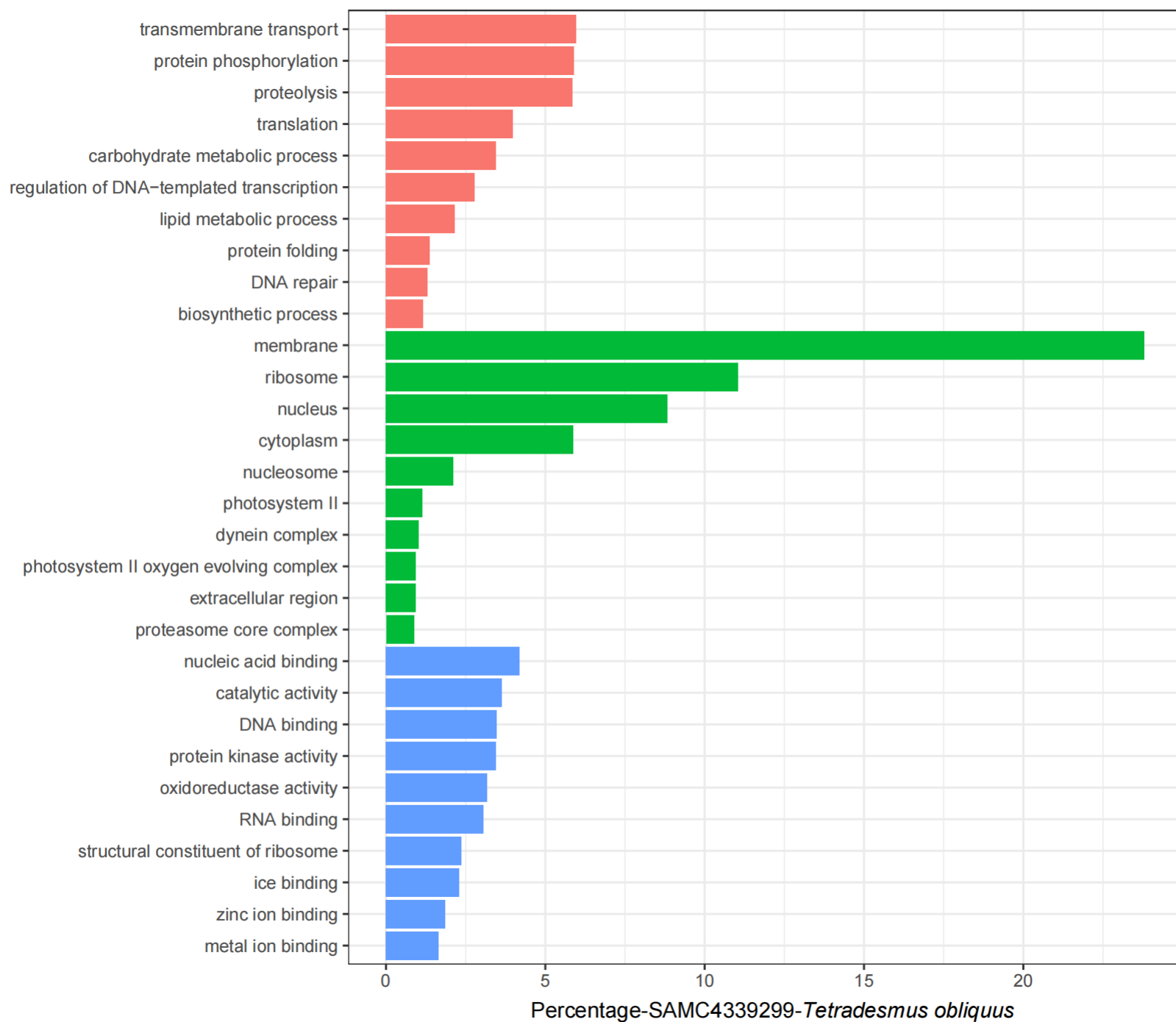

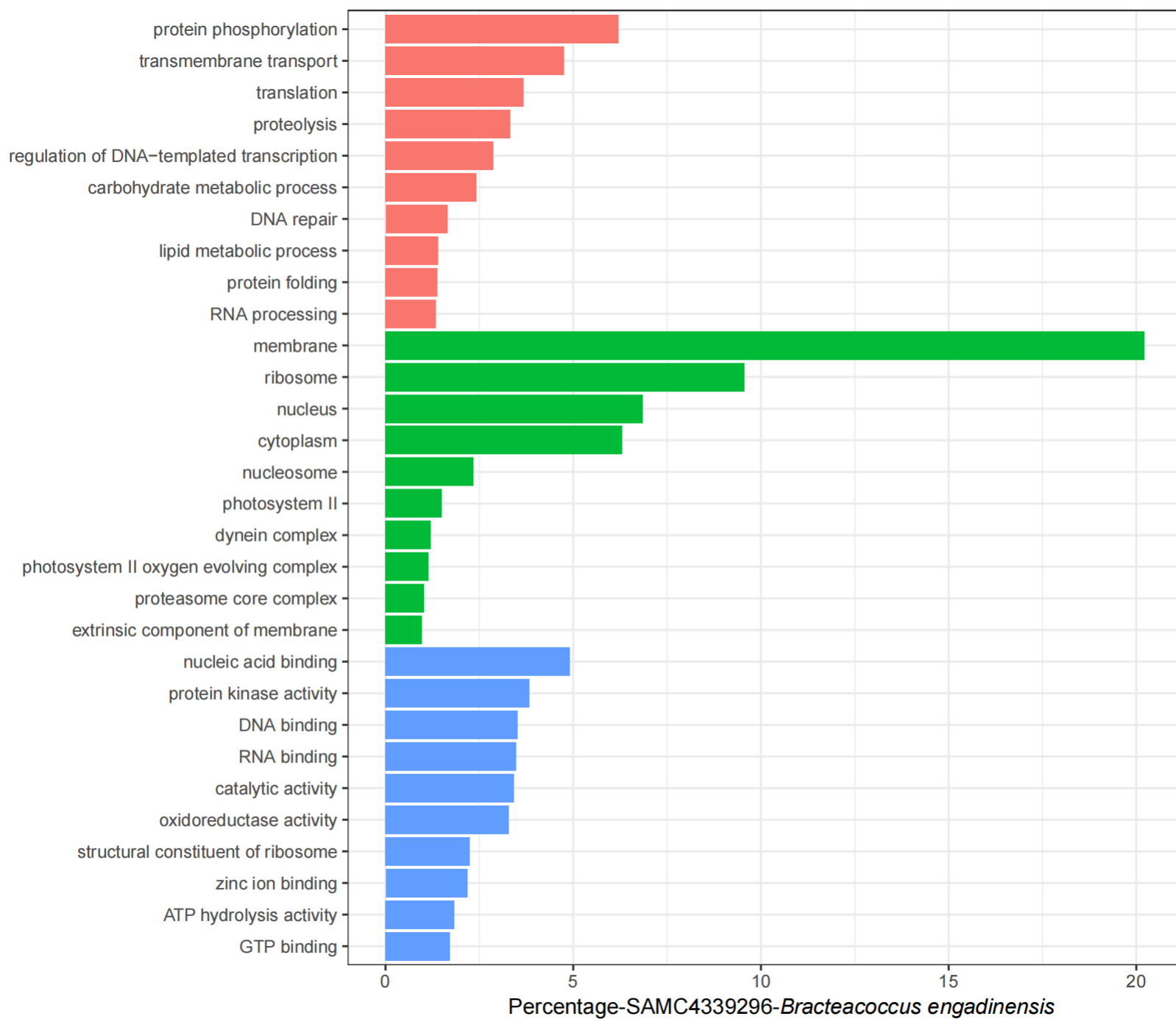

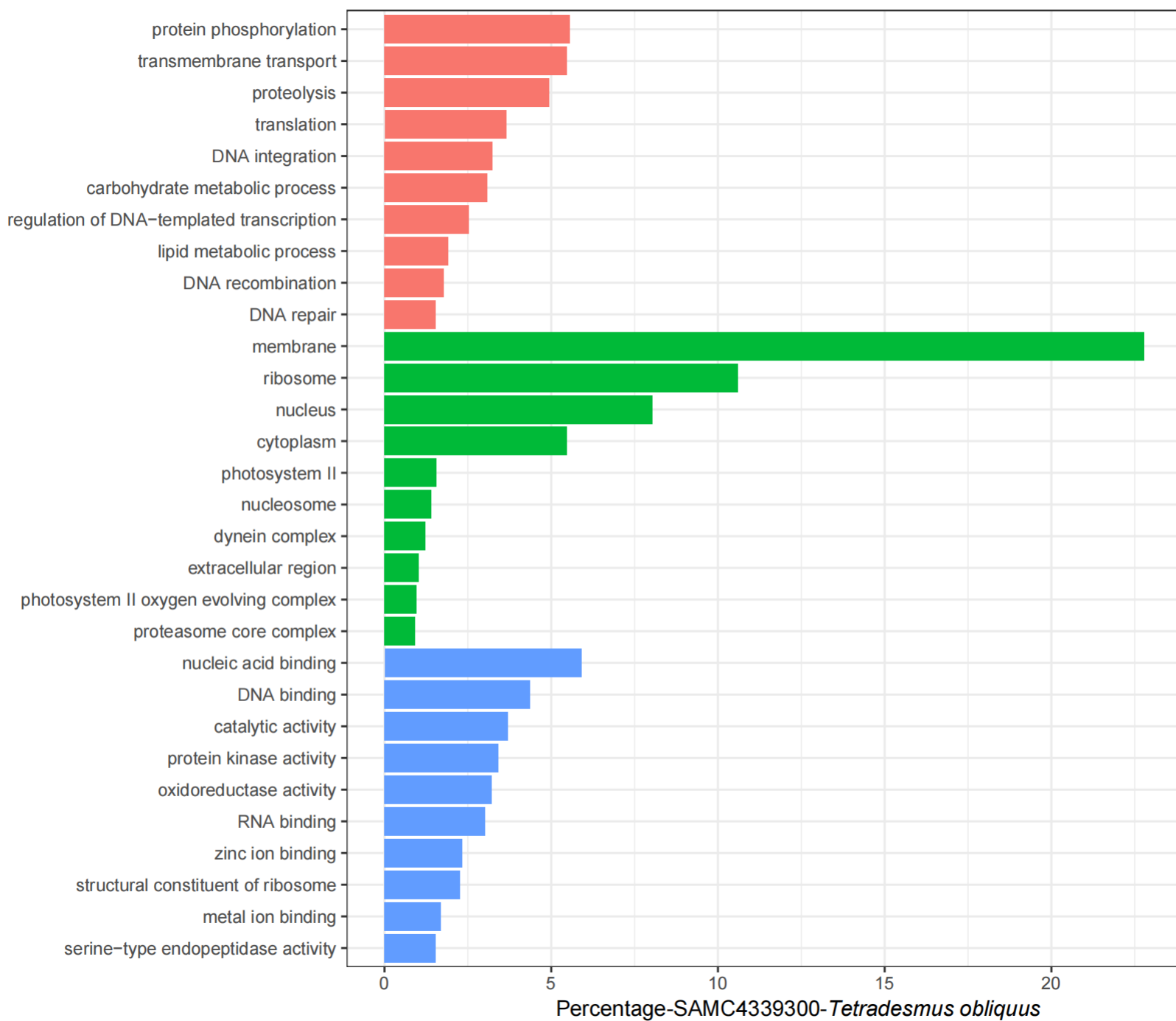

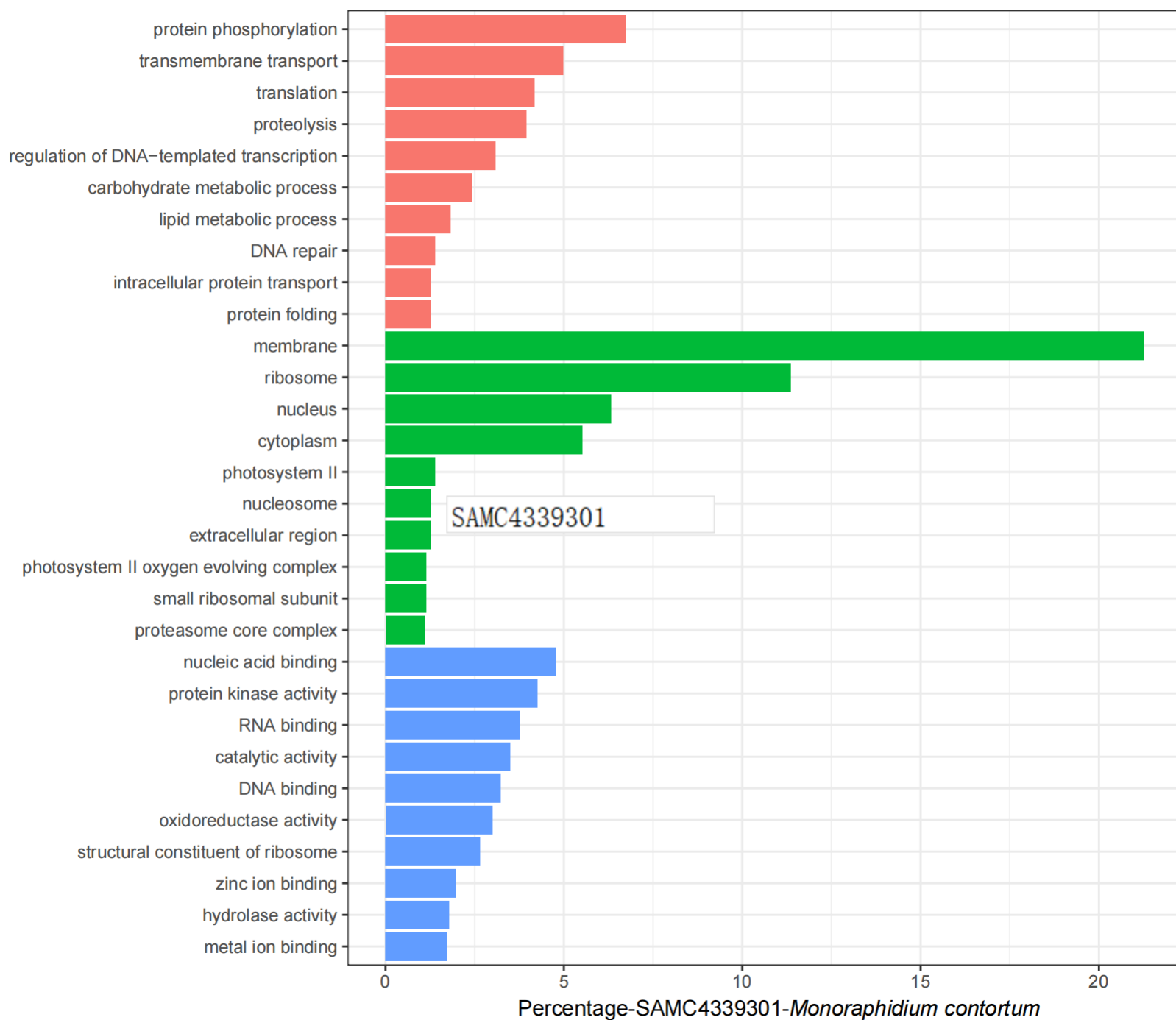

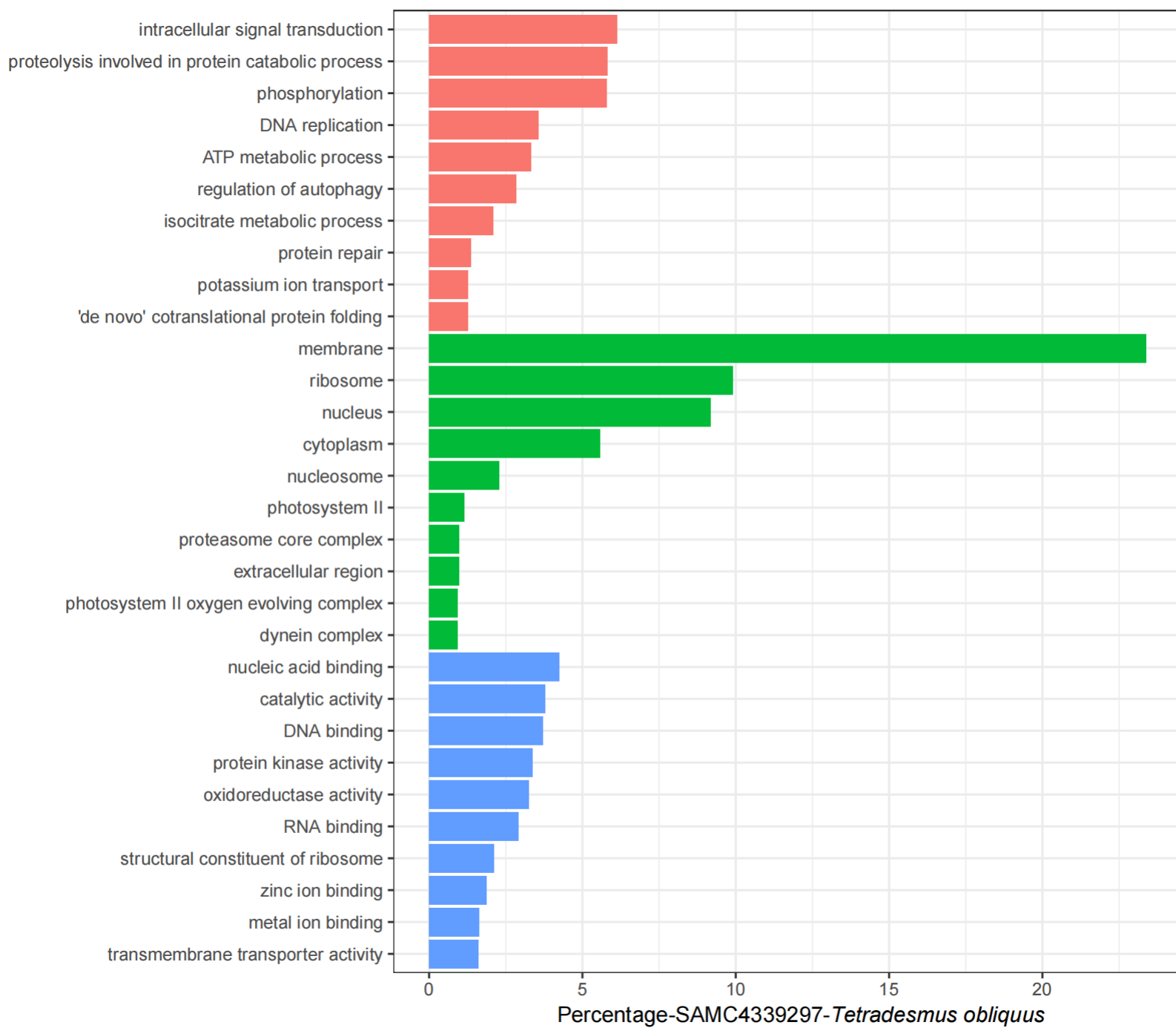

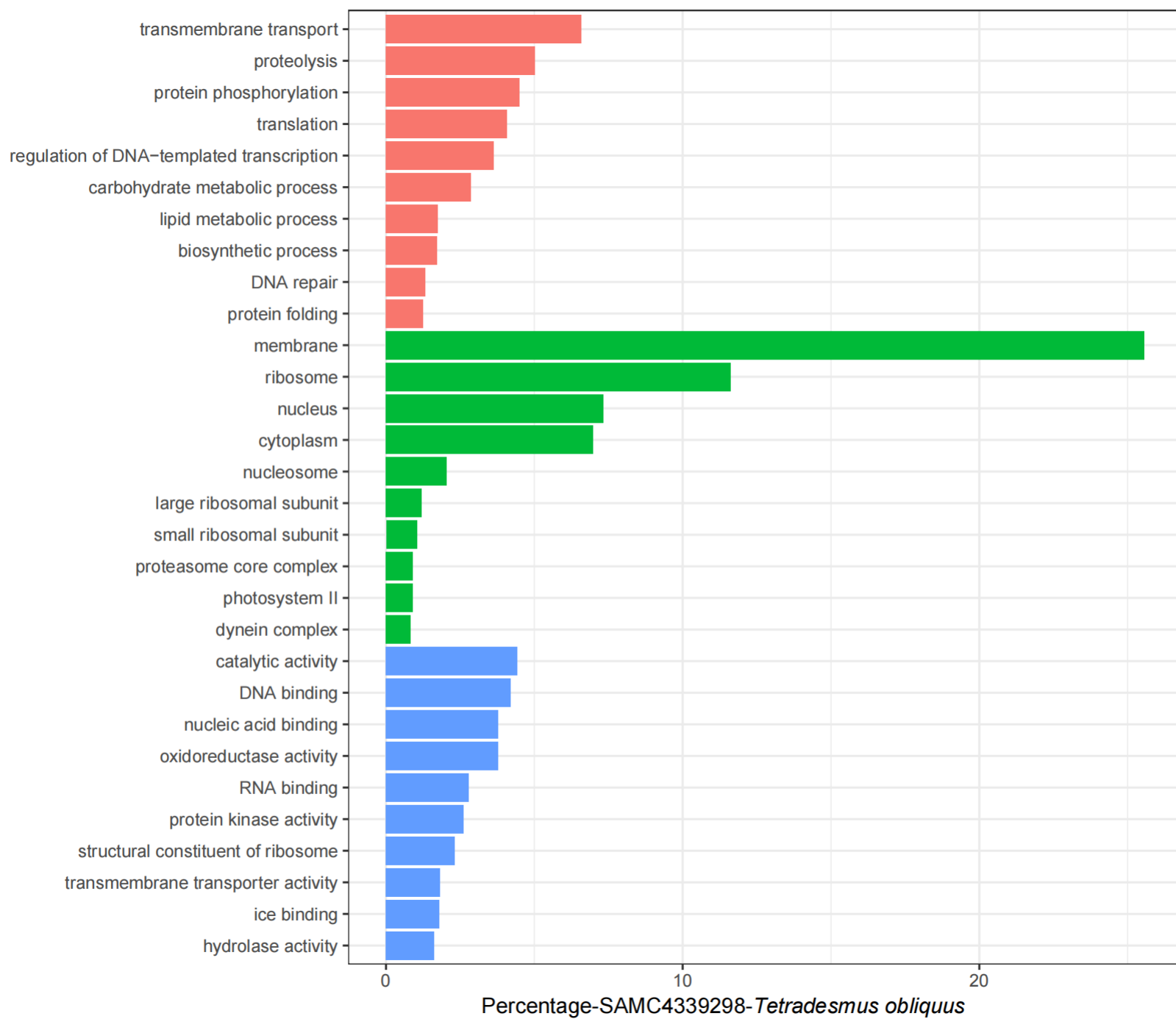

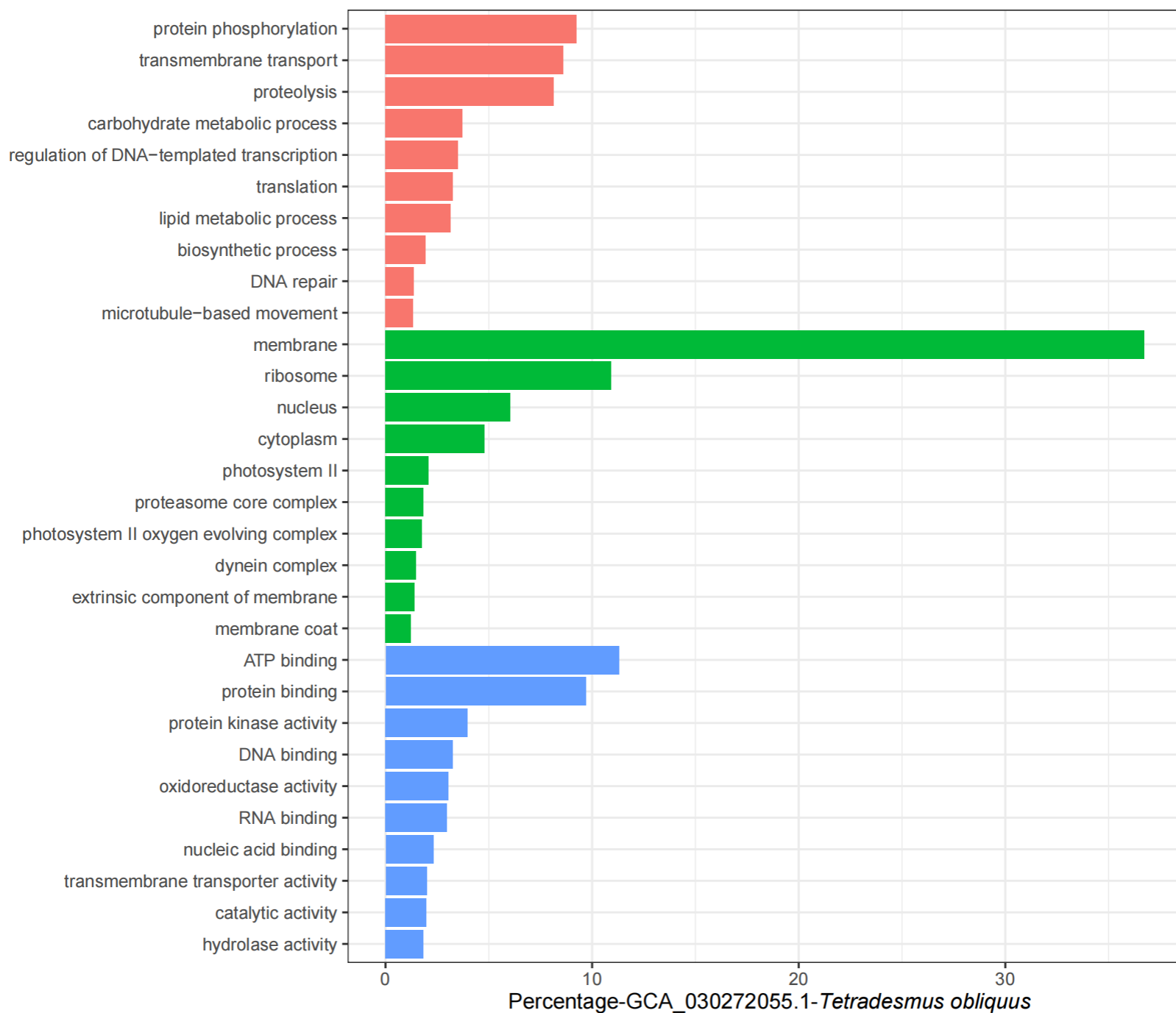

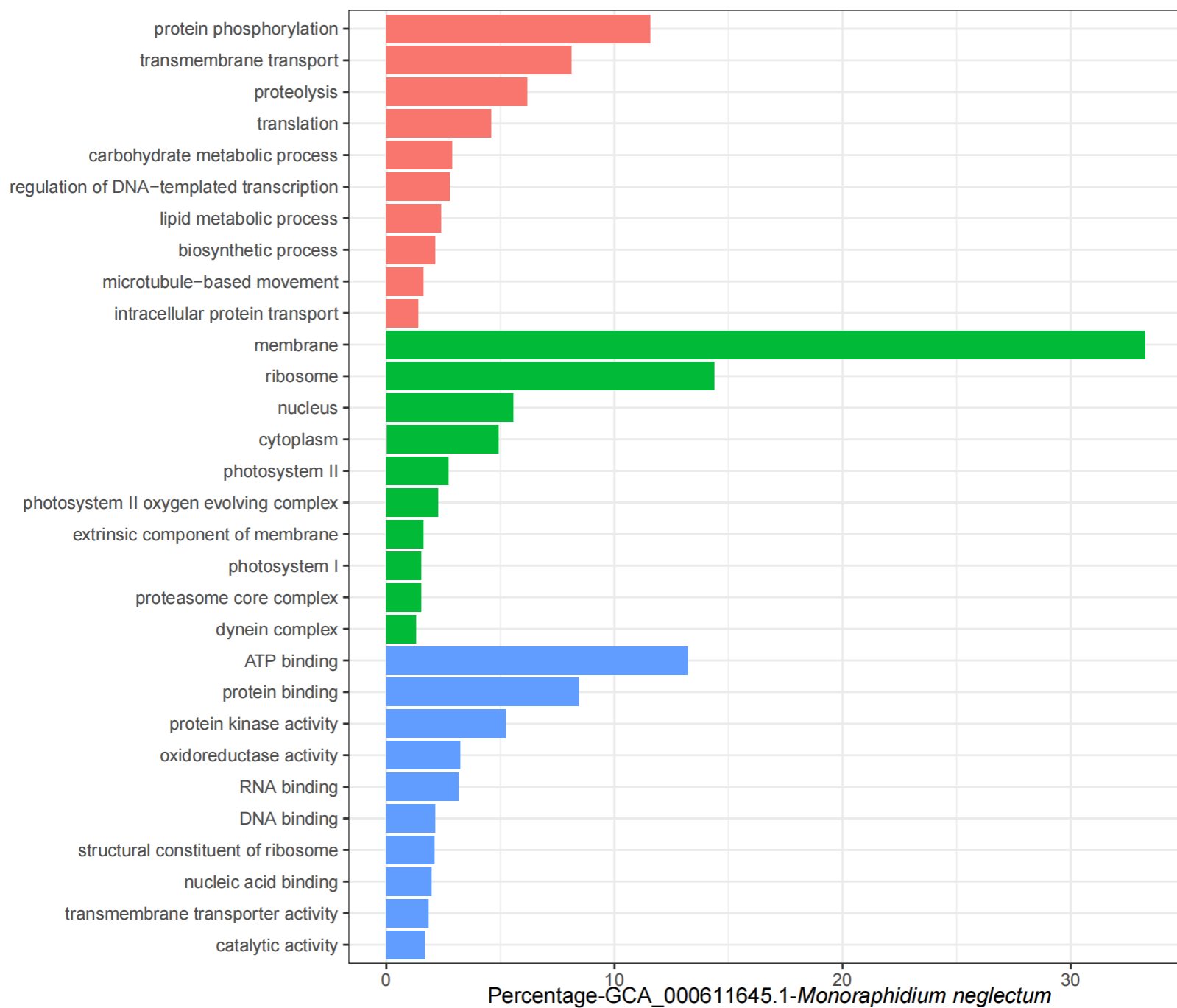

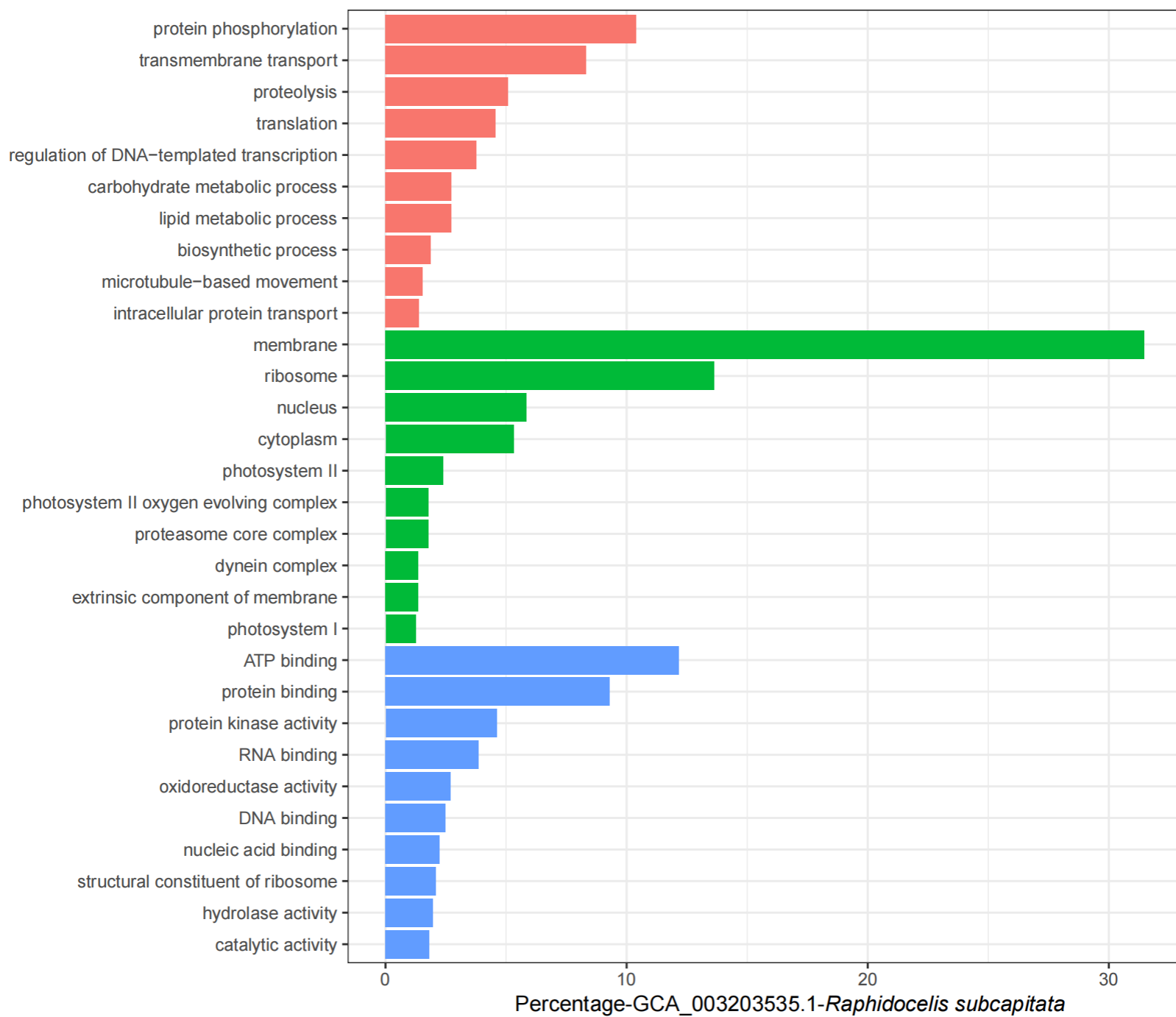

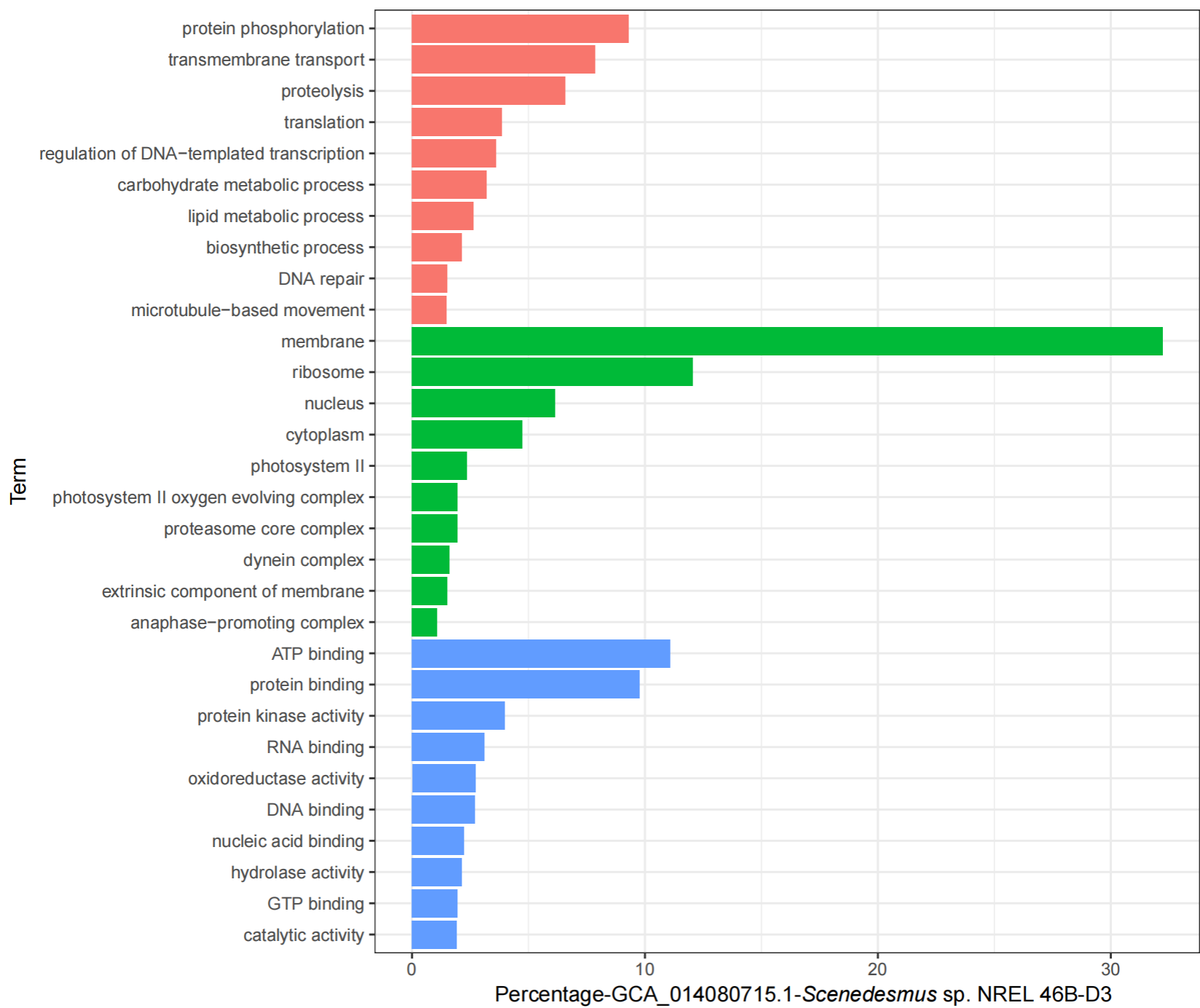

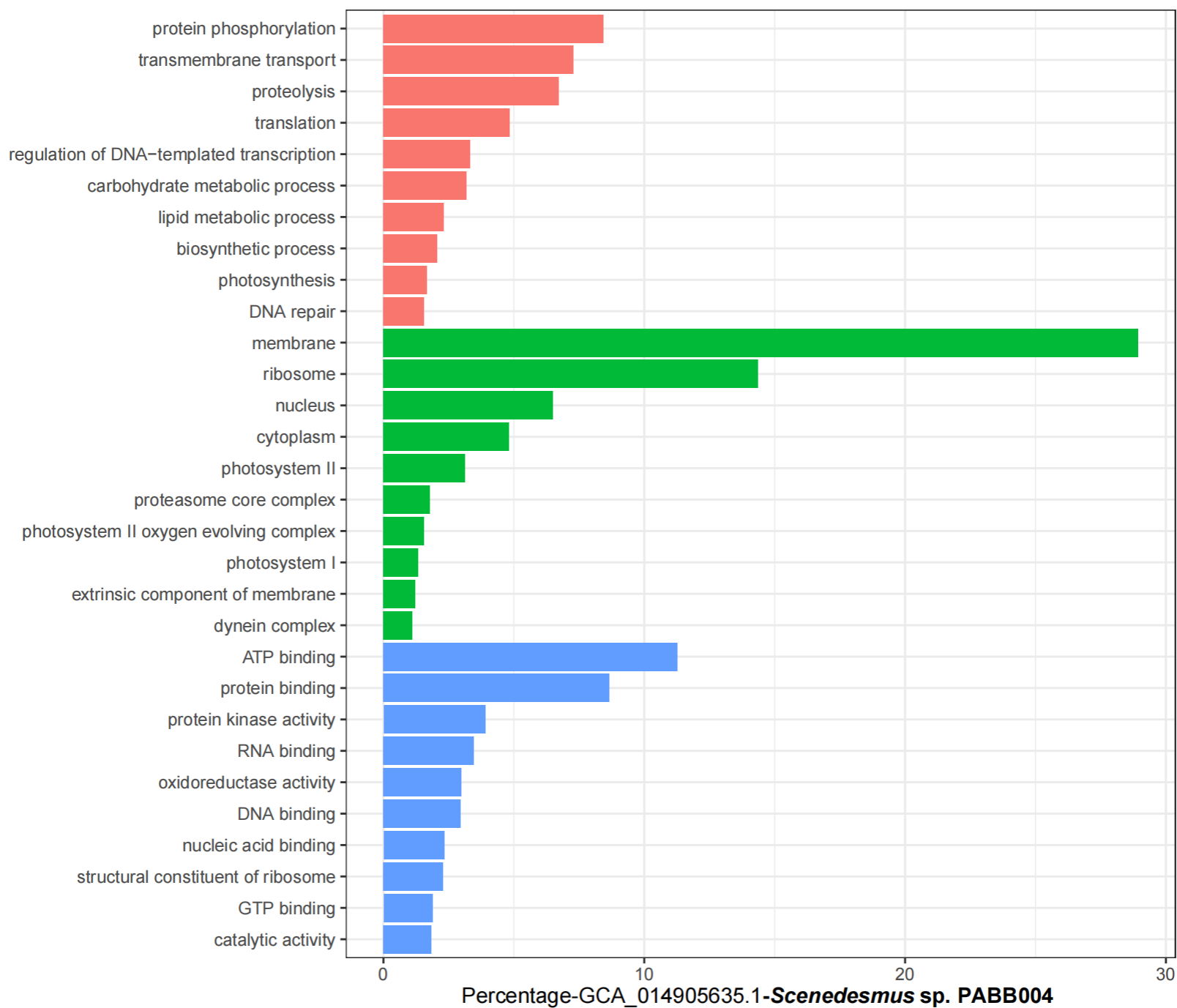

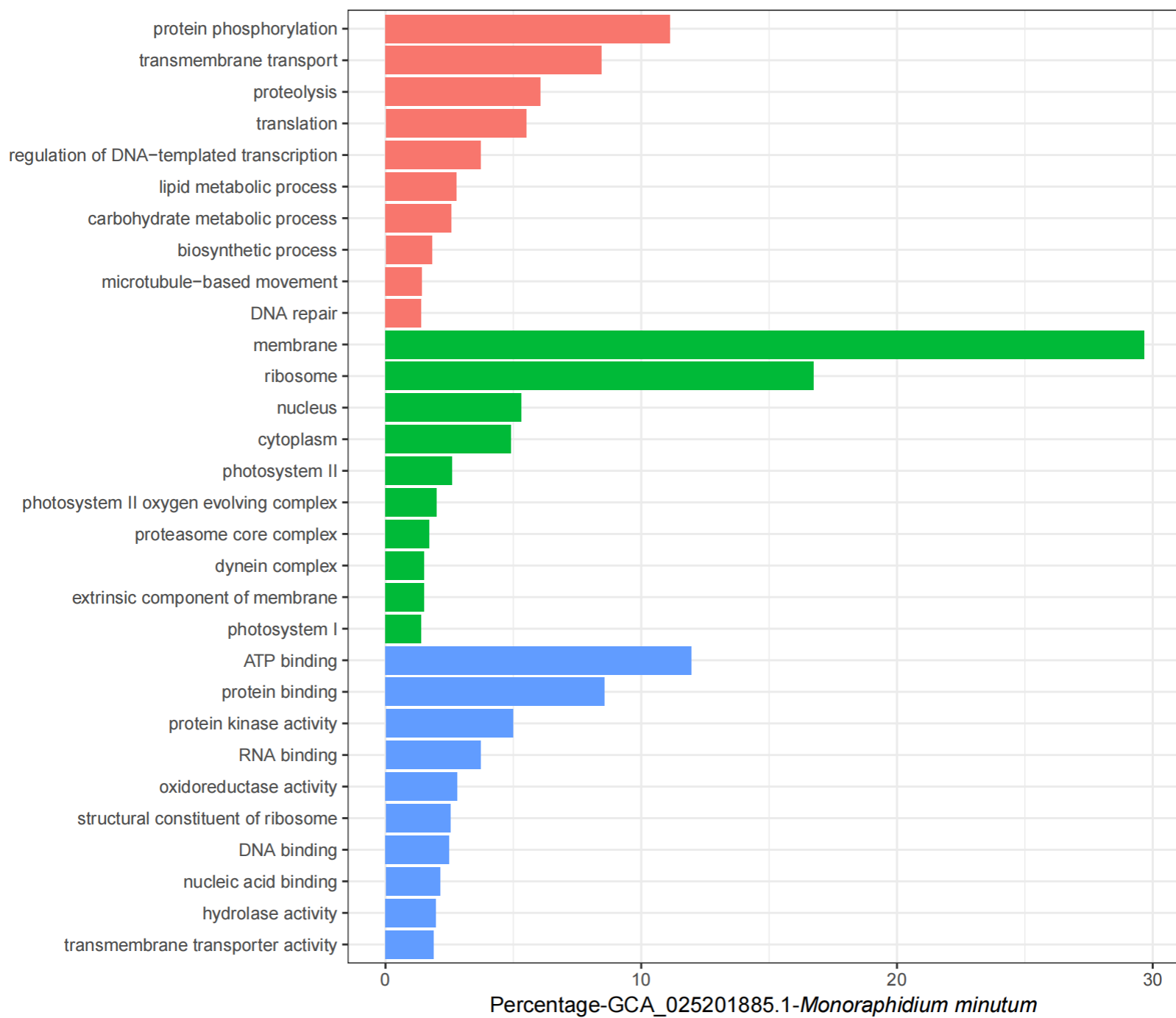

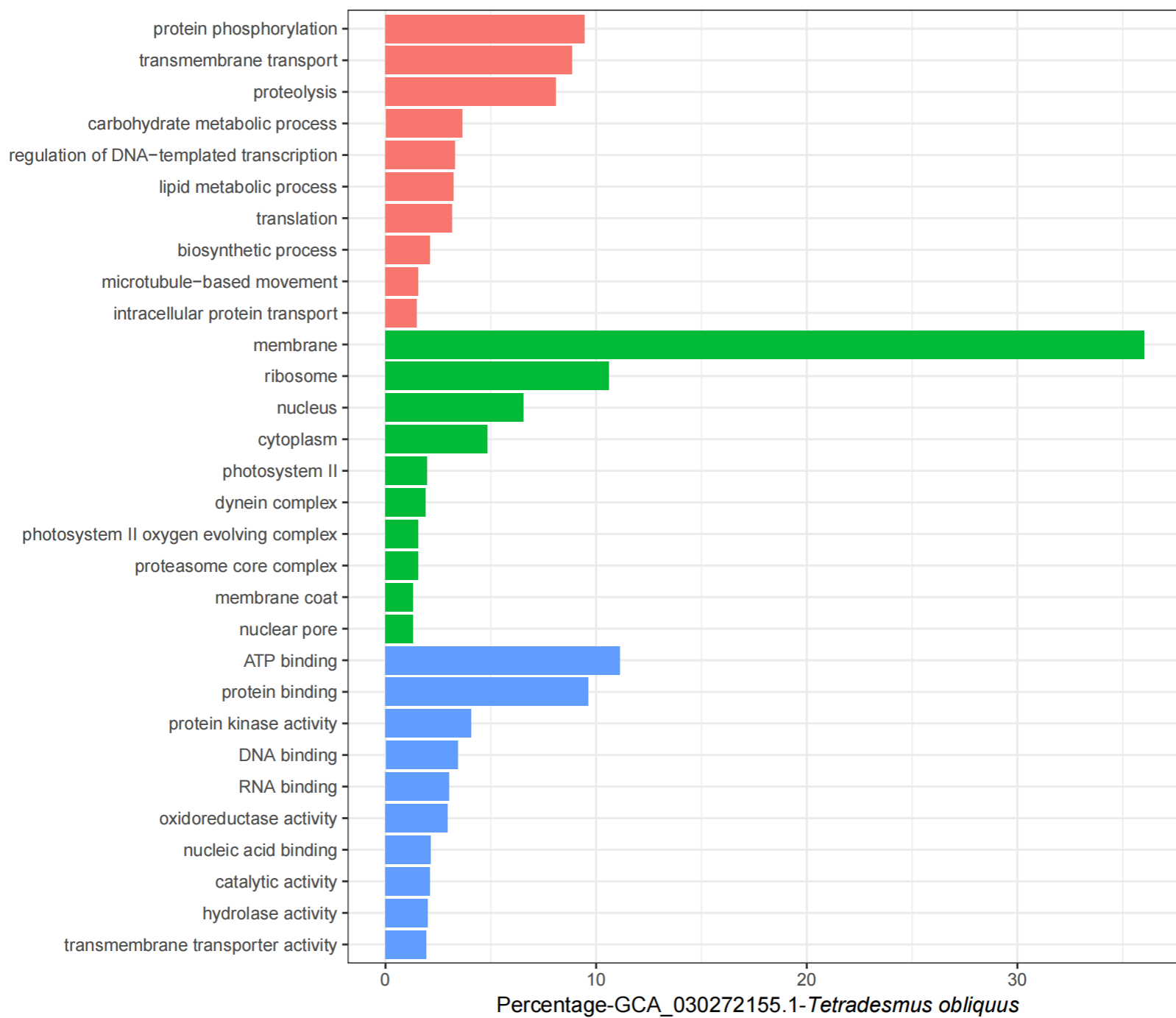

Supplement: Supplementary Figure 2 — The top ten functional categories of the 14 Sphaeropleales based on Gene Ontology (GO) database. The red, green and blue indicates biological process (BP), cellular component (CC), and molecular function (MF) respectively. [file DataSheet1.pdf]
