# Supplementary material for: Comparative genomics and evolutionary analyses of Sphaeropleales
Source: Front Plant Sci. 2025 Oct 16;16:1534646. doi: 10.3389/fpls.2025.1534646 (PMC12571836; doi:10.3389/fpls.2025.1534646)
Supplement: Supplementary Figure 3 — The top 20 PFAM domain categories of the 14 Sphaeropleales. [file DataSheet2.pdf]

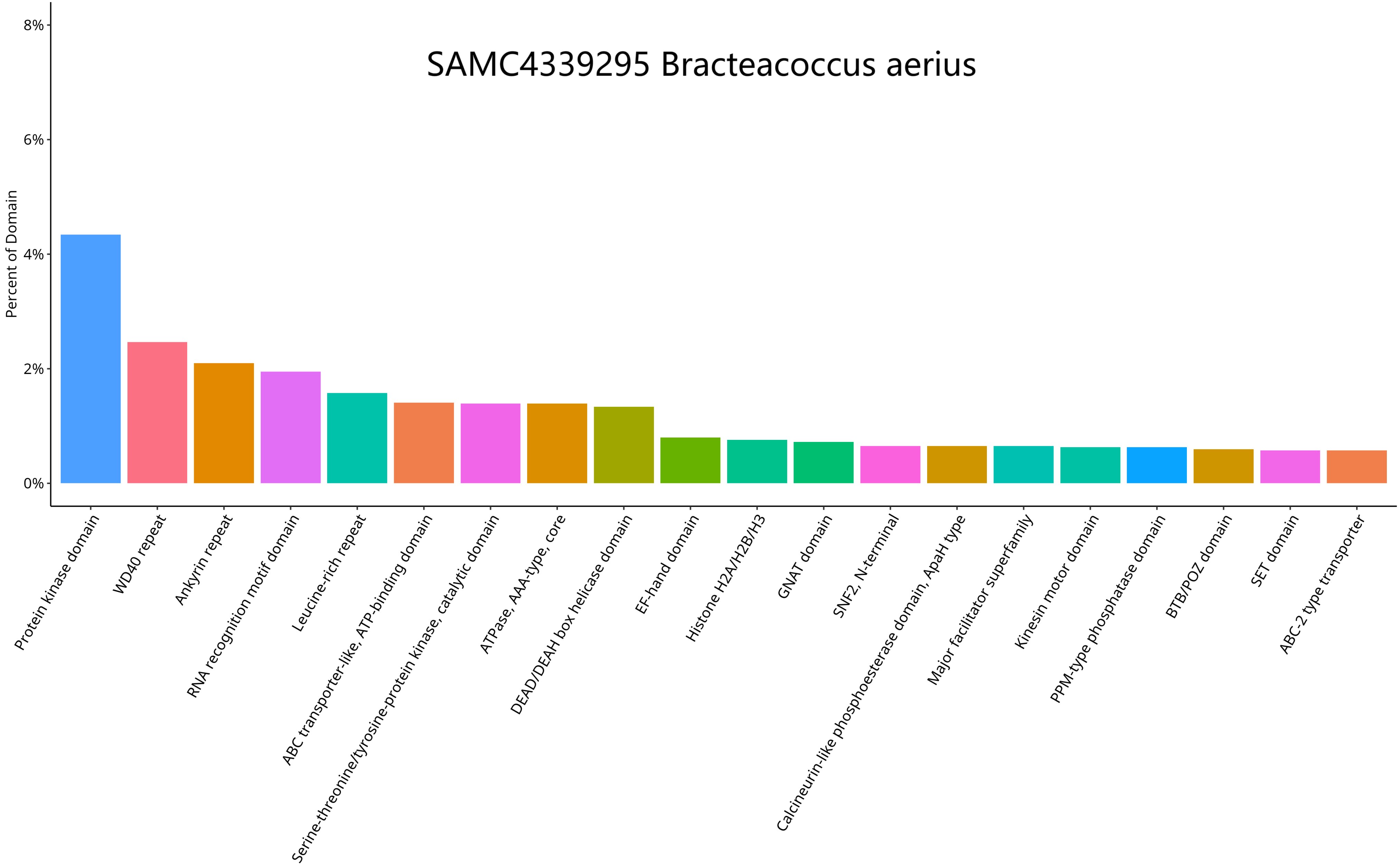
SAMC4339295 Bracteacoccus aerius
Percent of Domain
8%
6%
4%
2%
0%
Protein kinase domain
WD40 repeat
Ankyrin repeat
RNA recognition motif domain
Leucine-rich repeat
ABC transporter-like, ATP-binding domain
Serine-threonine/tyrosine-protein kinase, catalytic domain
ATPase, AAA-type, core
DEAD/DEAH box helicase domain
EF-hand domain
Histone H2A/H2B/H3
GNAT domain
SNF2, N-terminal
Calcineurin-like phosphoesterase domain, ApaH type
Major facilitator superfamily
Kinesin motor domain
PPM-type phosphatase domain
BTB/POZ domain
SET domain
ABC-2 type transporter

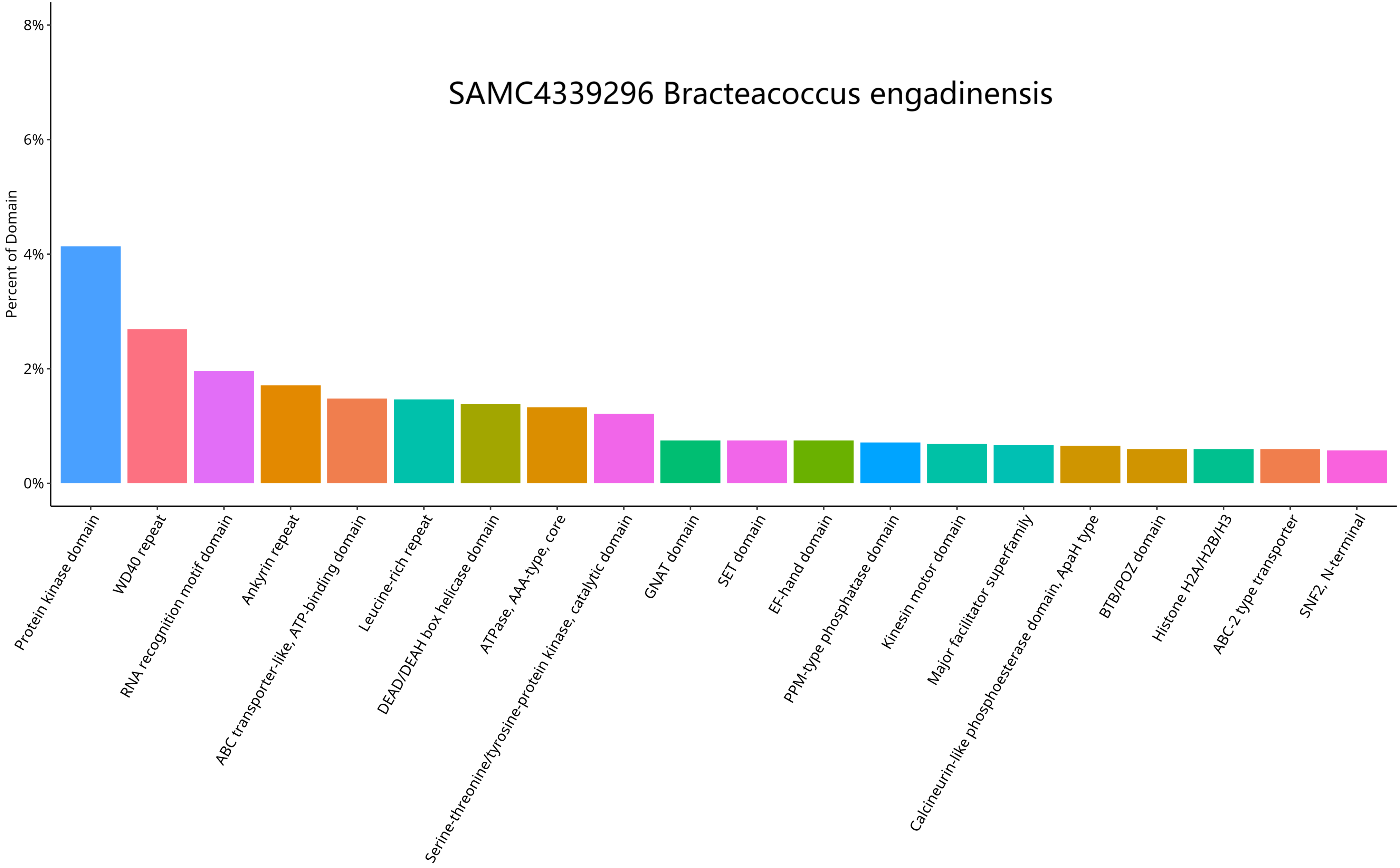
SAMC4339296 Bracteacoccus engadinensis
Percent of Domain
8%
6%
4%
2%
0%
Protein kinase domain
WD40 repeat
RNA recognition motif domain
Ankyrin repeat
ABC transporter-like, ATP-binding domain
Leucine-rich repeat
DEAD/DEAH box helicase domain
ATPase, AAA-type, core
Serine-threonine/tyrosine-protein kinase, catalytic domain
GNAT domain
SET domain
EF-hand domain
PPM-type phosphatase domain
Kinesin motor domain
Major facilitator superfamily
Calcineurin-like phosphoesterase domain, ApaH type
BTB/POZ domain
Histone H2A/H2B/H3
ABC-2 type transporter
SNF2, N-terminal

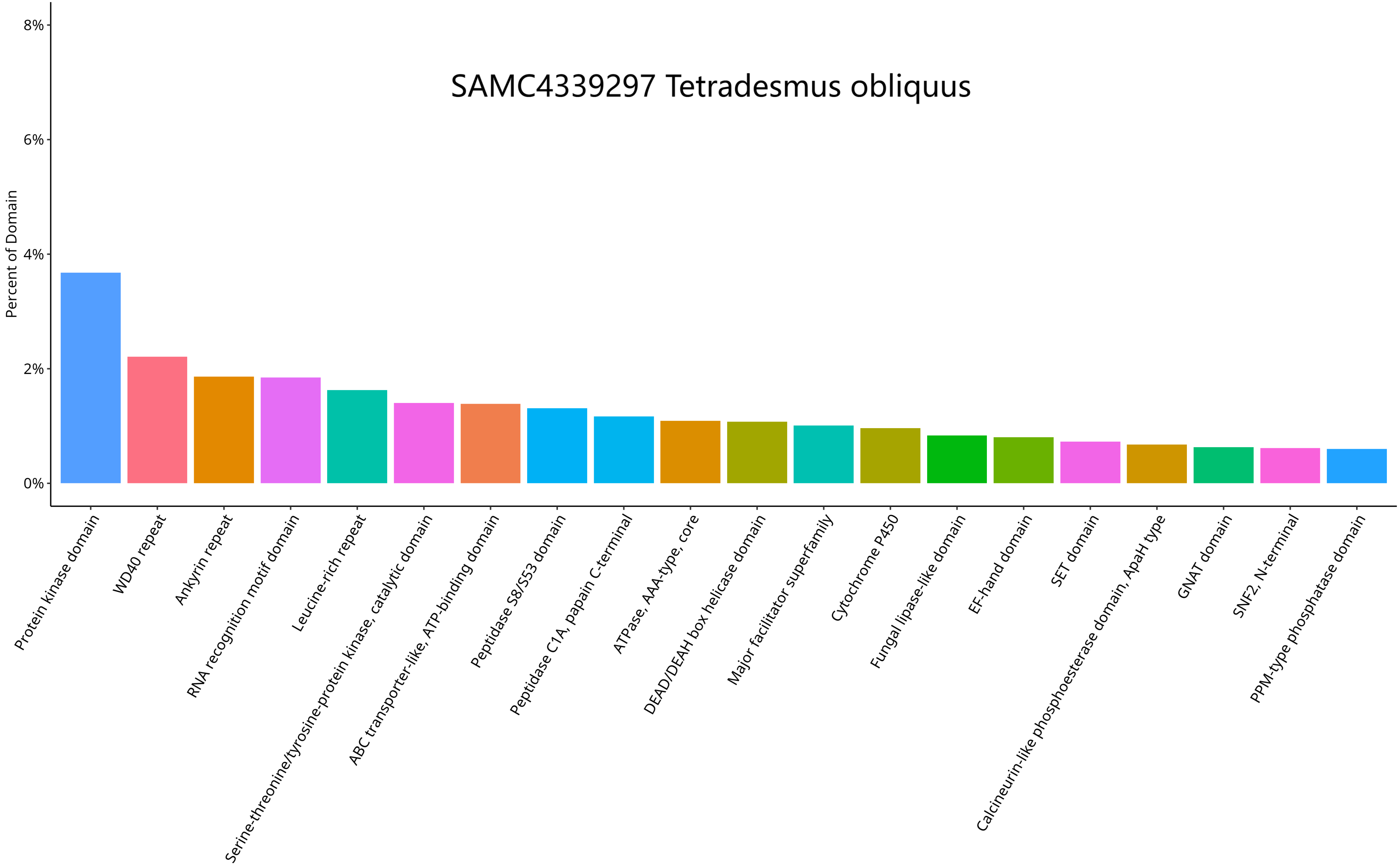
SAMC4339297 Tetradesmus obliquus
Percent of Domain
8%
6%
4%
2%
0%
Protein kinase domain
WD40 repeat
Ankyrin repeat
RNA recognition motif domain
Leucine-rich repeat
Serine-threonine/tyrosine-protein kinase, catalytic domain
ABC transporter-like, ATP-binding domain
Peptidase S8/S53 domain
Peptidase C1A, papain C-terminal
ATPase, AAA-type, core
DEAD/DEAH box helicase domain
Major facilitator superfamily
Cytochrome P450
Fungal lipase-like domain
EF-hand domain
SET domain
Calcineurin-like phosphoesterase domain, ApaH type
GNAT domain
SNF2, N-terminal
PPM-type phosphatase domain

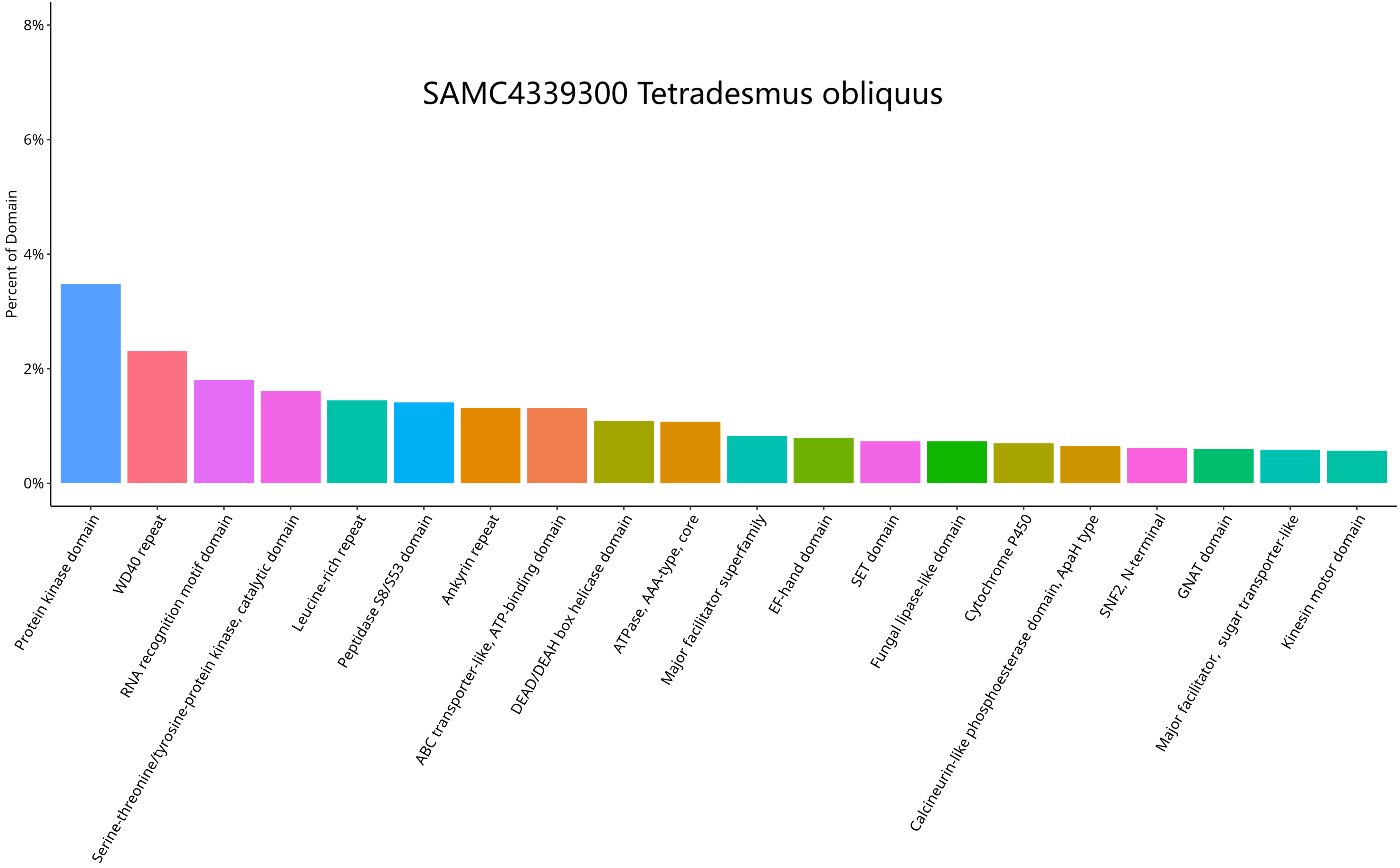
SAMC4339300 Tetradesmus obliquus
Percent of Domain
8%
6%
4%
2%
0%
Protein kinase domain
WD40 repeat
RNA recognition motif domain
Serine-threonine/tyrosine-protein kinase, catalytic domain
Leucine-rich repeat
Peptidase S8/S53 domain
Ankyrin repeat
ABC transporter-like, ATP-binding domain
DEAD/DEAH box helicase domain
ATPase, AAA-type, core
Major facilitator superfamily
EF-hand domain
SET domain
Fungal lipase-like domain
Cytochrome P450
Calcineurin-like phosphoesterase domain, ApaH type
SNF2, N-terminal
GNAT domain
Major facilitator, sugar transporter-like
Kinesin motor domain

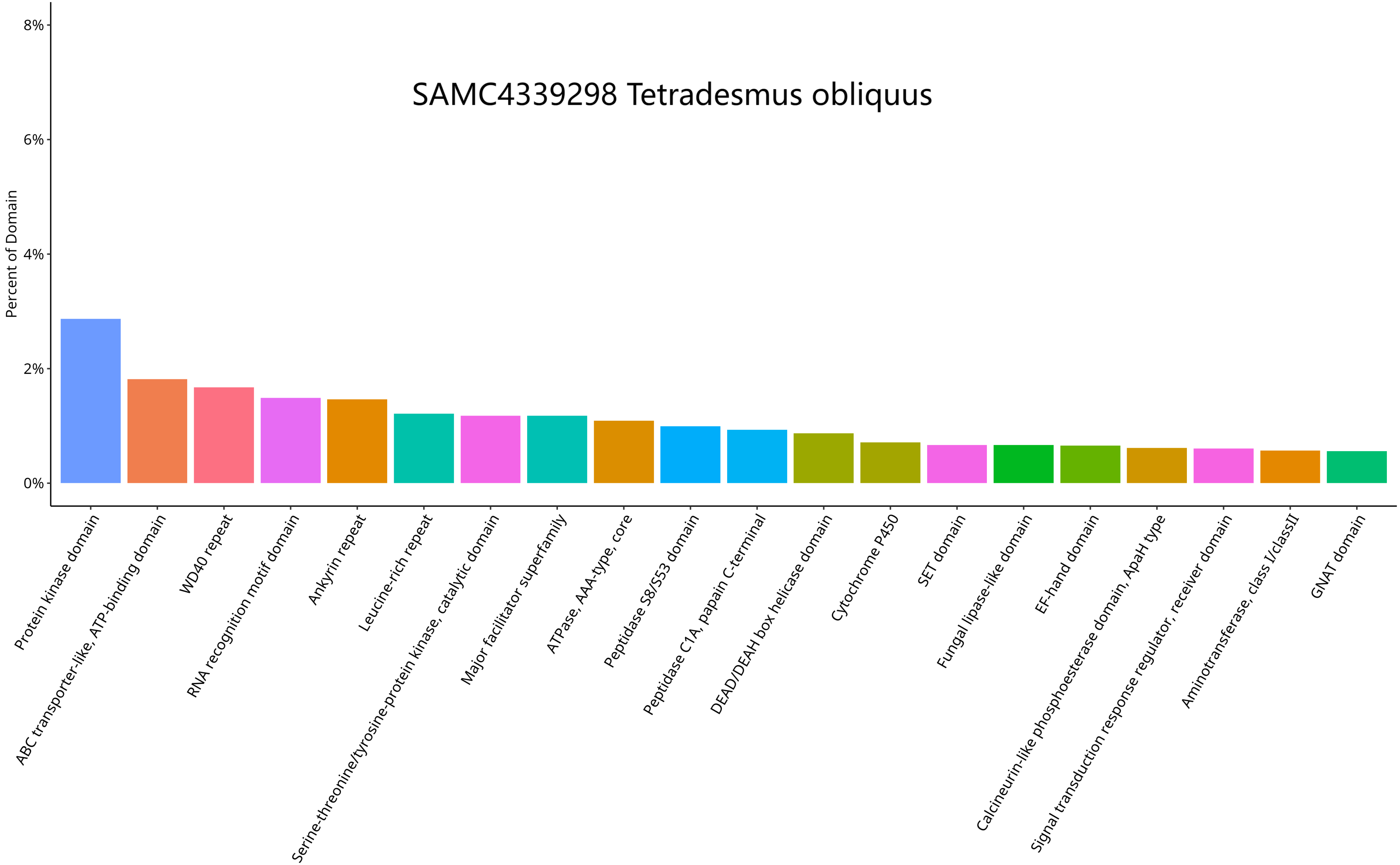
SAMC4339298 Tetradesmus obliquus
Percent of Domain
8%
6%
4%
2%
0%
Protein kinase domain
ABC transporter-like, ATP-binding domain
WD40 repeat
RNA recognition motif domain
Ankyrin repeat
Leucine-rich repeat
Serine-threonine/tyrosine-protein kinase, catalytic domain
Major facilitator superfamily
ATPase, AAA-type, core
Peptidase S8/S53 domain
Peptidase C1A, papain C-terminal
DEAD/DEAH box helicase domain
Cytochrome P450
SET domain
Fungal lipase-like domain
EF-hand domain
Calcineurin-like phosphoesterase domain, ApaH type
Signal transduction response regulator, receiver domain
Aminotransferase, class I/classII
GNAT domain

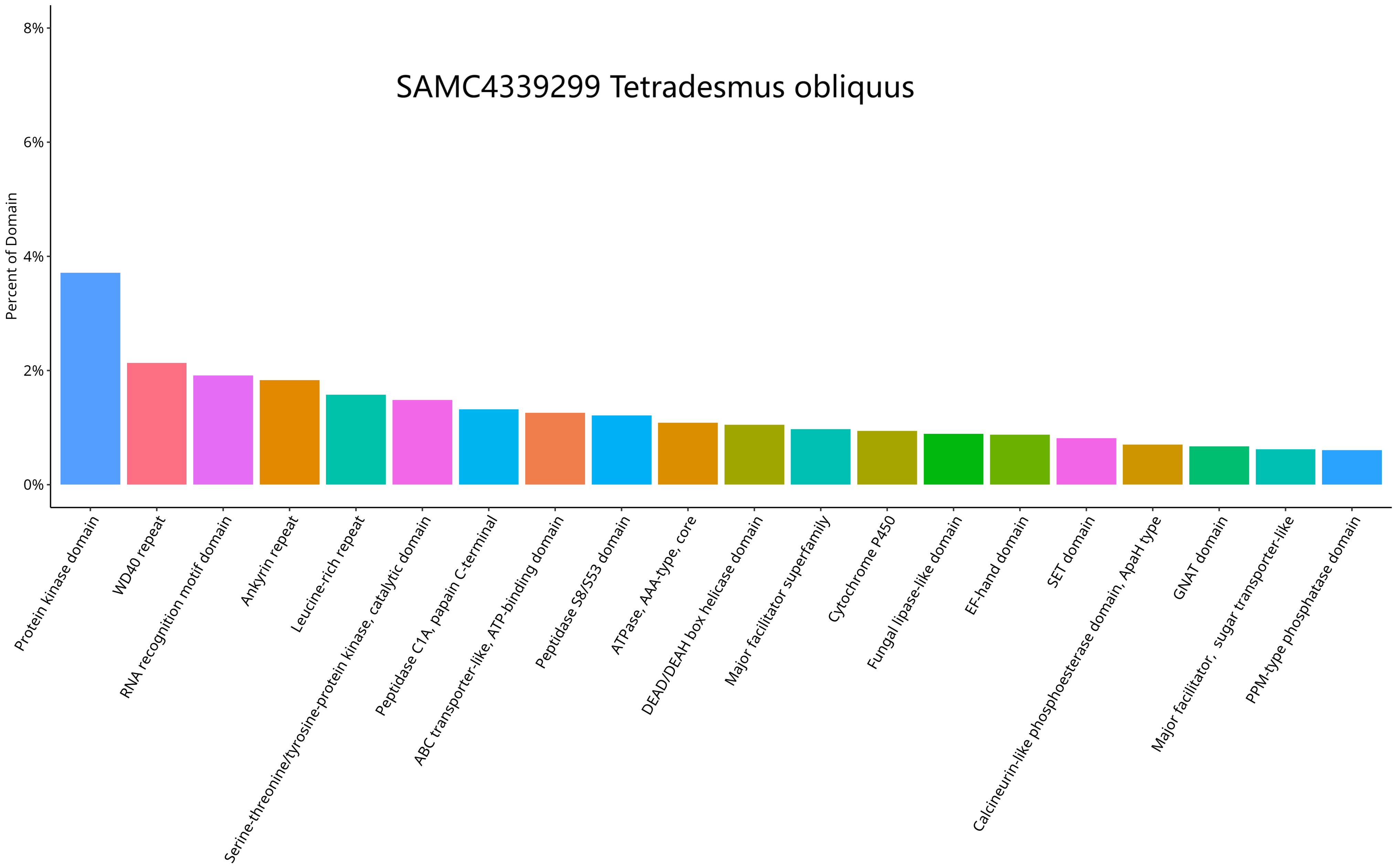
SAMC4339299 Tetradesmus obliquus
Percent of Domain
8%
6%
4%
2%
0%
Protein kinase domain
WD40 repeat
RNA recognition motif domain
Ankyrin repeat
Leucine-rich repeat
Serine-threonine/tyrosine-protein kinase, catalytic domain
Peptidase C1A, papain C-terminal
ABC transporter-like, ATP-binding domain
Peptidase S8/S53 domain
ATPase, AAA-type, core
DEAD/DEAH box helicase domain
Major facilitator superfamily
Cytochrome P450
Fungal lipase-like domain
EF-hand domain
SET domain
Calcineurin-like phosphoesterase domain, ApaH type
GNAT domain
Major facilitator, sugar transporter-like
PPM-type phosphatase domain

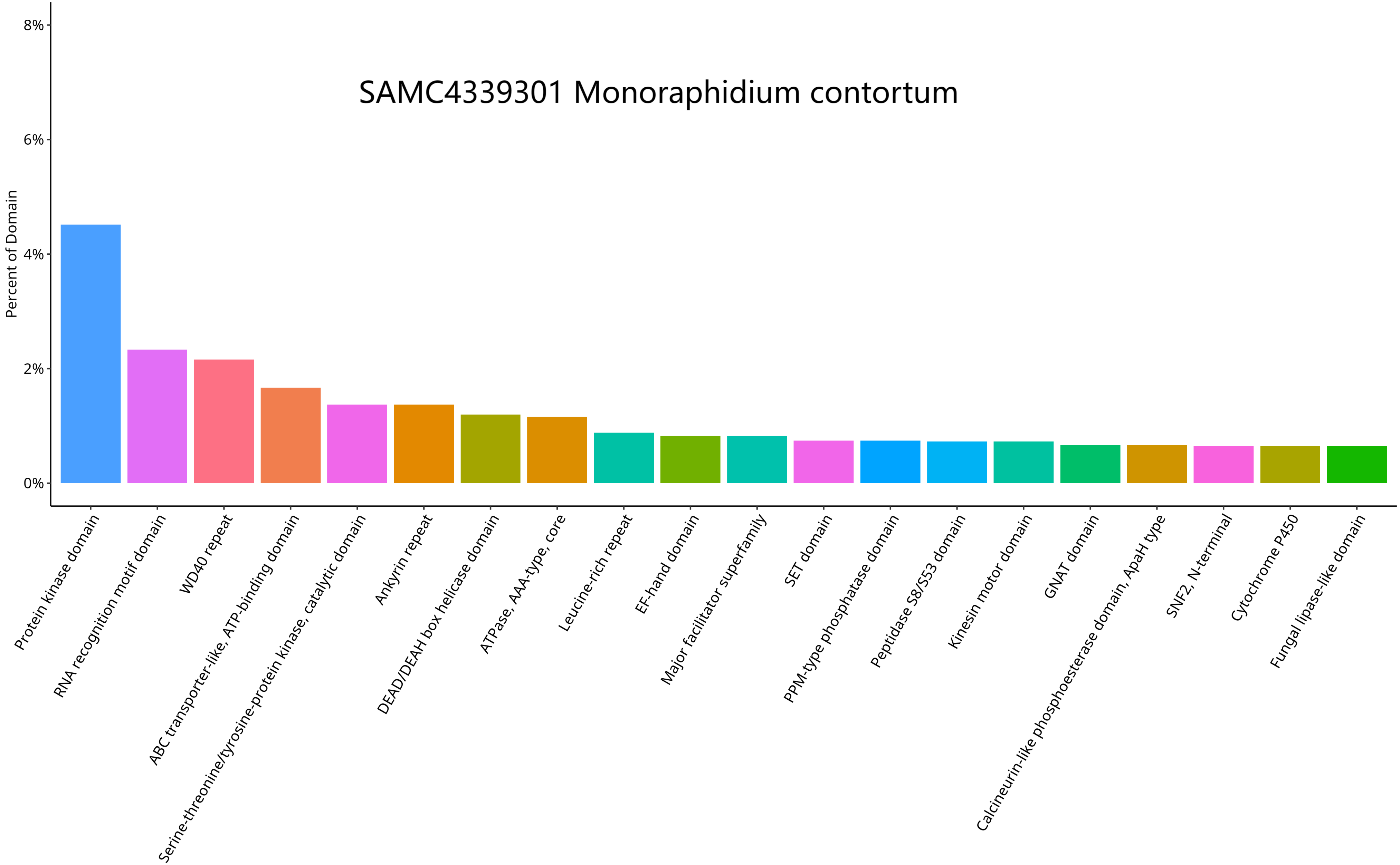
SAMC4339301 Monoraphidium contortum
Percent of Domain
8%
6%
4%
2%
0%
Protein kinase domain
RNA recognition motif domain
WD40 repeat
ABC transporter-like, ATP-binding domain
Serine-threonine/tyrosine-protein kinase, catalytic domain
Ankyrin repeat
DEAD/DEAH box helicase domain
ATPase, AAA-type, core
Leucine-rich repeat
EF-hand domain
Major facilitator superfamily
SET domain
PPM-type phosphatase domain
Peptidase S8/S53 domain
Kinesin motor domain
GNAT domain
Calcineurin-like phosphoesterase domain, ApaH type
SNF2, N-terminal
Cytochrome P450
Fungal lipase-like domain

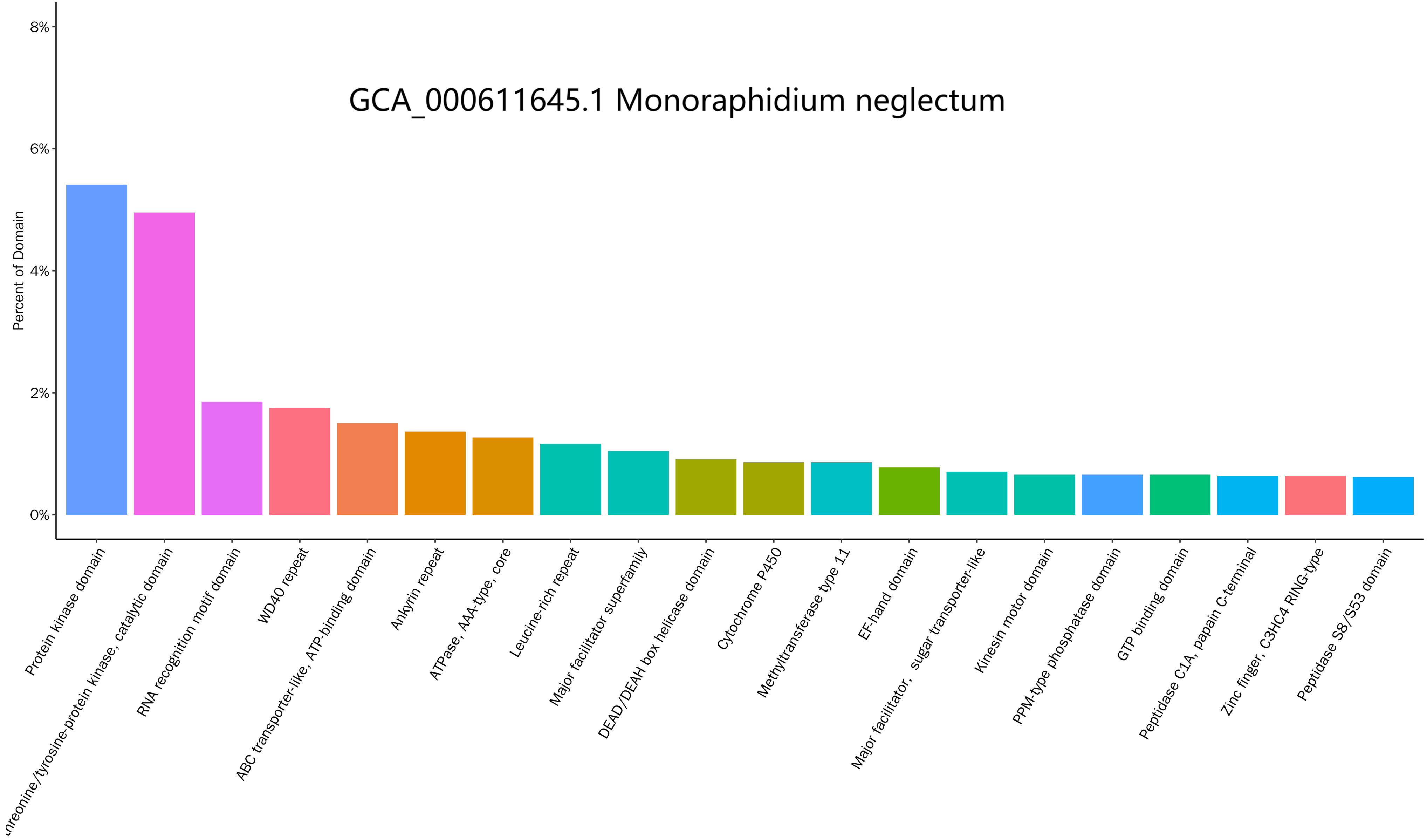
GCA_000611645.1 Monoraphidium neglectum
Percent of Domain
8%
6%
4%
2%
0%
Protein kinase domain
hreonine/tyrosine-protein kinase, catalytic domain
RNA recognition motif domain
WD40 repeat
ABC transporter-like, ATP-binding domain
Ankyrin repeat
ATPase, AAA-type, core
Leucine-rich repeat
Major facilitator superfamily
DEAD/DEAH box helicase domain
Cytochrome P450
Methyltransferase type 11
EF-hand domain
Major facilitator, sugar transporter-like
Kinesin motor domain
PPM-type phosphatase domain
GTP binding domain
Peptidase C1A, papain C-terminal
Zinc finger, C3HC4 RING-type
Peptidase S8/S53 domain

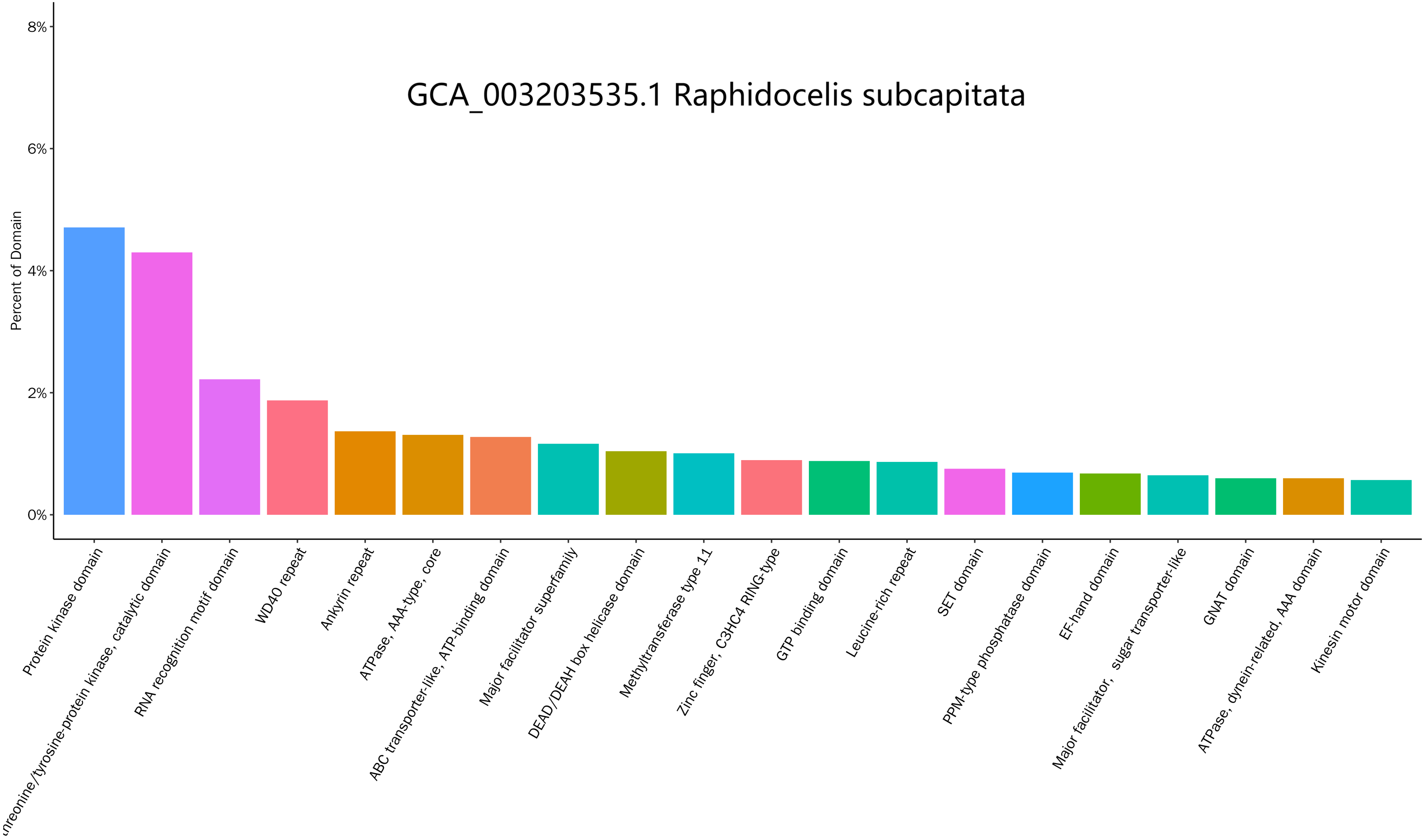
GCA_003203535.1 Raphidocelis subcapitata
Percent of Domain
8%
6%
4%
2%
0%
Protein kinase domain
hreonine/tyrosine-protein kinase, catalytic domain
RNA recognition motif domain
WD40 repeat
Ankyrin repeat
ATPase, AAA-type, core
ABC transporter-like, ATP-binding domain
Major facilitator superfamily
DEAD/DEAH box helicase domain
Methyltransferase type 11
Zinc finger, C3HC4 RING-type
GTP binding domain
Leucine-rich repeat
SET domain
PPM-type phosphatase domain
EF-hand domain
Major facilitator, sugar transporter-like
GNAT domain
ATPase, dynein-related, AAA domain
Kinesin motor domain

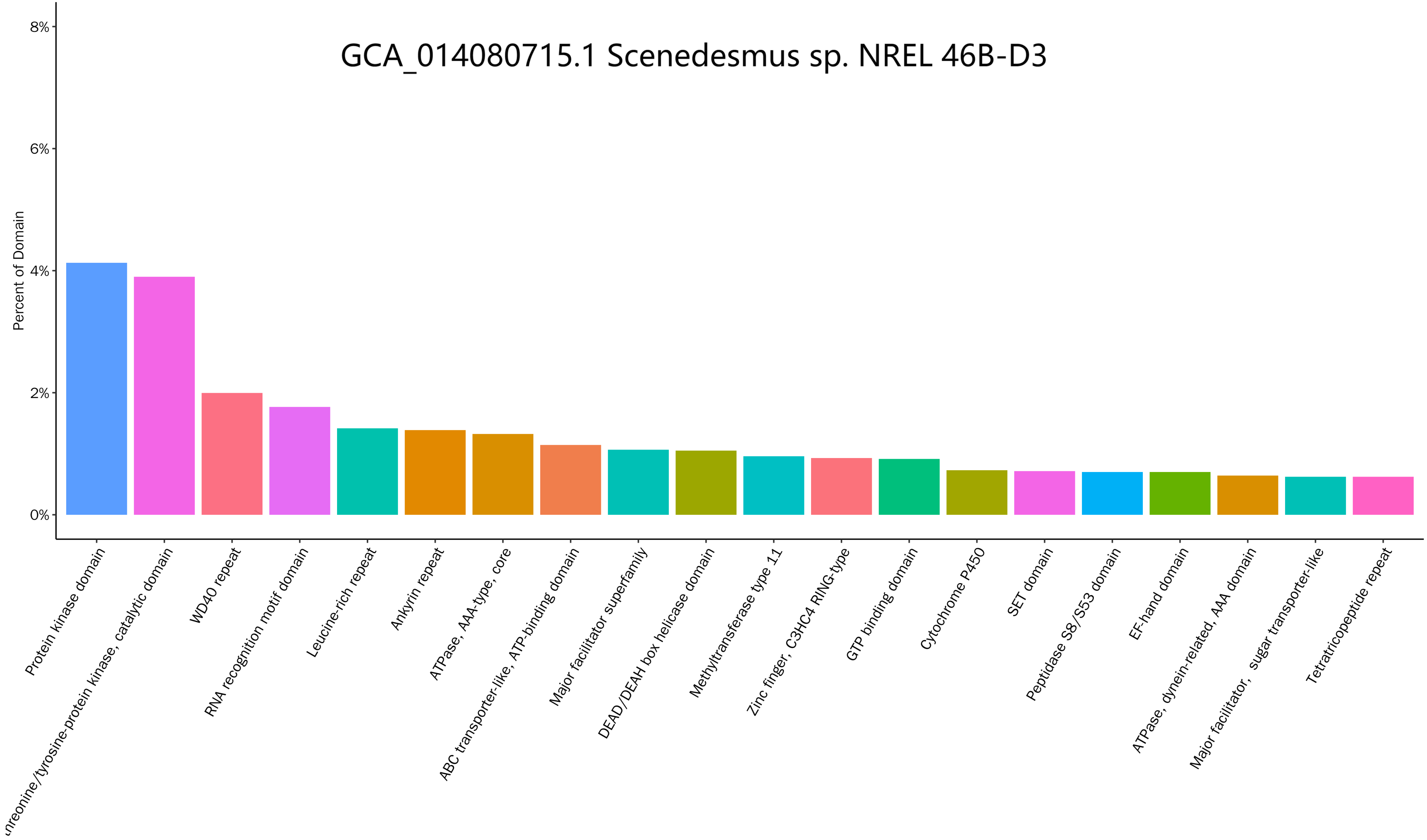

GCA_014080715.1 Scenedesmus sp. NREL 46B-D3
Percent of Domain
8%
6%
4%
2%
0%
Protein kinase domain
hreonine/tyrosine-protein kinase, catalytic domain
WD40 repeat
RNA recognition motif domain
Leucine-rich repeat
Ankyrin repeat
ATPase, AAA-type, core
ABC transporter-like, ATP-binding domain
Major facilitator superfamily
DEAD/DEAH box helicase domain
Methyltransferase type 11
Zinc finger, C3HC4 RING-type
GTP binding domain
Cytochrome P450
SET domain
Peptidase S8/S53 domain
EF-hand domain
ATPase, dynein-related, AAA domain
Major facilitator, sugar transporter-like
Tetratricopeptide repeat

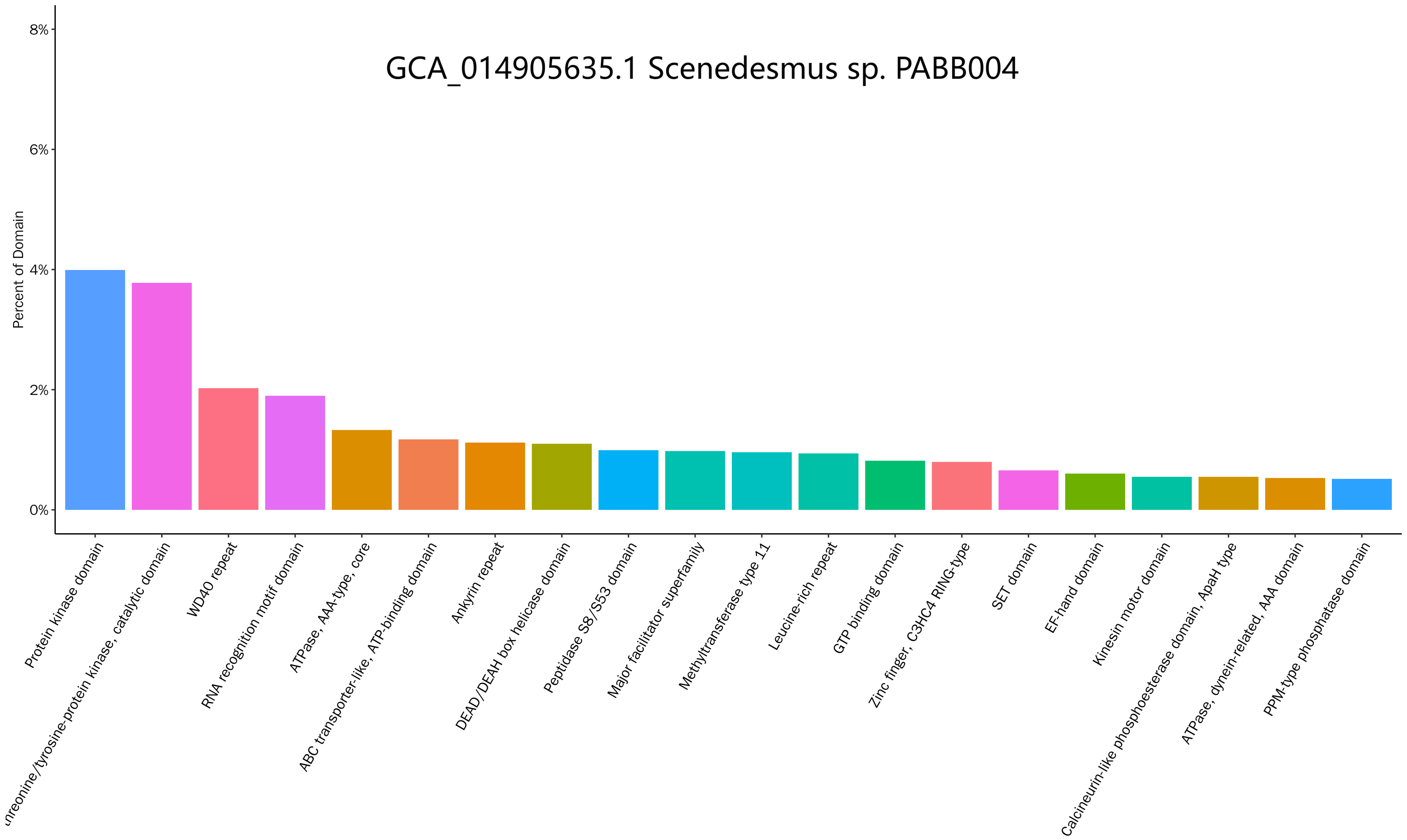
GCA_014905635.1 Scenedesmus sp. PABB004
Percent of Domain
8%
6%
4%
2%
0%
Protein kinase domain
reonine/tyrosine-protein kinase, catalytic domain
WD40 repeat
RNA recognition motif domain
ATPase, AAA-type, core
ABC transporter-like, ATP-binding domain
Ankyrin repeat
DEAD/DEAH box helicase domain
Peptidase S8/S53 domain
Major facilitator superfamily
Methyltransferase type 11
Leucine-rich repeat
GTP binding domain
Zinc finger, C3HC4 RING-type
SET domain
EF-hand domain
Kinesin motor domain
Calcineurin-like phosphoesterase domain, ApaH type
ATPase, dynein-related, AAA domain
PPM-type phosphatase domain

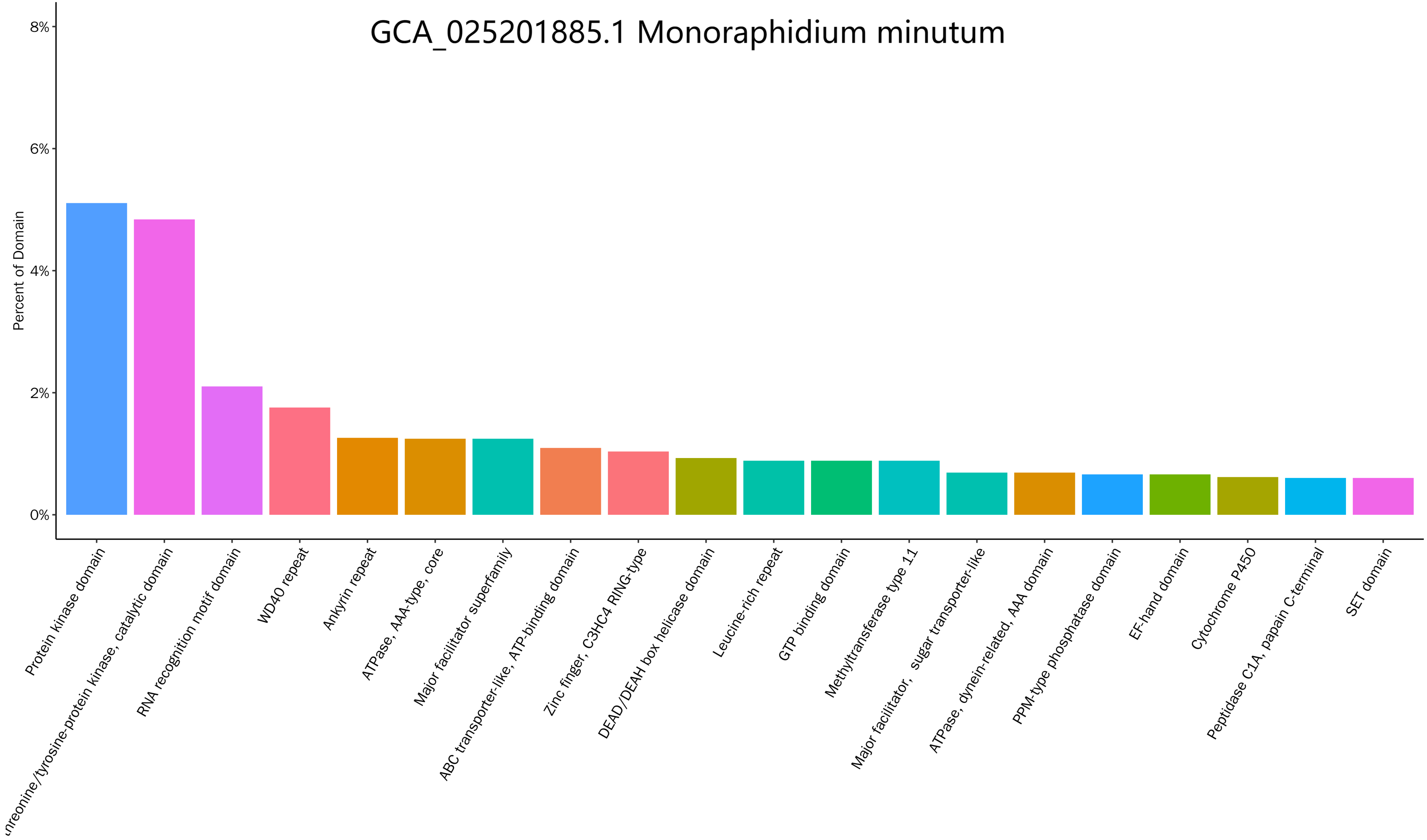

GCA_025201885.1 Monoraphidium minutum
Percent of Domain
8%
6%
4%
2%
0%
Protein kinase domain
...hreonine/tyrosine-protein kinase, catalytic domain
RNA recognition motif domain
WD40 repeat
Ankyrin repeat
ATPase, AAA-type, core
Major facilitator superfamily
ABC transporter-like, ATP-binding domain
Zinc finger, C3HC4 RING-type
DEAD/DEAH box helicase domain
Leucine-rich repeat
GTP binding domain
Methyltransferase type 11
Major facilitator, sugar transporter-like
ATPase, dynein-related, AAA domain
PPM-type phosphatase domain
EF-hand domain
Cytochrome P450
Peptidase C1A, papain C-terminal
SET domain

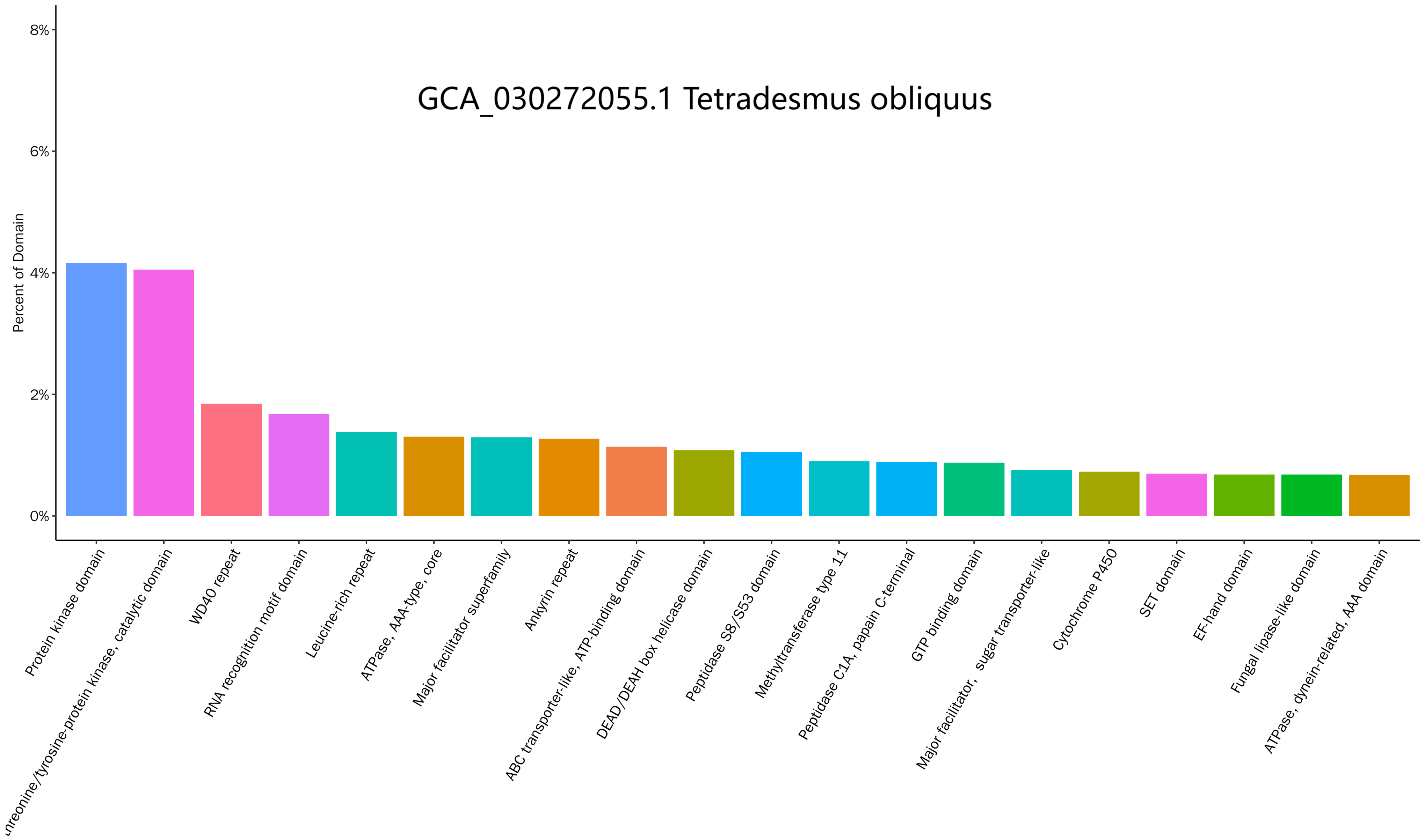
GCA_030272055.1 Tetradesmus obliquus
Percent of Domain
8%
6%
4%
2%
0%
Protein kinase domain
hreonine/tyrosine-protein kinase, catalytic domain
WD40 repeat
RNA recognition motif domain
Leucine-rich repeat
ATPase, AAA-type, core
Major facilitator superfamily
Ankyrin repeat
ABC transporter-like, ATP-binding domain
DEAD/DEAH box helicase domain
Peptidase S8/S53 domain
Methyltransferase type 11
Peptidase C1A, papain C-terminal
GTP binding domain
Major facilitator, sugar transporter-like
Cytochrome P450
SET domain
EF-hand domain
Fungal lipase-like domain
ATPase, dynein-related, AAA domain

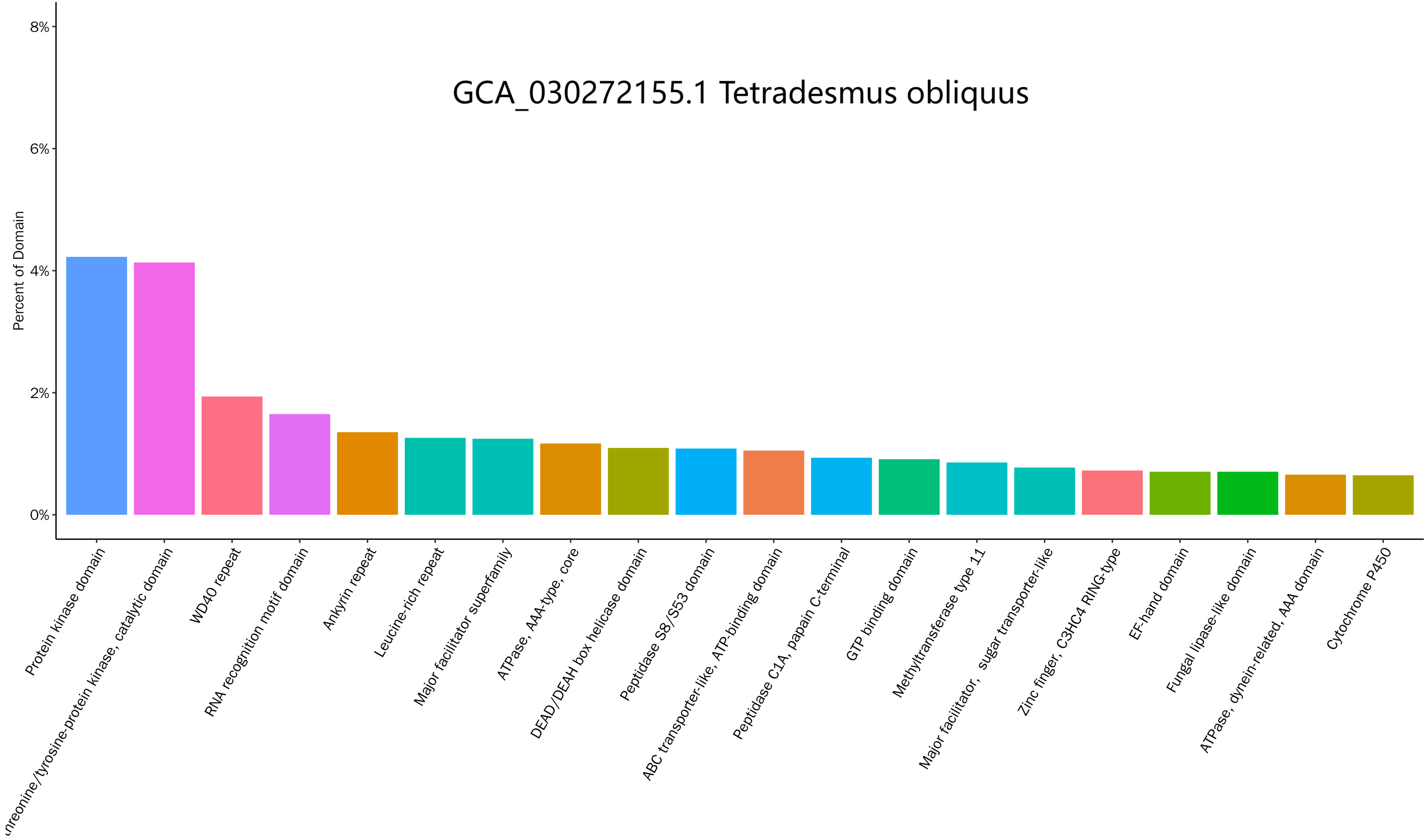

GCA_030272155.1 Tetradesmus obliquus
Percent of Domain
8%
6%
4%
2%
0%
Protein kinase domain
hreonine/tyrosine-protein kinase, catalytic domain
WD40 repeat
RNA recognition motif domain
Ankyrin repeat
Leucine-rich repeat
Major facilitator superfamily
ATPase, AAA-type, core
DEAD/DEAH box helicase domain
Peptidase S8/S53 domain
ABC transporter-like, ATP-binding domain
Peptidase C1A, papain C-terminal
GTP binding domain
Methyltransferase type 11
Major facilitator, sugar transporter-like
Zinc finger, C3HC4 RING-type
EF-hand domain
Fungal lipase-like domain
ATPase, dynein-related, AAA domain
Cytochrome P450
