# Supplementary material for: Comparative genomics and evolutionary analyses of Sphaeropleales
Source: Front Plant Sci. 2025 Oct 16;16:1534646. doi: 10.3389/fpls.2025.1534646 (PMC12571836; doi:10.3389/fpls.2025.1534646)
Supplement: Supplementary Figure 4 — The top 10 KEGG pathway categories of the 14 Sphaeropleales. [file DataSheet3.pdf]

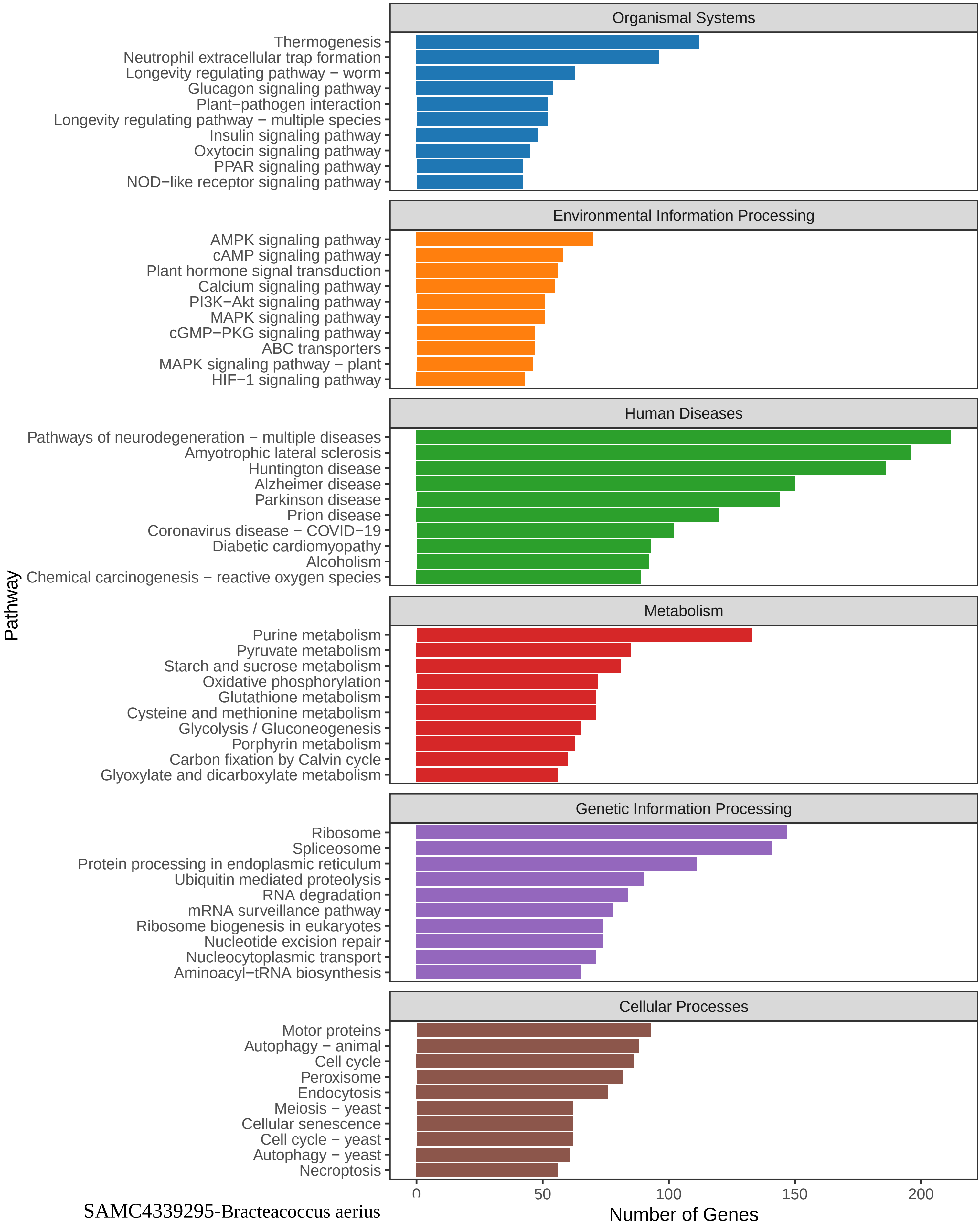


SAMC4339295-Bracteacoccus aerius

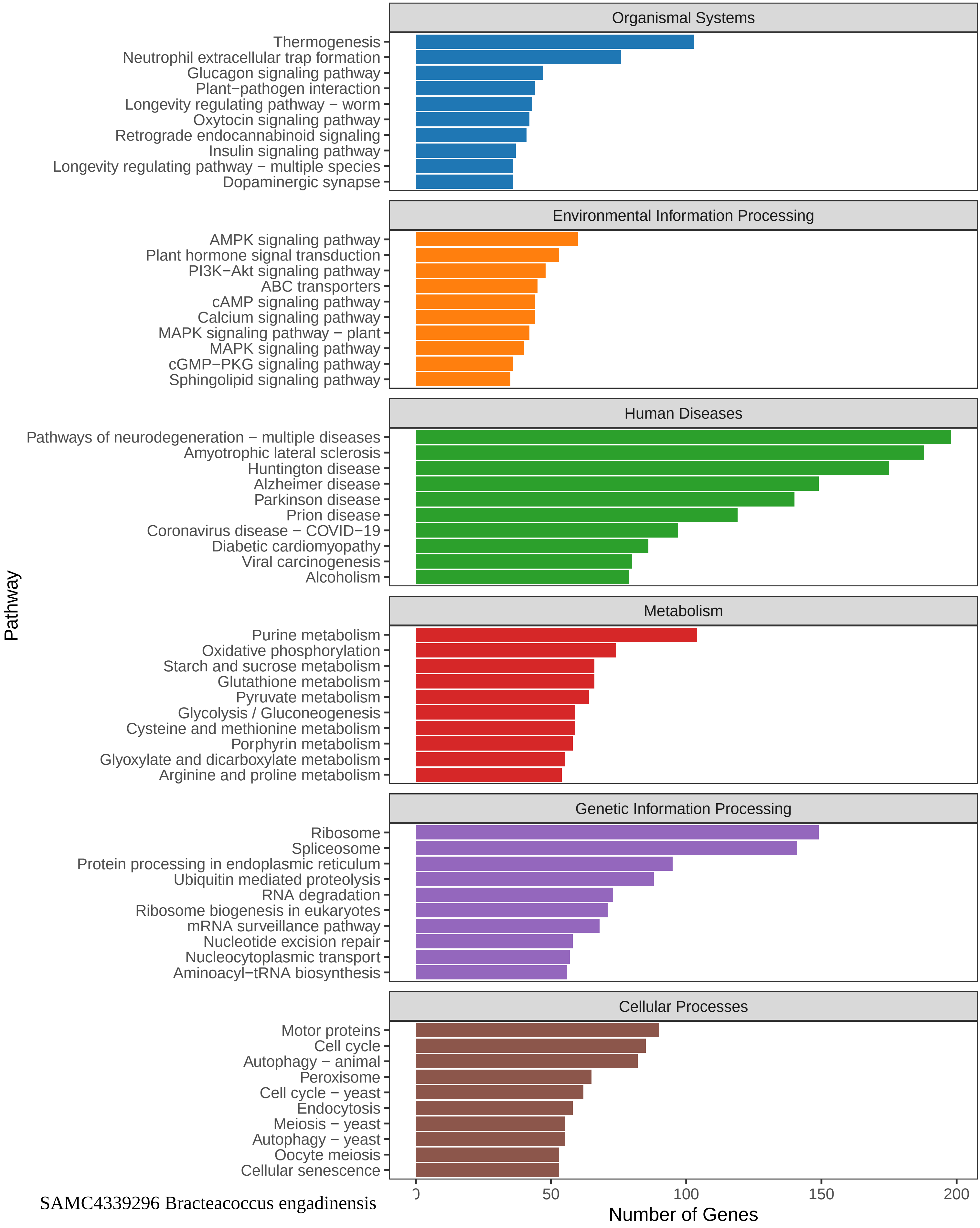


SAMC4339296 Bracteacoccus engadinensis

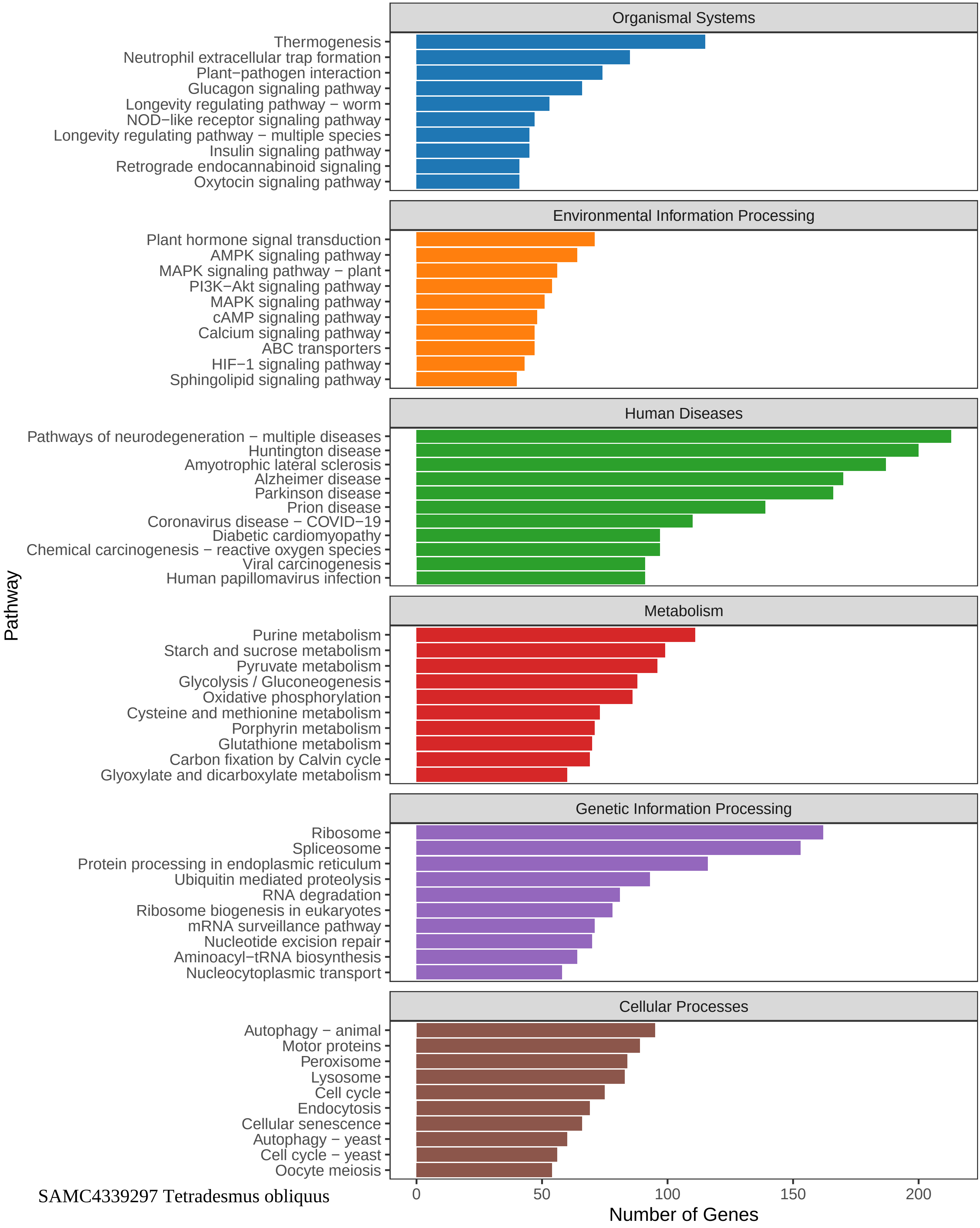


SAMC4339297 Tetradesmus obliquus

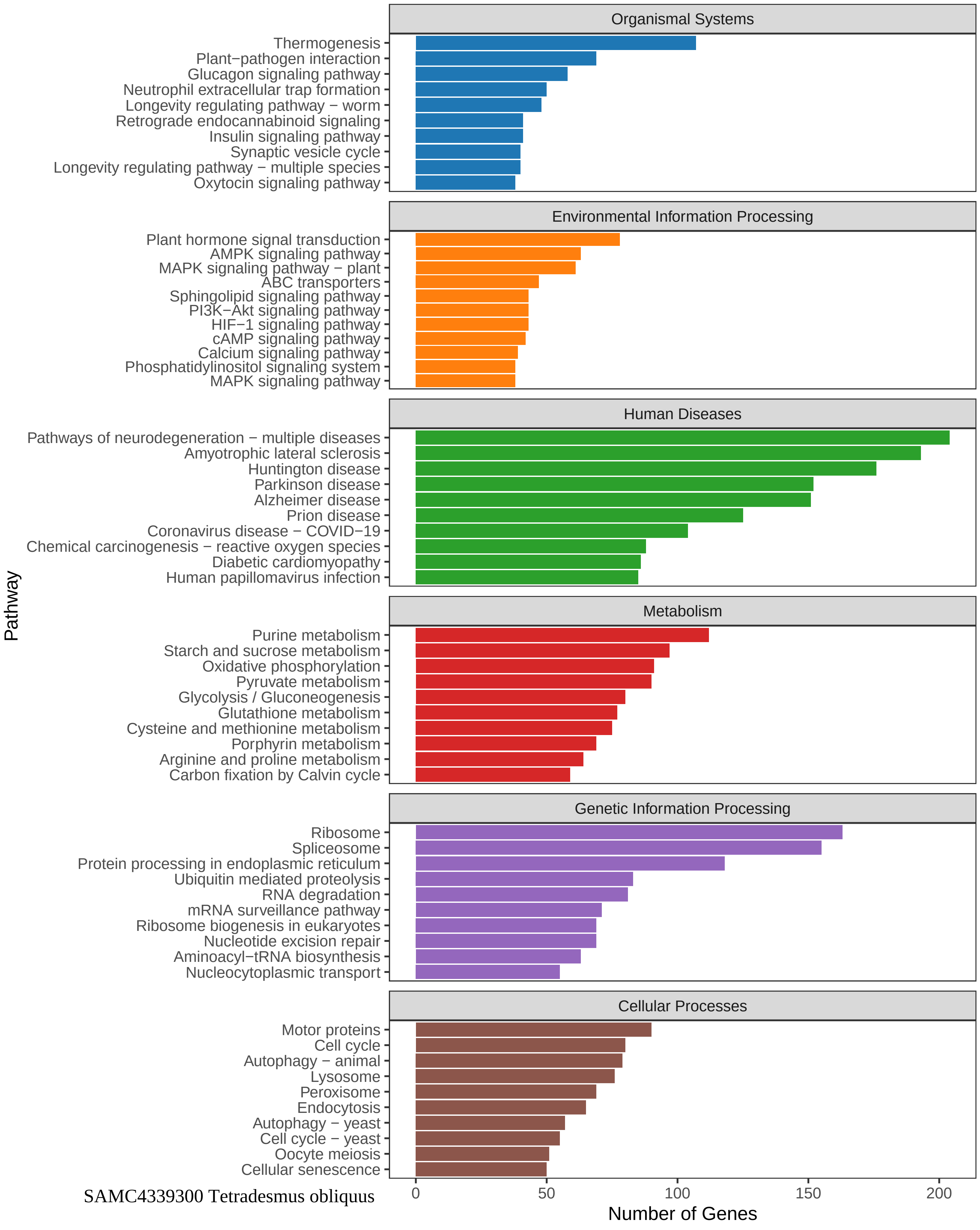


SAMC4339300 Tetradesmus obliquus

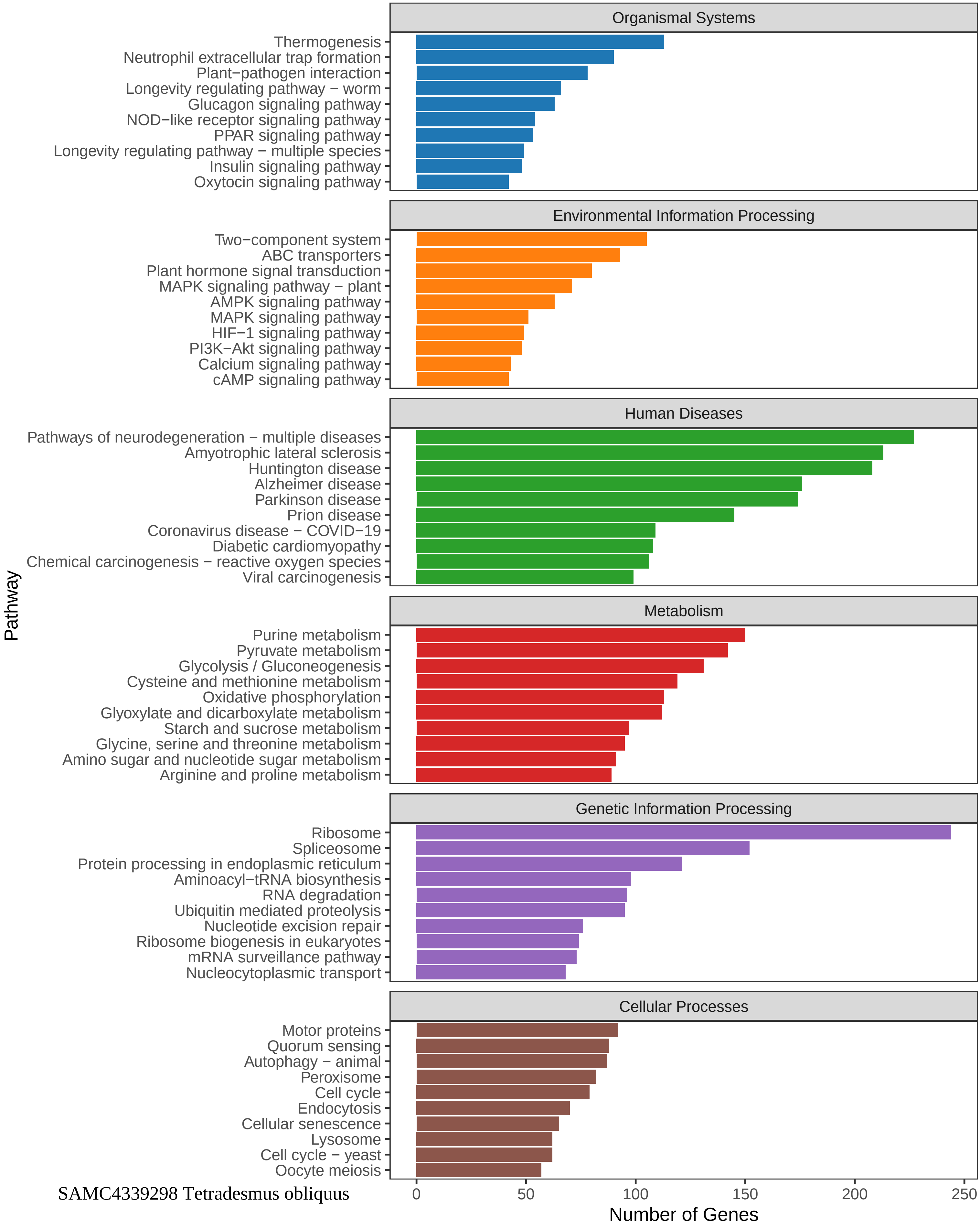


SAMC4339298 Tetradesmus obliquus

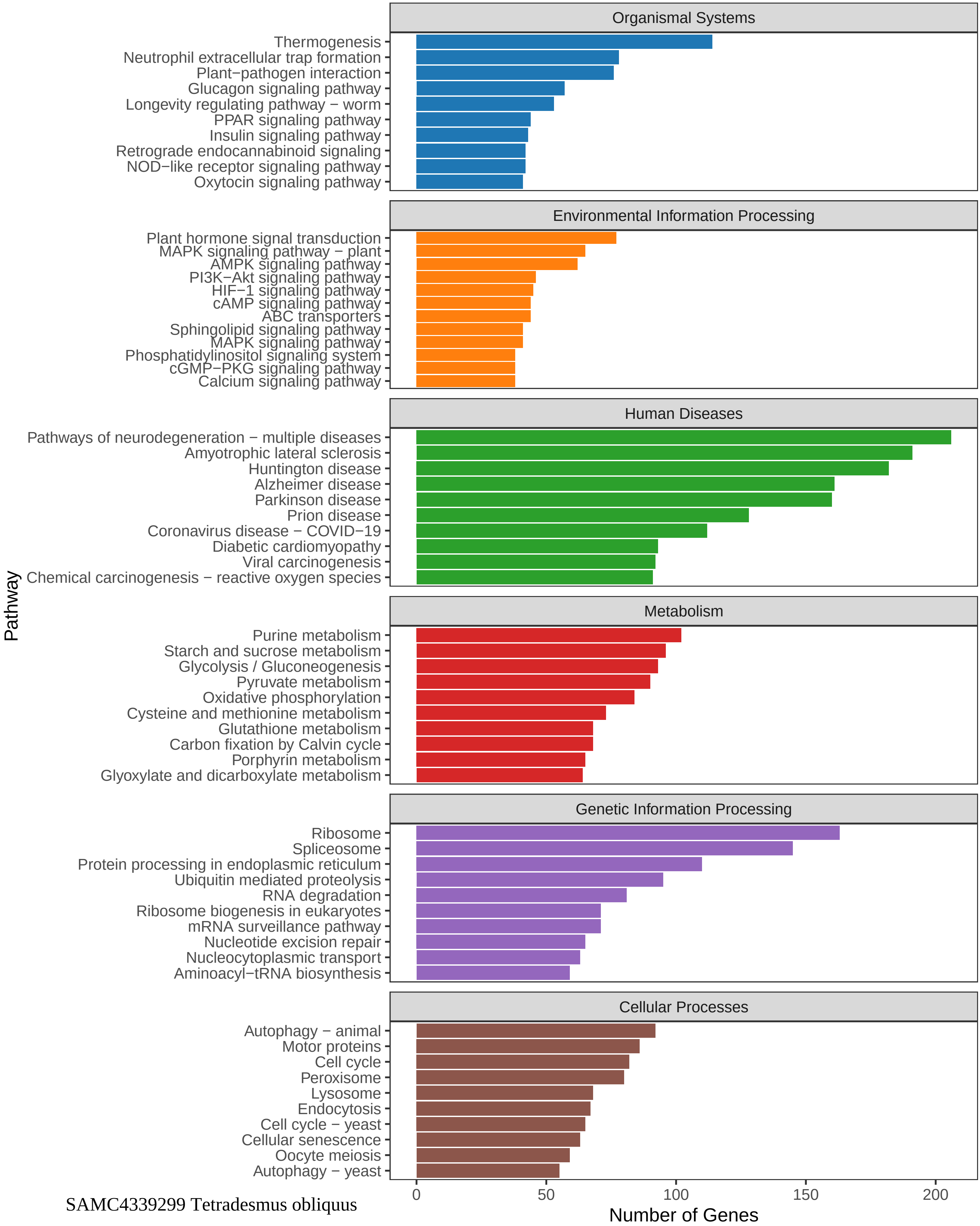


SAMC4339299 Tetradesmus obliquus

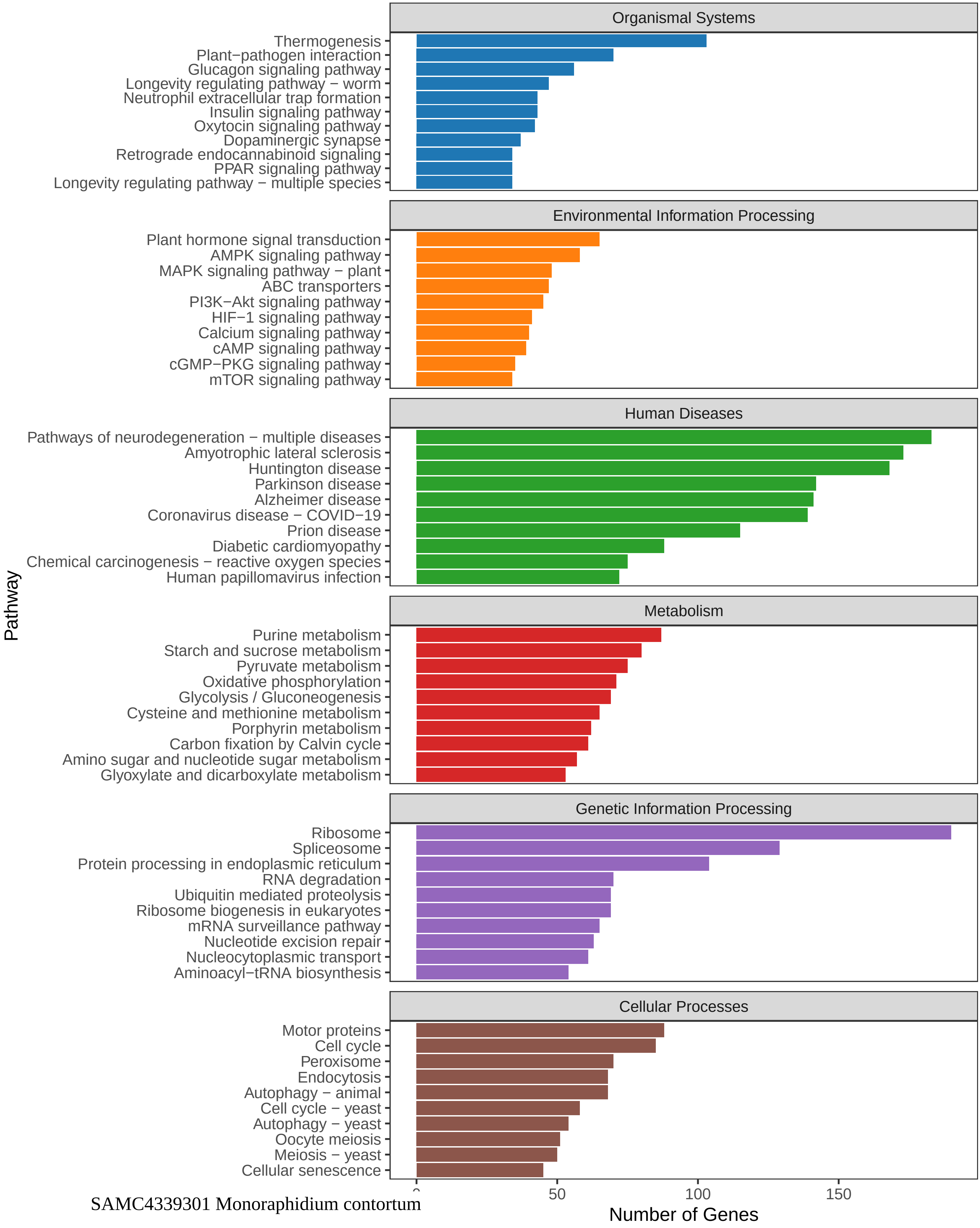


SAMC4339301 Monoraphidium contortum

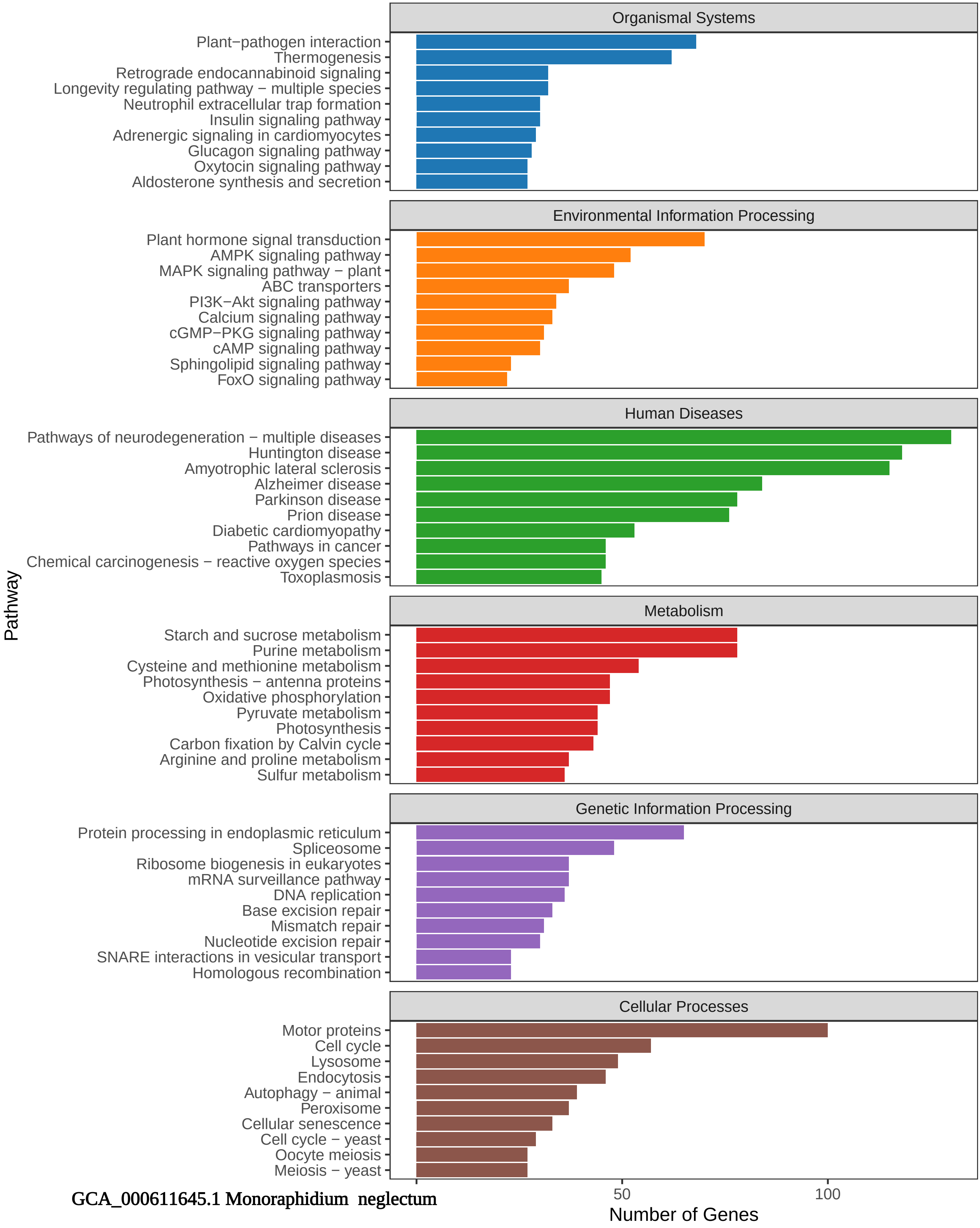


GCA_000611645.1 Monoraphidium neglectum

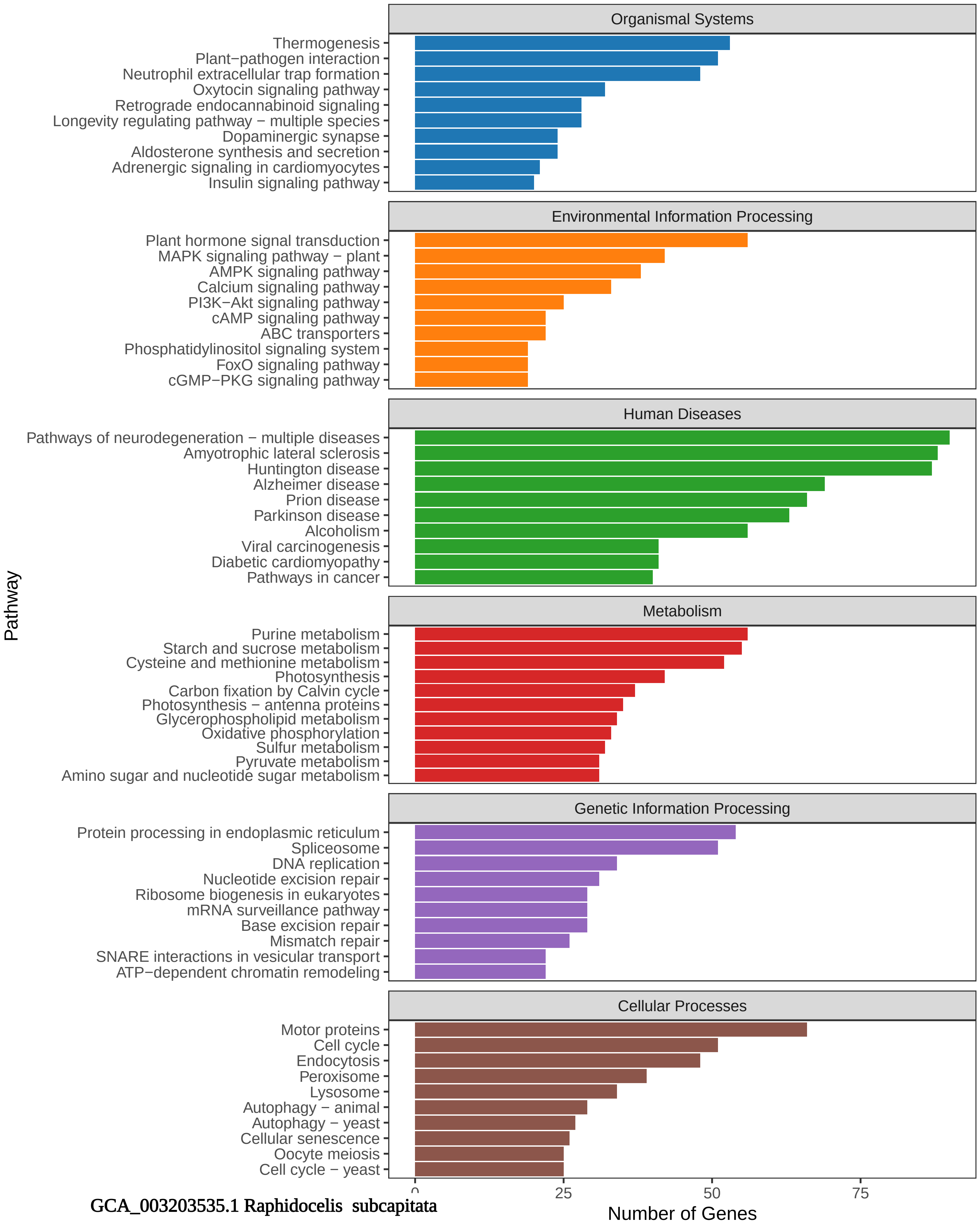


GCA_003203535.1 Raphidocelis subcapitata

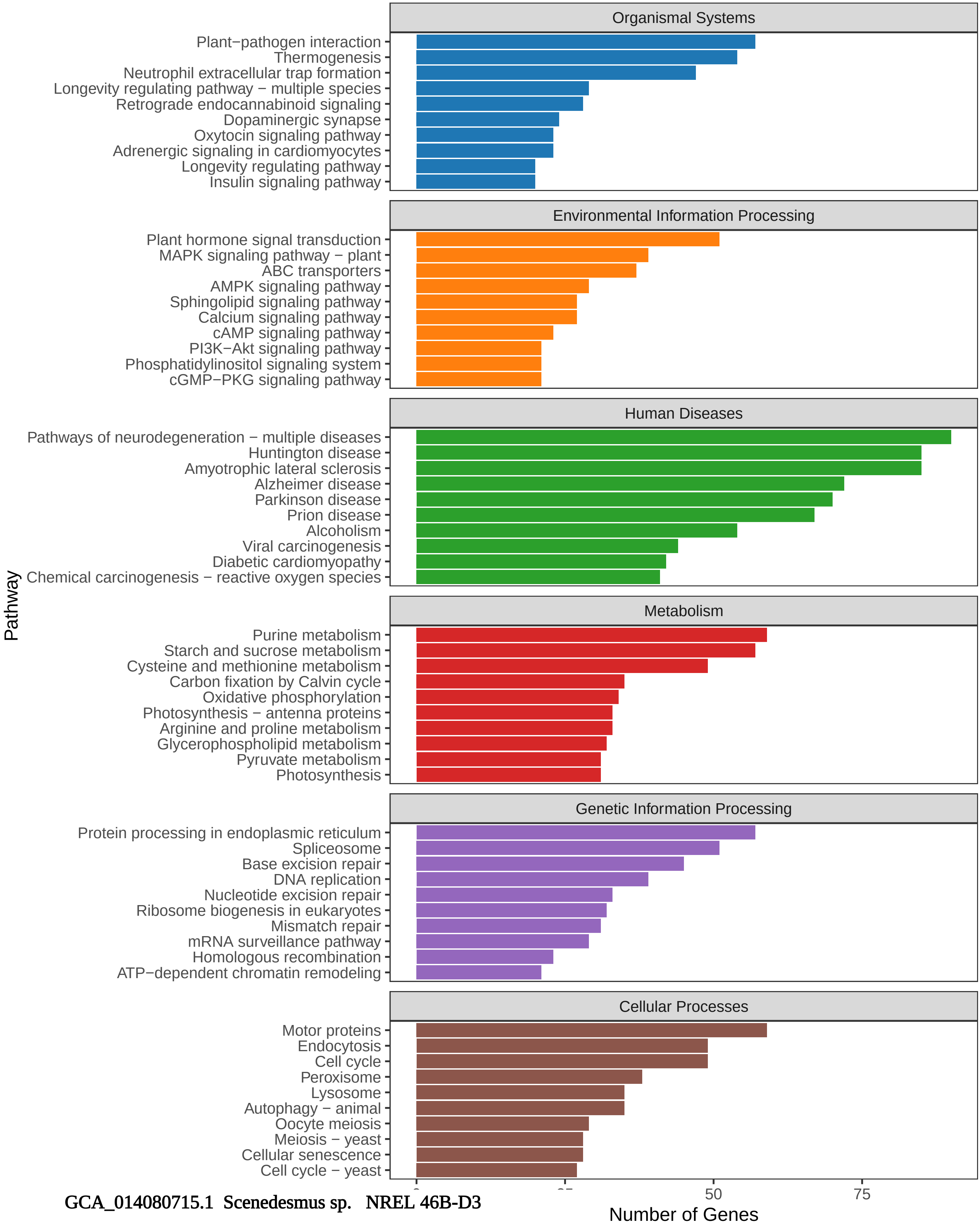


GCA_014080715.1 Scenedesmus sp. NREL 46B-D3

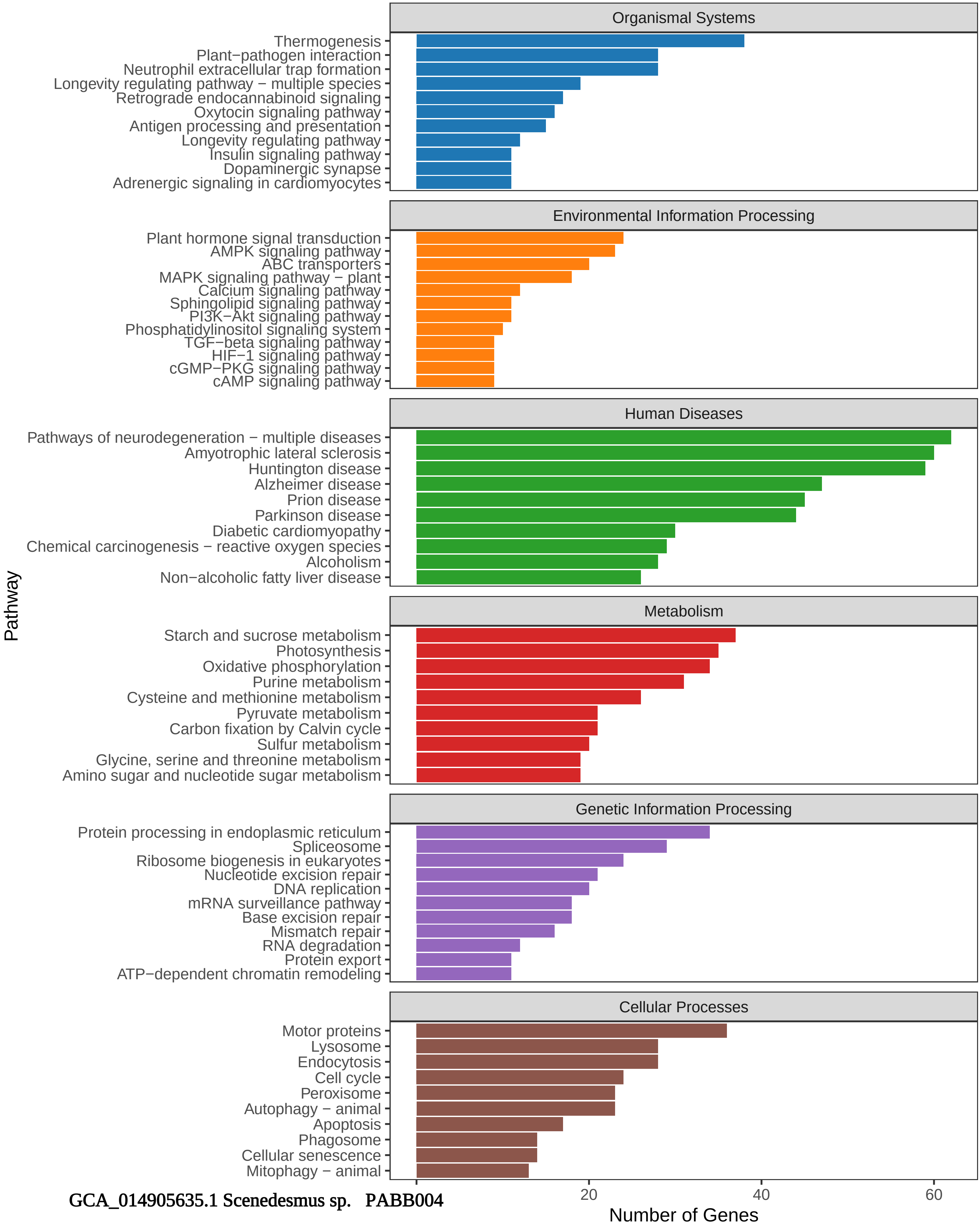


GCA_014905635.1 Scenedesmus sp. PABB004

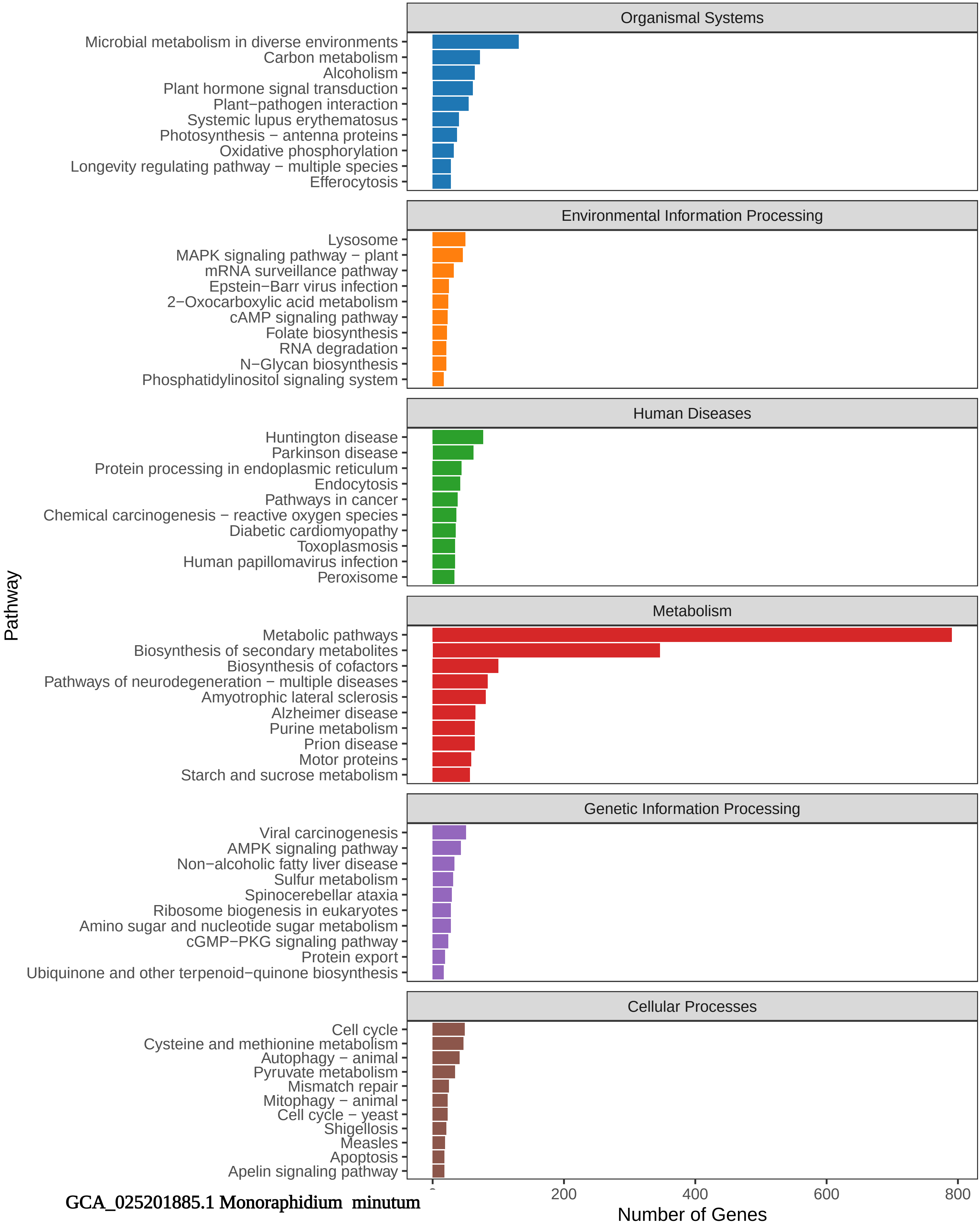


GCA_025201885.1 Monoraphidium minutum

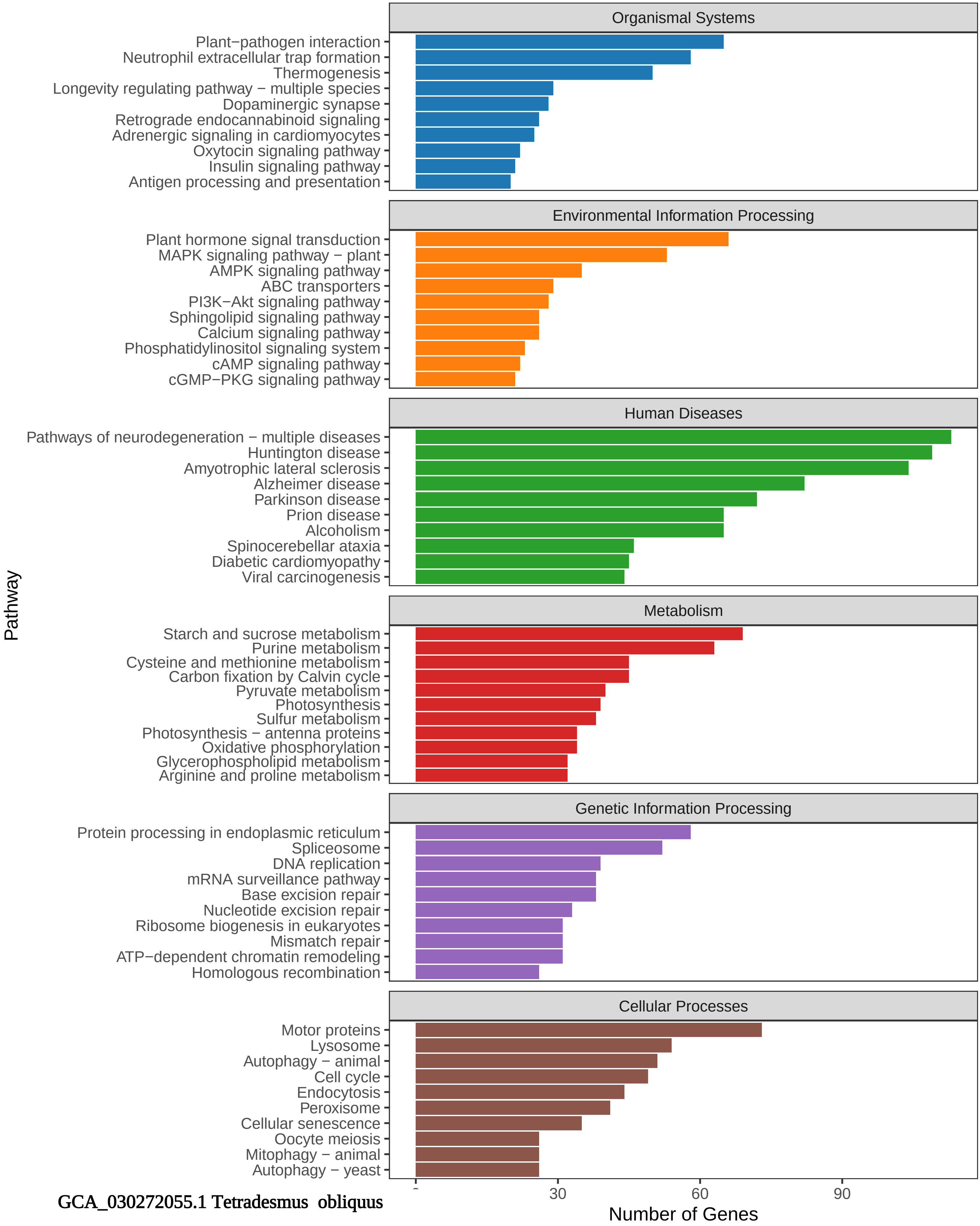


GCA_030272055.1 Tetradesmus obliquus

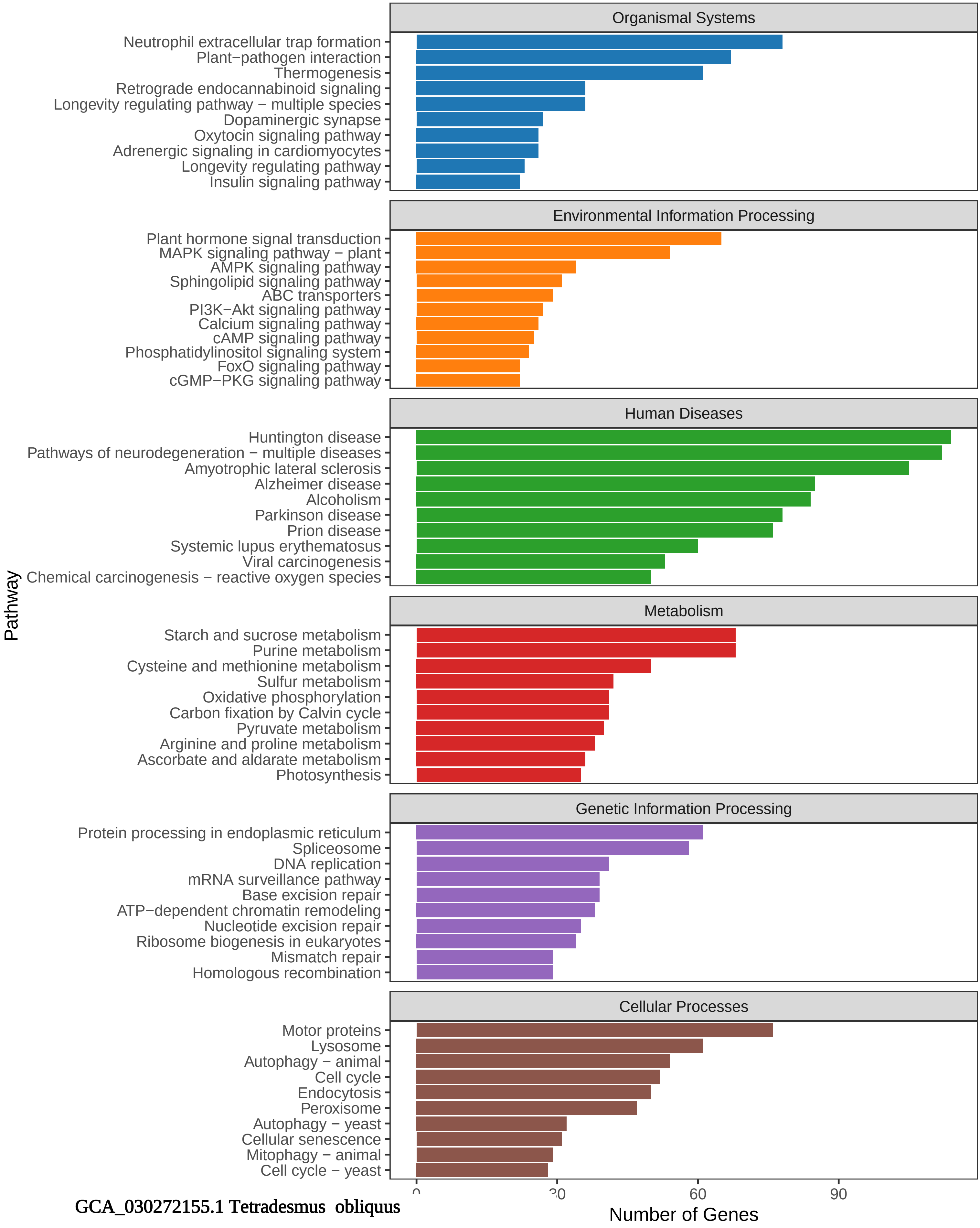


GCA_030272155.1 Tetradesmus obliquus
